# Improvement in the production of the human recombinant enzyme N-acetylgalactosamine-6-sulfatase (rhGALNS) in *Escherichia coli* using synthetic biology approaches

**DOI:** 10.1038/s41598-017-06367-w

**Published:** 2017-07-19

**Authors:** Luis H. Reyes, Carolina Cardona, Luisa Pimentel, Alexander Rodríguez-López, Carlos J. Alméciga-Díaz

**Affiliations:** 1 Institute for the Study of Inborn Errors of Metabolism, Faculty of Science, Pontificia Universidad Javeriana, Bogotá, Colombia; 2 Chemistry Department, Faculty of Science, Pontificia Universidad Javeriana, Bogotá, Colombia

## Abstract

Previously, we demonstrated production of an active recombinant human N-acetylgalactosamine-6-sulfatase (rhGALNS) enzyme in *Escherichia coli* as a potential therapeutic alternative for mucopolysaccharidosis IVA. However, most of the rhGALNS produced was present as protein aggregates. Here, several methods were investigated to improve production and activity of rhGALNS. These methods involved the use of physiologically-regulated promoters and alternatives to improve protein folding including global stress responses (osmotic shock), overexpression of native chaperones, and enhancement of cytoplasmic disulfide bond formation. Increase of rhGALNS activity was obtained when a promoter regulated under *σ* ^*s*^ was implemented. Additionally, improvements were observed when osmotic shock was applied. Noteworthy, overexpression of chaperones did not have any effect on rhGALNS activity, suggesting that the effect of osmotic shock was probably due to a general stress response and not to the action of an individual chaperone. Finally, it was observed that high concentrations of sucrose in conjunction with the physiological-regulated promoter *proU*_*mod*_ significantly increased the rhGALNS production and activity. Together, these results describe advances in the current knowledge on the production of human recombinant enzymes in a prokaryotic system such as *E. coli*, and could have a significant impact on the development of enzyme replacement therapies for lysosomal storage diseases.

## Introduction

Mucopolysaccharidosis IVA (MPS IVA, Morquio A disease, OMIM 253000) is a rare autosomal-recessive disease characterized by the deficiency of the lysosomal enzyme N-acetylgalactosamine-6-sulfate sulfatase (GALNS, EC 3.1.6.4) with an estimated incidence of 1:200,000 born alive^1, 2^. This enzyme is indispensable for the degradation of the glycosaminoglycans (GAGs) keratan sulfate and chondroitin-6-sulfate. The progressive accumulation of these GAGs within the lysosomes of multiple tissues such as ligaments, connective tissues, bone and cartilage, leads to the classical clinical manifestations of the disease including laxity of joints, skeletal dysplasia, hearing loss, corneal clouding, and pulmonary dysfunction, among others^3^.

Currently, the leading therapeutic option for MPS IVA is the enzyme replacement therapy (ERT) by using a recombinant enzyme produced in Chinese hamster ovaries (CHO) cells (elosulfase alfa)^4^. Although elosulfase alfa is a therapeutic option for MPS IVA patients, current limitations include^5^: (i) a limited effect on skeletal, corneal, and heart valvular tissues (ii) a short half-life of the enzyme and rapid clearance from the circulation (iii) immunological problems, and (iv) a high cost. An improved ERT with a long circulating enzyme and a bone-targeting enzyme have been proposed^6^, and a recombinant GALNS produced in other sources may potentially help to resolve some of the listed issues^7^.

The human GALNS complementary DNA is composed by 1569 bp, encoding a 522 amino acids peptide (herein known as precursor peptide). This protein undergoes several posttranslational modifications by the trafficking through the endoplasmic reticulum, Golgi apparatus and lysosome. These posttranslational modifications include the removal of the signal peptide (first 26 amino acids −3 kDa), the active-site activation by the formylglycine-generating enzyme, the addition of two N-glycosylations, and the proteolytic processing to obtain a ∼58 kDa mature enzyme formed by two polypeptides of 40 kDa and 18 kDa^8, 9^. In the case of the recombinant GALNS produced in *Escherichia coli* and *Pichia pastoris*, it was reported a similar processing to that observed for the enzyme produced in mammalian cells^9, 10^.

*E. coli* has been extensively used as the prokaryotic model organism for production of proteins with therapeutic and industrial interest^11–13^. The key problem in the production of human recombinant proteins in this bacterial host is related with the lack of post-translational modifications, as glycosylations, and poor protein folding, leading to loss of enzyme activity and the formation of insoluble protein aggregates^14^.

Previously, we demonstrated the production of a recombinant human GALNS enzyme (rhGALNS) in *E. coli* BL21(DE3) as a potential therapeutic alternative for MPS IVA^9, 15^. However, most of the produced protein was present as protein aggregates. Culture conditions were optimized, including inductor concentrations and temperature shifts, which maximized rhGALNS activity, but most of its production was present in the insoluble protein fraction^16^. Here we explored different approaches to increase the production and activity of rhGALNS in *E. coli*. These approaches included the use of physiologically-regulated promoters to modulate gene expression, induction of osmoprotectants as helpers in protein folding, the overexpression of chaperone proteins, the improvement in the formation of disulfide bonds, and the combination of the different approaches to explore additive effects.

## Results and Discussion

### Effect of physiologically-regulated promoters on rhGALNS activity

There are several factors contributing to the formation of inclusion bodies in prokaryotic systems at the transcriptional level, which are generally attributed to a high transcriptional rate^17^ and transcription leakiness due to poor promoter repression^18^. The staple prokaryotic promoter used in recombinant protein production in *E. coli* is the *lac* promoter, which has been widely utilized due to its ability to be straightforwardly controlled with isopropyl β-D-1-thiogalactopyranoside (IPTG) and tightly repressed via *lacIq* ^19^. Other promoters commonly used in recombinant protein production are thermal or chemically induced^20^. Previous studies demonstrated the production of active rhGALNS in *E. coli* BL21(DE3) using the *tac* promoter, a synthetic promoter created from the combination of promoters from the *trp* and *lac* operons, which is inducible by IPTG^21^. However, most of the rhGALNS was recovered from protein aggregates^9, 16^. We hypothesized that the expression of rhGALNS is either controlled poorly at the transcriptional level or it is highly overexpressed, which leads to the accumulation of rhGALNS as protein aggregates.

To facilitate the production process (i.e. avoiding the use of inductors) and considering a necessary strong regulation over rhGALNS expression, the promoters *osmY* and *proU*_*mod*_ were selected for gene expression. These two promoters have been described to be tightly controlled under *σ* ^*s*^, and to induce the gene transcription at the late-exponential and at the onset of stationary growth phases^22, 23^. The promoters *osmY* and *proU*_*mod*_ were cloned to obtain the plasmids pGEXosmY and pGEXproUmod (Table 1 and Supplementary Data). Subsequently, the plasmids were transformed into *E. coli* BL21(DE3) for rhGALNS production as described in the Materials and Methods section. The cells were growth in M9 minimal media, and *E. coli* BL21(DE3)/pGEX-5X-GALNSopt was used as control.

**Table 1:** List of plasmids used in this work.

Plasmid	Description	Selection Marker	Source of reference
pGEX-5X-GALNSopt	pGEX-5X-3 plasmid encoding the enzyme N-acetylgalactosamine-6-sulfatase codon-optimized for expression in *E. coli*	AmpR	This study
pGEXosmY	Modification of the pGEX-5X-GALNSopt plasmid where the promoter *tac* was replaced by the *osmY* promoter with its corresponding RBS	AmpR	This study
pGEXproUmod	Modification of the pGEX-5X-GALNSopt plasmid where the promoter *tac* was replaced by the *proU*_*mod*_ promoter with its corresponding RBS	AmpR	This study
pACYCDuet™-1	Bicistronic plasmid used as a control and to clone all the chaperones used in this study	CmR	Novagen, Merck Milipore
pDuet::GroS	Plasmids for the overexpression of the genes *groS, groL, groS* and *groL, dnaK, dnaJ, dnaK* and *dnaJ, ibpA, ibpB, ibpA* and *ibpB, dsbA, dsbB, dsbA* and *dsbB, grpE*, and *clpB* (respectively) driven by the *lac* promoter. Backbone pACYCDuet™-1	CmR	This study
pDuet::GroL			
pDuet::GroSL			
pDuet::DnaK			
pDuet::DnaJ			
pDuet::DnaKJ			
pDuet::IbpA			
pDuet::IbpB			
pDuet::IbpAB			
pDuet::DsbA			
pDuet::DsbB			
pDuet::DsbAB			
pDuet::GrpE			
pDuet::ClpB			

**Figure 1:**
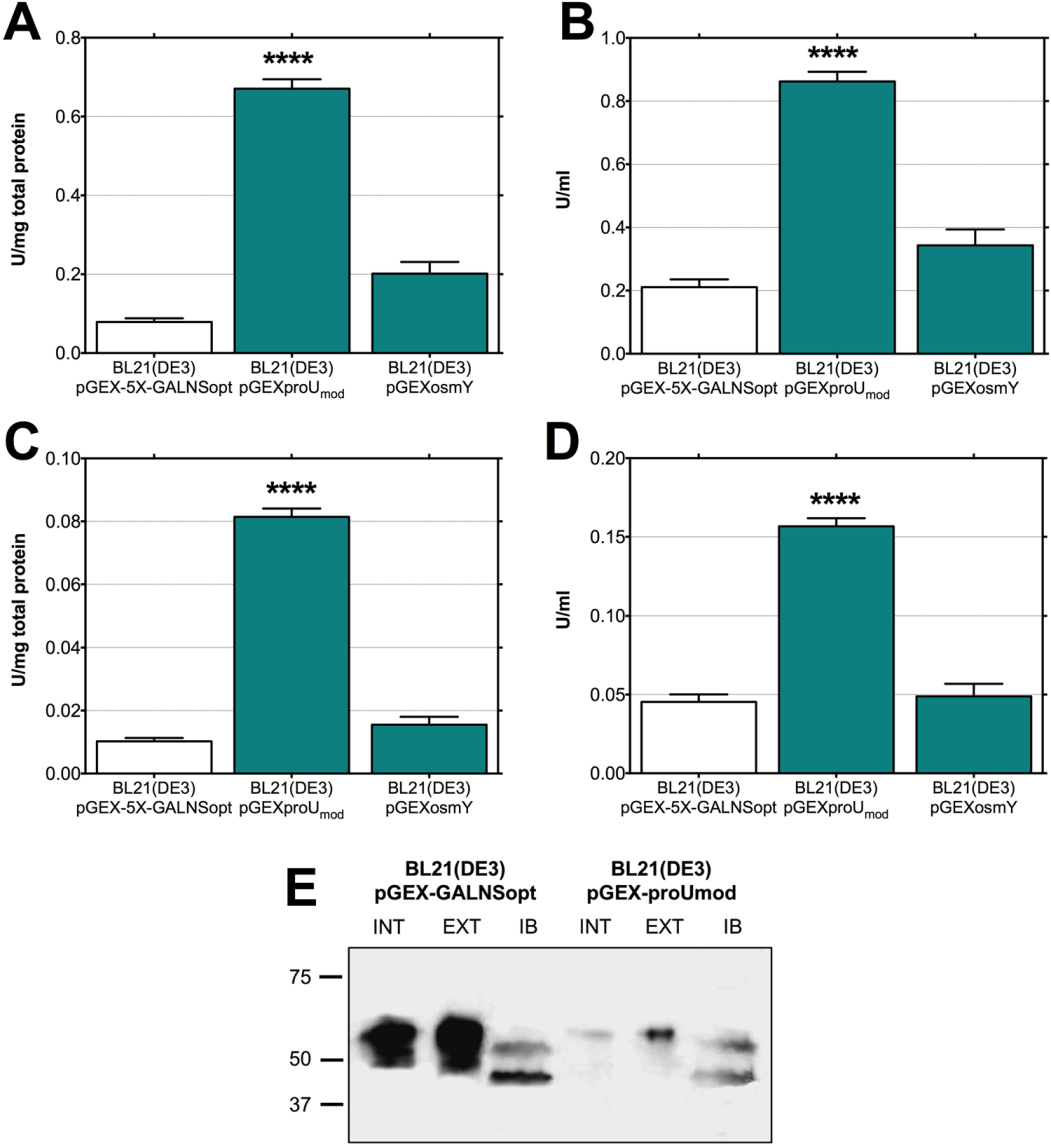
Production of rhGALNS in *E. coli* BL21(DE3) using different promoters. (**A**) Specific activity in the extracellular fraction. (**B**) Volumetric activity in the extracellular fraction. (**C**) Specific activity in the intracellular fraction. (**D**) Volumetric activity in the intracellular fraction. Enzyme activity was assessed 24 hours after induction (no induction was necessary in the case of the promoters studied). A Student’s t-test was performed to assess significance, where (****) corresponds to a p-value < 0.0001. Specific activities are expressed as U rhGALNS/(mg total protein) and volumetric activities as U/ml. (**E**) Western blotting analysis of *E. coli* extracts. Here we evaluated the effect of the promoters *tac* and *proU*_*mod*_ on the level expression of rhGALNS soluble protein at optimal (0.5 mM) IPTG concentration (in the case of *tac*) and 37 °C. Both soluble (intracellular [INT] and extracellular [EXT]) and protein aggregates (IB) fractions are shown. Samples from the intracellular fraction were normalized by the total protein amount, solubilized protein aggregates fractions were normalized by biomass, and the extracellular samples were normalized by culture volume. A non-cropped version of this figure can be found in the Supplementary Data.

As shown in Fig. 1, we did not observe a statistically significant increment in rhGALNS activity, in comparison to the levels observed using the strain BL21(DE3)/pGEX-5X-GALNSopt, when gene expression was driven by the *osmY* promotor, either intra- or extracellularly. This promoter has been reported as a weak promoter strongly regulated by *σ* ^*s*^ ^23^, in correspondence to the low enzyme activities obtained. On the other hand, rhGALNS activity increased significantly when the *proU*_*mod*_ promoter was used. The enzymatic activities obtained at the intracellular level (Fig. 1C and D) were 0.08 U/(mg total protein) and 0.16 U/mL, corresponding to increments of 695% and 245%, respectively, in comparison to the levels observed using the strain *E. coli* BL21(DE3)/pGEX-5X-GALNSopt. At the extracellular fraction, the enzyme activities, as shown in Fig. 1A and B, were 0.67 U/(mg total protein) and 0.86 U/mL of rhGALNS, equivalent to increments of 751% and 309%, respectively, in comparison to the levels observed using pGEX-5X-GALNSopt.

It is important to highlight that secretion of rhGALNS is a process mediated by the human native GALNS signal peptide. Extracellular secretion of rhGALNS in *E. coli* BL21(DE3), associated to the presence of a native signal peptide, was previously observed^16^. In the study, by using a similar plasmid construction to the one used in this report (*i.e*. GALNS with a native signal peptide downstream of the glutathione S-transferase [GST]), it was observed that removal of the signal peptide completely abolished secretion of the recombinant enzyme. Those results suggested that GALNS signal peptide is recognized by *E. coli* secretion machinery, even if it is located downstream of the GST peptide. In addition, removal of signal peptide severely affected the activation process of rhGALNS^16^.

In order to evaluate the presence of rhGALNS in different protein fractions, we performed a Western-blot using an anti-GALNS antibody, produced against the N-terminal region of the enzyme^16^. As expected, the antibody reacted with the high molecular mass rhGALNS polypeptide (~60 kDa) but it did not detect the small rhGALNS subunit (~18 kDa) (Fig. 1E). Although the sequence encoding for the GST tag was present in the recombinant plasmids and in-frame with GALNS sequence (see plasmids maps in the Supplementary Data), the expected 26 kDa size shift corresponding to this tag was not observed. In this sense, these results might suggest that either the fusion protein GST-rhGALNS was not produced or a post-translational processing of the recombinant enzyme might eliminate the GST tag. The absence of the fusion protein was validated using an affinity chromatography (see Supplementary Data). The results showed that GST tag was produced but it was not fused to recombinant enzyme. Although, further experiments are necessary to elucidate the reasons of the production of a protein lacking the GST tag, the Western-blot analysis, in addition to the enzyme activity results, confirm the production of the rhGALNS in *E. coli* BL21(DE3). Furthermore, the Western-blot analysis showed a lower rhGALNS production in all the protein fractions studied by using the *proU*_*mod*_ promoter, opposite to the production under the *tac* promoter (Fig. 1E). In addition, the ∼60 kDa rhGALNS precursor was observed in extra and intracellular fractions, while the precursor and the processed polypeptides were observed in the protein aggregates. These results might suggest an improvement in protein folding, since rhGALNS activity increases using the promoter *proU*_*mod*_ even when the corresponding enzyme amounts decrease. A densitometry analysis of the Western-blot for Fig. 1E was performed using the software ImageJ^24^ and allowed us to roughly estimate increments in protein folding of approximately 20X and 15X in the intracellular and extracellular fractions, respectively.

**Figure 2:**
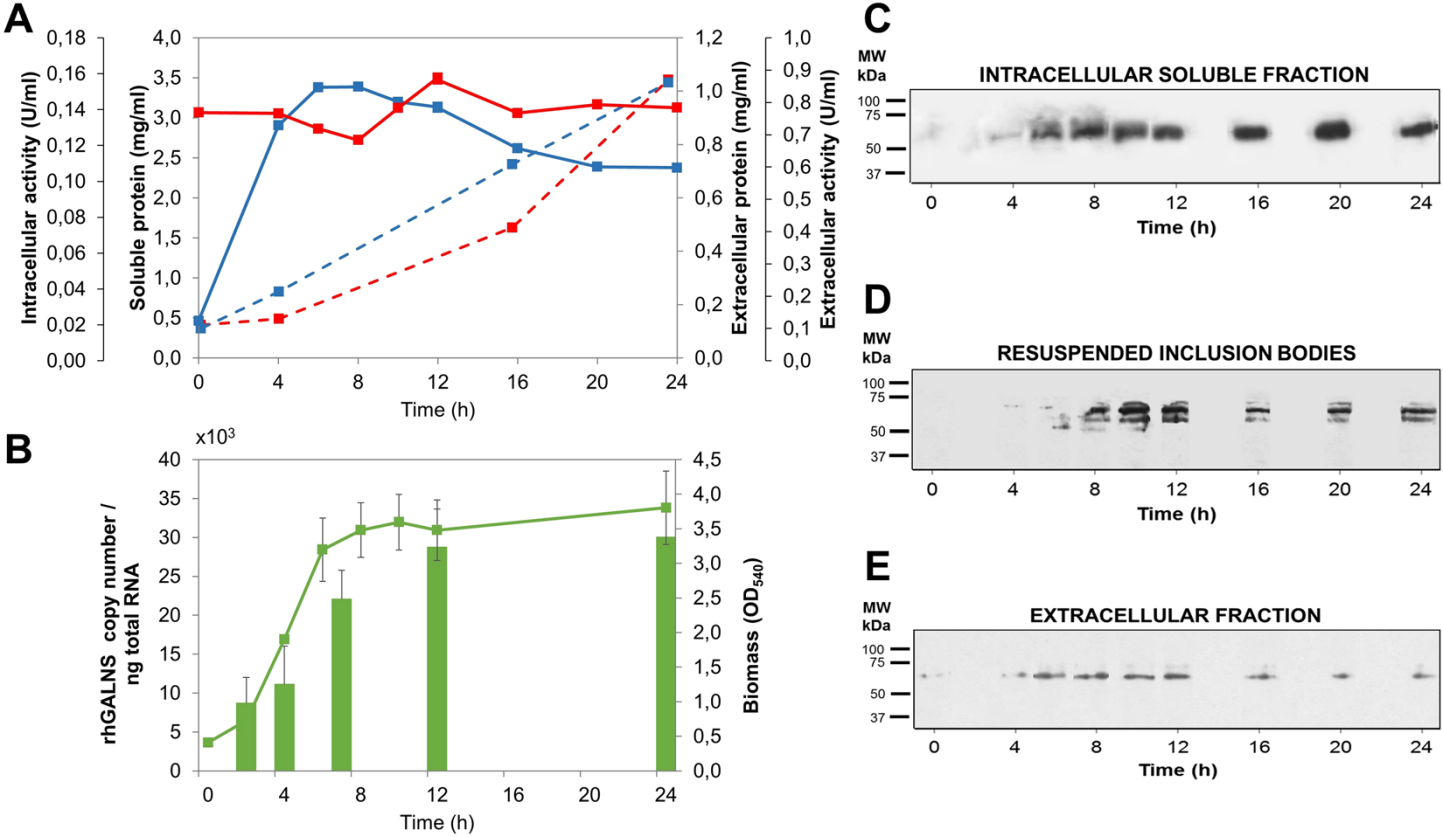
Dynamics of the expression of rhGALNS under the control of the promoter *proU*_*mod*_. (**A**) The solid lines represent the soluble protein quantitation expressed as mg/mL. Dashed lines indicate volumetric enzymatic activities expressed as U/mL. Blue lines show soluble proteins and enzyme activity of the intracellular fraction, whereas red lines are for the samples analyzed at the culture medium. These samples were analyzed at 0, 4, 6, 8, 10, 12, 16, 20 and 24 hours. (**B**) The green solid line shows the biomass measured as OD_540_ at 0, 2, 4, 6, 8, 10, 12 and 24 hours. Green bars represent the rhGALNS copy number per ng of total RNA, isolated at 2, 4, 7, 12 and 24 hours after inoculation. The expression profile of the promoter *proU*_*mod*_ shown corresponds to the profile expected of promoters regulated under *σ* ^*s*^. Western-blot analyses of rhGALNS at the, (**C**) Intracellular soluble fraction, (**D**) Solubilized protein aggregates fraction and (**E**) Extracellular fraction. All samples were collected at the same timestamps analyzed in part (**A**) Samples from the intracellular fraction were normalized by the total protein amount, solubilized protein aggregates fractions were normalized by biomass, and the extracellular samples were normalized by culture volume. A non-cropped version of this figure can be found in the Supplementary Data.

The dynamics of rhGALNS expression under the promoter *proU*_*mod*_ was determined as shown in Fig. 2. Different parameters were monitored in 100 ml culture during the first 24 h of cultivation including the intracellular and extracellular total soluble protein, the intracellular and extracellular rhGALNS activity, amounts of rhGALNS mRNA, and the protein amount of rhGALNS in the different protein fractions. After the first 6 h of cultivation the cells decelerate their growth, entering in stationary phase, as evidenced in the steady amounts of intracellular soluble protein (Fig. 2). At this point, the expression of rhGALNS increased significantly, supported by the elevation of the rhGALNS transcripts. The expression profile is an expected gene expression dynamics profile of promoters governed by *σ* ^*s*^ regulation, as demonstrated in the study of *rpoS*-dependent gene promoters expressing the gene *lacZ* ^22^ and the study of stationary-phase promoters using GFP^25^. This increment in rhGALNS transcription leads to an increase in the corresponding protein levels, as seen in the Western-blot (it increased in an accelerated manner in the first few hours after entering stationary phase), and consequently an increment in the rhGALNS activity. An increasing concentration of rhGALNS in the solubilized protein aggregates was observed, indicating that reduced expression is not sufficient to ensure proper protein folding (Fig. 2). Analysis of the extracellular fraction exhibits a small variation of protein along with an increasing activity of rhGALNS, suggesting a higher level of protein folding. However, these findings are currently under further study. Additionally, even after the reduction of rhGALNS gene expression under the control of *proU*_*mod*_, we still observed a different expression pattern in protein aggregates (Fig. 2C). This phenomenon could be due to poor protein processing or traffic saturation as shown in other studies^26–28^.

These results demonstrate the importance of controlling gene expression in the production of rhGALNS, not only in terms of promoter strength, but also regarding transcriptional dynamics and regulation. These results also support the versatility of using physiologically-controlled promoters for protein expression in the sense that it will facilitate the controllability of production processes and reduce the process costs since no inducer is required.

### Induction of osmoprotectants

Two strategies to improve the amount of soluble recombinant proteins were used in this study, which involve increasing the concentration of osmolytes and the overexpression of chaperones^14, 29^. Overproduction of bacterial chaperones, some of which actively drive folding processes whereas others prevent protein aggregation, can be obtained by different extracellular stresses such as heat-shock^30^ or osmotic shock^31^.

**Figure 3:**
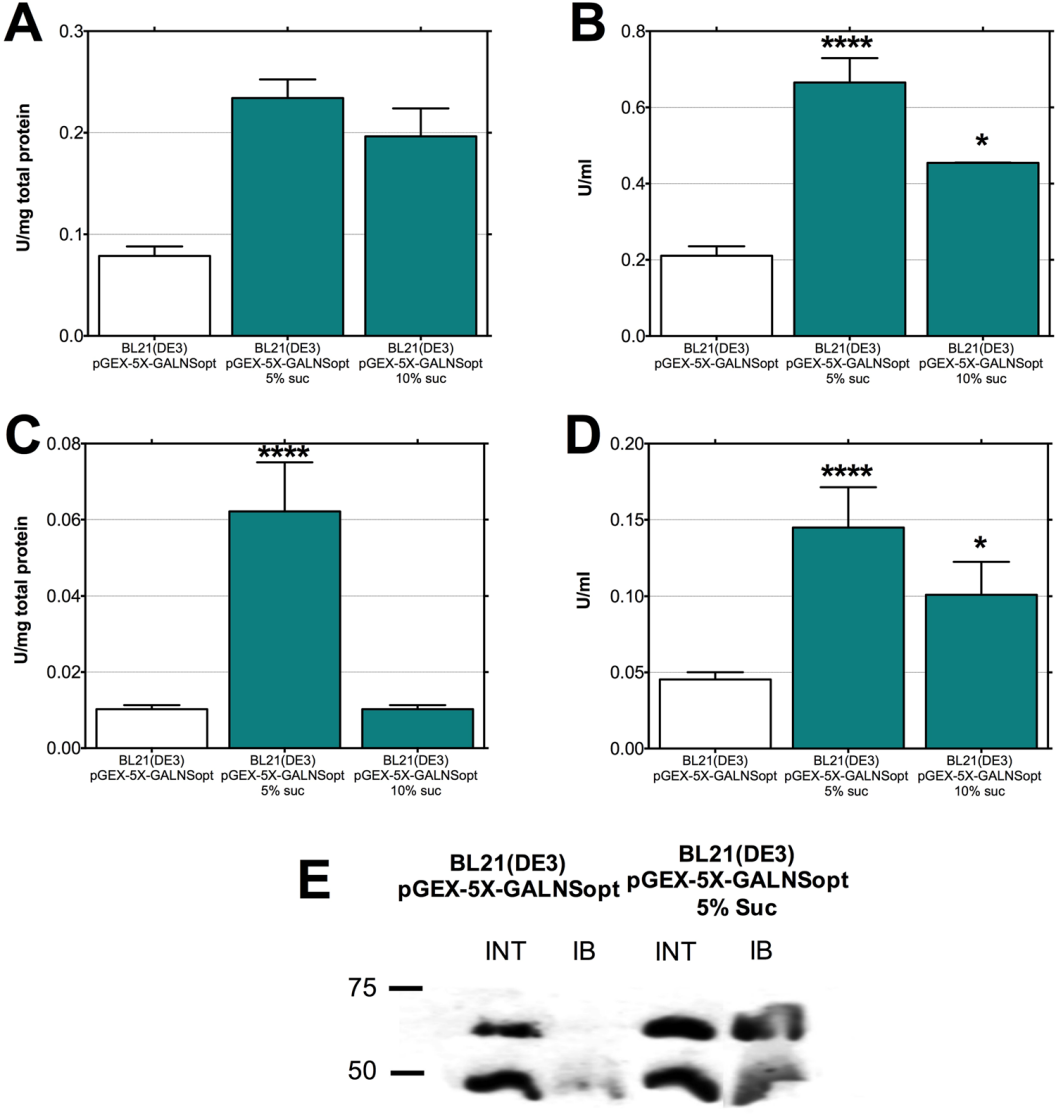
Production of rhGALNS in *E. coli* BL21(DE3) with cultures exposed to high osmotic stress. (**A**) Specific activity in the extracellular fraction. (**B**) Volumetric activity in the extracellular fraction. (**C**) Specific activity in the intracellular fraction. (**D**) Volumetric activity in the intracellular fraction. Enzyme activity was assessed 24 hours after induction. A Student’s t-test was performed to assess significance, where (*) corresponds to a p-value < 0.05 and (****) p-value < 0.0001. Specific activities are expressed as U rhGALNS/(mg total protein) and volumetric activities as U/ml. (**E**) Western-blot of rhGALNS levels in the intracellular and insoluble protein fractions using high osmotic stress, with production controlled by the promoter *tac* using optimal (0.5 mM) IPTG concentration. The results showed a significant increase of rhGALNS protein in both, intracellular and solubilized protein aggregates fraction under *tac* control supplemented with 5% of sucrose. In addition, increased expression was observed in both processed and mature protein.

We implemented osmotic shock as a method for inducing proper protein folding, exposing the bacterial cultures of *E. coli* BL21(DE3)/pGEX-5X-GALNSopt to high concentrations of sucrose. Two concentrations of sucrose were tested for this purpose, 5% and 10% (w/v). As shown in Fig. 3, we observed a statistically significant increase in rhGALNS production when the two conditions were tested, although the highest response was observed using a concentration of 5% (w/v) sucrose. The lower effect observed with 10% (w/v) sucrose, in comparison with 5% (w/v) sucrose, agrees with Barth, *et al*.^32^ report, who suggested that an excessive osmotic stress in the absence of cytoplasmic compatible solutes to rescue cell grow from inhibitory conditions (e.g. organic osmolytes as betaine, carnitine, trehalose, proline, mannitol, and small peptides^33^), could hinder recombinant protein folding.

The largest increments in rhGALNS activity were obtained at the intracellular level, with enzyme activity levels of rhGALNS of 0.06 U/(mg total protein) and 0.14 U/ml (increments of 507% and 219%, respectively, in comparison to an unexposed culture) when cells were grown in presence of 5% (w/v) sucrose. This result is consistent with the effect observed in the Western-blot (Fig. 3E **)**. We did not observe a large increment in the amount of rhGALNS at the intracellular fraction, even if the intracellular activity increased when sucrose was added, suggesting that osmotic stress increases the proper folding of proteins at the cytoplasmic space. However, we estimated a 3-fold increment in rhGALNS recovered from the insoluble fraction, as shown in the Western-blot.

On the other hand, an interesting effect occurred with the protein secreted to the culture medium. As shown in Fig. 3A and B, although a statistically significant increment in specific activity after 24 h of induction was not observed, there was a significant increase in the volumetric activity. The same tendency was evidenced when 10% (w/v) sucrose was used. Elevated amounts of secreted rhGALNS were quantified using an indirect ELISA, as shown in Supplementary Data, where approximately 300% more rhGALNS was found in the medium when the culture was exposed to osmotic stress. In this sense, these results indicates an elevation in the amount of secreted protein but not in its folding, as well as that osmotic stress does not increase the proper folding of proteins at the periplasmic space. This effect can be explained since bacterial cells, under osmotic stress, accumulate small molecules known as osmolytes in the cytoplasm, acting as chemical chaperones^34, 35^.

**Figure 4:**
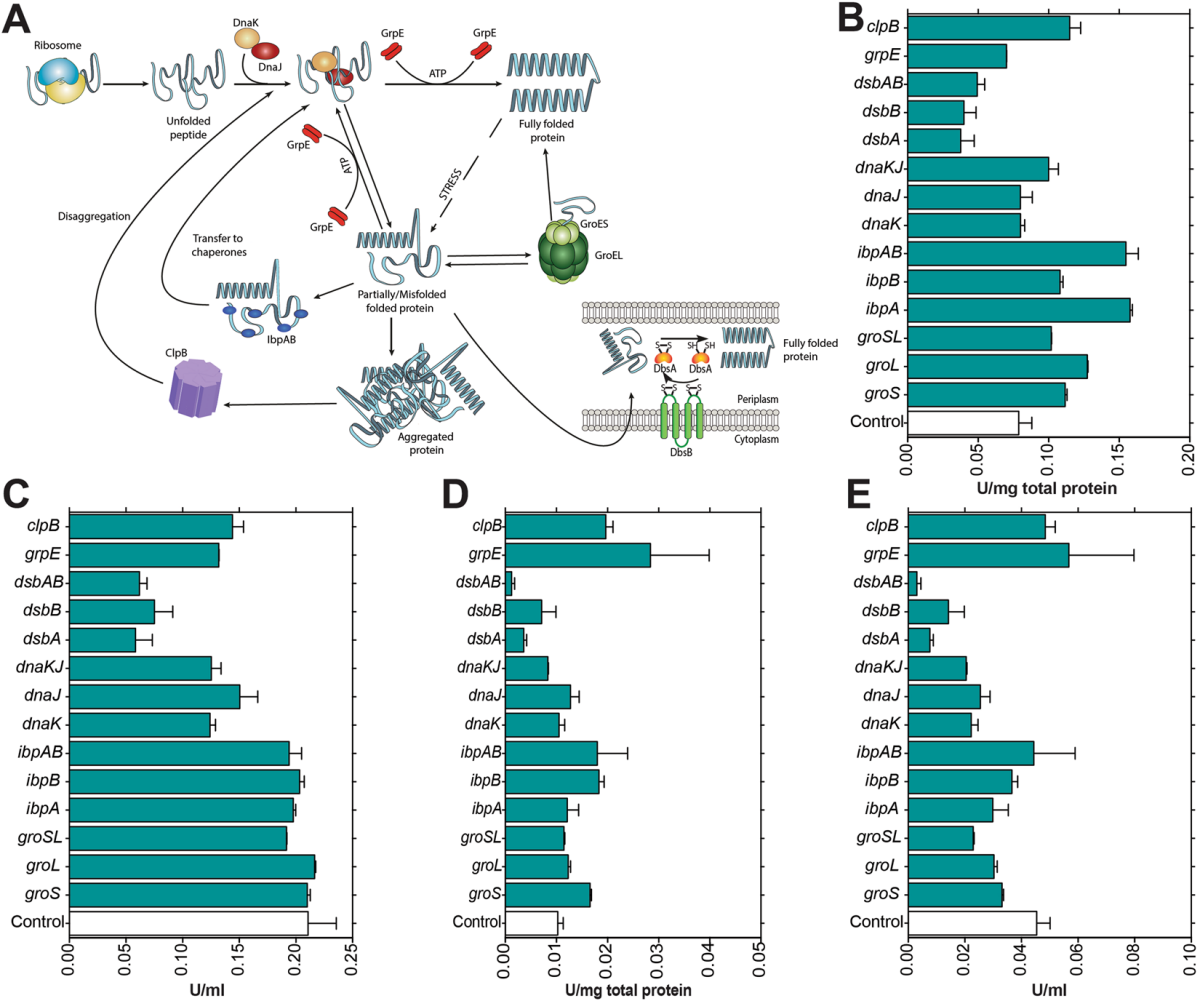
Production of rhGALNS in *E. coli* BL21(DE3) when different chaperones were co-expressed. (**A**) The biological role of the overexpressed chaperones in protein folding in *E. coli*. The scheme shows the importance of the different chaperones in proper folding of nascent peptides, recovery from partially/misfolded proteins and the response to stress. (**B**) Specific activity in the extracellular fraction. (**C**) Volumetric activity in the extracellular fraction. (**D**) Specific activity in the intracellular fraction. (**E**) Volumetric activity in the intracellular fraction. Enzyme activity was assessed 24 hours after induction using optimal (0.5 mM) IPTG concentration. A Student’s t-test was performed to assess significance, where (**) corresponds to a p-value < 0.01. Specific activities are expressed as U rhGALNS/(mg total protein) and volumetric activities as U/ml. As noticed, the overexpression of individual chaperone proteins did not aid in the increase of rhGALNS activity. The names shown in this panels B to E correspond to the overexpressed chaperones. Full names of the plasmids used can be found in Table 1.

Overexpression studies were carried out to determine the effect of individual chaperones genes on the enzyme activity of rhGALNS, or on the contrary, if the observed benefit via osmotic stress was due to a global stress response. Several chaperones were selected for these overexpression studies including the chaperonin system GroESL (genes *groS* and *groL*)^36^; the chaperones DnaK, DnaJ and GrpE capable of repairing heat-inducing protein damage^37^. heat-shock proteins IbpA and IbpB involved in the binding of protein aggregates in *E. coli* ^38^; the complex DsbA-DsbB involved in the formation of disulfide bonds in the periplasmic space^39^ and the chaperone ClpB^40^. The role of these chaperones on protein folding is schematize in Fig. 4A. The plasmids for the overexpression of the chaperones proteins are summarized in Table 1 and Supplementary Data. The *E. coli* strain BL21(DE3) was co-transformed with the plasmids pGEX-5X-GALNSopt and a recombinant pACYCDuet™-1 plasmid for overexpression of the different chaperone genes (Table 1). The clones were screened based on the double antibiotic selection and tested for rhGALNS production in 100 mL as described in the Materials and Methods section. We did not observe a benefit neither in specific nor in volumetric rhGALNS activity when the chaperones were co-expressed alongside rhGALNS (Fig. 4). Even though chaperones are cataloged as folding modulators in the production of recombinant proteins^41, 42^, their co-production has been shown to reduce the yield and quality of several recombinant proteins produced in *E. coli*, as observed in the production of horseradish peroxidase^43^, guinea pig liver transglutaminase^44^, fibroblast growth factor^45^, cyclodextrin glycosyltransferase^46^ and different antibodies fragments^47, 48^. In addition, overexpression of chaperones can contribute to metabolic burden, thereby leading to growth rate reduction as well as decreased final biomass yields^49^. It remains to be determined what genes are involved in the increment of rhGALNS activity once the cells are exposed to high concentrations of sucrose, but we hypothesize based on the findings described above, that this is the effect of a global stress response and not to the action of individual chaperones.

### Improving formation of disulfide bonds

The disulfide bond is the most common link between amino acids after the peptide bond^50^ and around 15% of human produced proteins are predicted to have disulfide bonds^51^. In the instance of the human GALNS, it contains three disulfide bonds per monomer, increasing its stability and activity^52^. The formation of disulfide bonds in all organisms are compartmentalized in the extra-cytoplasmic sections such as the endoplasmic reticulum in eukaryotes, or the periplasmic space of gram-negative bacteria, such as *E. coli* ^53, 54^. This phenomenon is due to the presence of enzymes devoted to the reduction of disulfide bonds in the cytoplasm. In *E. coli*, the oxidative environment in the periplasm allows the formation of disulfide bonds, crucial for the activity and stability of several proteins, promoting resistance against proteases and harsh environments^50^. Therefore, proteins requiring disulfide bonds for their folding and stability, such as the rhGALNS, are prone to be misfolded and not active when expressed in the cytoplasm of *E. coli*. We hypothesized that the lower activity of the intracellular rhGALNS, in comparison to its extracellular counterpart, could be associated with inefficient protein folding due to the reducing cytoplasmic environment. For this reason, we tested the *E. coli* strain SHuffle® T7. This strain is an engineered version of *E. coli* BL21, modified to allow the formation of stable disulfide bonded proteins within the cytoplasm^55^. This modification was generated by the deletion of the thioredoxin reductase (*trxB*) and glutathione reductase (*gor*) genes, the presence of the mutant peroxidase AhpC* to restore reducing power to the bacteria, and the overexpression of the chaperone DsbC lacking the signal sequence to be co-expressed in the cytoplasm^55^.

**Figure 5:**
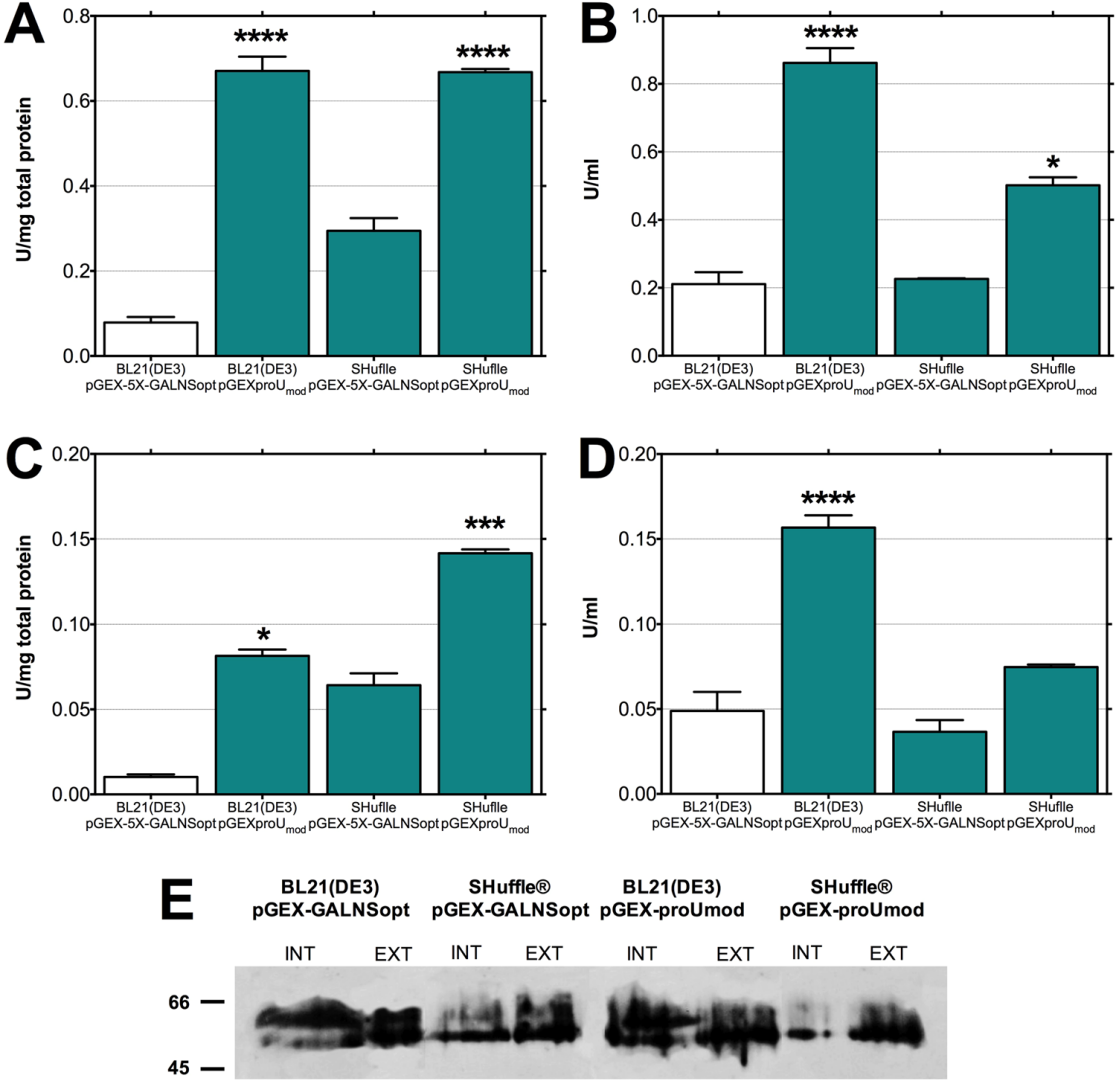
Production of rhGALNS using the promoters *tac* and *proU*_*mod*_, with two different *E. coli* strains: BL21(DE3) and SHuffle® T7. (**A**) Specific activity in the extracellular fraction. (**B**) Volumetric activity in the extracellular fraction. (**C**) Specific activity in the intracellular fraction. (**D**) Volumetric activity in the intracellular fraction. Enzyme activity was assessed 24 hours after induction (no induction was necessary in the case of the promoter *proU*_*mod*_). A Student’s t-test was performed to assess significance, where (*) corresponds to a p-value < 0.05, (***) p-value < 0.001 and (****) p-value < 0.0001. Specific activities are expressed as U rhGALNS/(mg total protein) and volumetric activities as U/ml. (**E**) Western-blot of rhGALNS levels in the intracellular and extracellular fractions in the different studied conditions. All samples were normalized by the amount of total protein. Non-cropped versions of these figures can be found in the Supplementary Data.

We transformed the *E. coli* strain SHuffle® T7 either with the plasmids pGEX-5X-GALNSopt or pGEXproUmod. As observed in Fig. 5A and B, there were not statistically significant differences in the extracellular specific and volumetric activities of rhGALNS in *E. coli* strain SHuffle® T7 when the gene expression was controlled by the *tac* promoter, in comparison to the results observed in *E. coli* BL21(DE3). When the promoter *proU*_*mod*_ was used, the outcome was similar to the previously described in regard of the specific activity, but we observed a reduced volumetric activity of rhGALNS. This effect can be attributed to the lower biomass reached by the strain *E. coli* SHuffle® T7 (1.8 g/L) than the one obtained using *E. coli* BL21(DE3) (3.2 g/L), which could be associated with the numerous genetic modifications in the strain *E. coli* SHuffle® T7. Nevertheless, by using the *proU*_*mod*_ promoter, a large improvement occurred at the cytoplasm with an increase of the specific activity of 1,283% [0.14 U/(mg total protein)] (Fig. 5C and D). We evaluated the increments of protein folding by using a Western-blot (Fig. 5E). The Western-blot analysis indicates a reduction of the intracellular levels of the precursor rhGALNS using *E. coli* SHuffle® T7, in comparison with the levels observed by using *E. coli* BL21(DE3). Through a densitometric analysis of the Western-blot results, we estimated the concentrations of rhGALNS present in the evaluated samples, in order to assess the specific enzyme activity (U rhGALNS/mg rhGALNS), thus determining an approximate increment in enzyme activity of 7X at the intracellular fraction when either plasmids (pGEX-5X-GALNSopt or pGEXproUmod) were used in conjunction to the strain *E. coli* SHuffle® T7. However, we did not observe important variations at the extracellular fraction. This result is in accordance with the expected effect using *E. coli* SHuffle® T7, since all the genetic modifications in this strain are designed to improve intracellular protein folding due to an oxidative cytoplasmic conditions, but not at the periplasmic space where the disulfide forming environment is already present. Similar results were reported in the production of human herpesvirus type-6, with production increments up to 5-fold in comparison to the control strain *E. coli* BL21(DE3)^56^ and the production of the neurosecretory protein GM, with presence of intracellular soluble protein in contrast to the *E. coli* BL21^57^.

### Combinatorial effect

**Figure 6:**
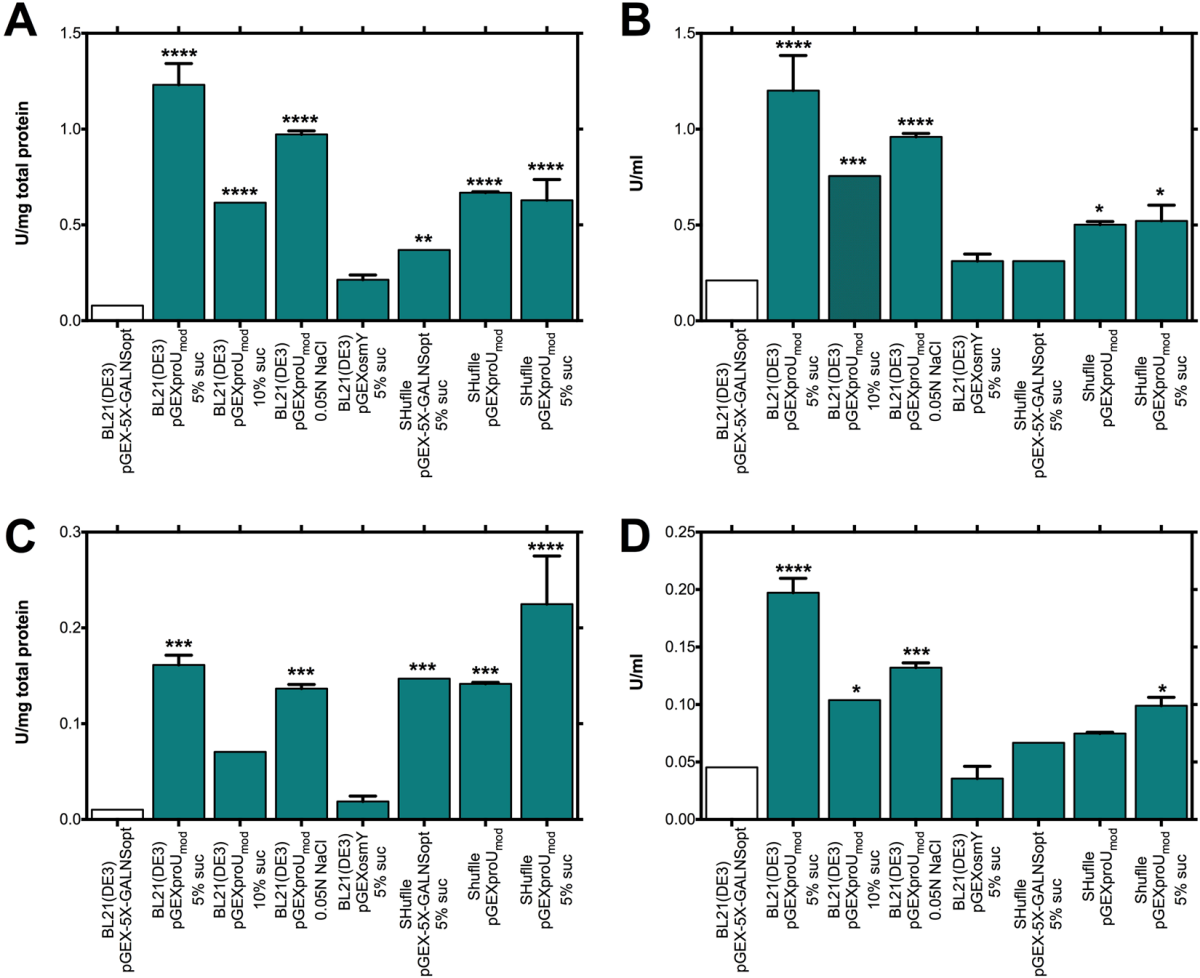
Production of rhGALNS in *E. coli* using the combinations of the different studied approaches. (**A**) Specific activity in the extracellular fraction. (**B**) Volumetric activity in the extracellular fraction. (**C**) Specific activity in the intracellular fraction. (**D**) Volumetric activity in the intracellular fraction. Enzyme activity was assessed 24 hours after induction (no induction was necessary in the case of the promoter *proU*_*mod*_). A Student’s t-test was performed to assess significance, where (*) corresponds to a p-value < 0.05, (**) p-value < 0.01, (***) p-value < 0.001 and (****) p-value < 0.0001. Specific activities are expressed as U rhGALNS/(mg total protein) and volumetric activities as U/ml.

Lastly, we tested the combination of the studied approaches to determine possible additive effects. The largest increment of rhGALNS activity in the extracellular fraction occurred with the use of *proU*_*mod*_ promoter and 5% (w/v) sucrose, obtaining activities of 1.23 U/(mg total protein) and 1.20 U/ml, which represent an improvement of 1,463% and 470%, respectively, in comparison to the results obtained with *E. coli* BL21(DE3)/pGEX-5X-GALNSopt. These substantial improvements also occurred at the intracellular level as seen in Fig. 6 and Table 2, with activities of 0.16 U/(mg total protein) and 0.20 U/ml, which represent an increments of 1,475% and 335%, respectively in comparison to BL21(DE3)/pGEX-5X-GALNSopt.

**Table 2:** Summary of the enzymatic rhGALNS activities obtained in this work after 24 hours of induction.

Studied strains	Extracellular		Intracellular	
	U/mg total protein (% of improvement)	U/ml (% of improvement)	U/mg total protein (% of improvement)	U/ml (% of improvement)
BL21(DE3)/pGEX-5X-GALNSopt	0.08 ± 0.01	0.21 ± 0.04	0.01 ± 0.00	0.05 ± 0.01
BL21(DE3)/pGEX-5X-GALNSopt 5% suc	0.23 ± 0.03 (197%)	**0.67 ± 0.09 (216%)**	**0.06 ± 0.02 (507%)**	**0.14 ± 0.04 (219%)**
BL21(DE3)/pGEX-5X-GALNSopt 10% suc	0.20 ± 0.04 (149%)	**0.45 ± 0.00 (116%)**	0.01 ± 0.00 (0%)	**0.10 ± 0.03 (122%)**
BL21(DE3)/pGEXproUmod	**0.67 ± 0.03 (751%)**	**0.86 ± 0.04 (309%)**	**0.08 ± 0.00 (695%)**	**0.16 ± 0.01 (245%)**
BL21(DE3)/pGEXproUmod 5% suc	**1.23 ± 0.16 (1,463%)**	**1.20 ± 0.26 (470%)**	**0.16 ± 0.01 (1,475%)**	**0.20 ± 0.02 (335%)**
BL21(DE3)/pGEXproUmod 10% suc	**0.62 ± 0.07 (682%)**	**0.76 ± 0.09 (259%)**	**0.07 ± 0.00 (589%)**	**0.10 ± 0.00 (129%)**
BL21(DE3)/pGEXproUmod 0.05 N NaCl	**0.97 ± 0.03 (1,134%)**	**0.96 ± 0.03 (355%)**	**0.14 ± 0.01 (1,234%)**	**0.13 ± 0.01 (191%)**
BL21(DE3)/pGEXosmY	0.20 ± 0.04 (156%)	0.34 ± 0.07 (63%)	0.02 ± 0.00 (52%)	0.05 ± 0.01 (8%)
BL21(DE3)/pGEXosmY 5% suc	0.21 ± 0.04 (171%)	0.31 ± 0.05 (48%)	0.02 ± 0.01 (84%)	0.04 ± 0.02 (−22%)
SHuffle/pGEX-5X-GALNSopt	0.29 ± 0.03 (274%)	0.23 ± 0.00 (7%)	0.06 ± 0.01 (527%)	0.04 ± 0.01 (−19%)
SHuffle/pGEXproUmod	**0.67 ± 0.01 (748%)**	**0.50 ± 0.02 (138%)**	**0.14 ± 0.00 (1,283%)**	0.07 ± 0.00 (65%)
SHuffle/pGEX-5X-GALNSopt 5% suc	**0.37 ± 0.04 (369%)**	0.31 ± 0.06 (48%)	**0.15 ± 0.02 (1,336%)**	0.07 ± 0.01 (47%)
SHuffle/pGEXproUmod 5% suc	**0.63 ± 0.15 (698%)**	**0.52 ± 0.12 (147%)**	**0.22 ± 0.07 (2,095%)**	**0.10 ± 0.01 (118%)**

Values in bold correspond to statistically significant improvements using a t-student test with a p-value < 0.05.

We tested if the use of a different osmotic agent, in a similar osmolarity as implemented with sucrose, leads to the same effect on rhGALNS activities. As seen in Fig. 6, using sodium chloride at 0.05 N, in conjunction with the use of the promoter *proU*_*mod*_, allows the production of similar enzymatic activities to the observed using sucrose in combination to the same promoter. This result suggests that the osmotic effect on rhGALNS activity is not due to the nature of the osmoreagent. Aspedon *et al*., using a transcriptomic analysis in *Pseudomonas aeruginosa* under osmotic stress with sodium chloride and sucrose indicated that stress response to osmotic shock is not dependent of a specific compound or its ionic nature, but it is rather a general stress response process^58^.

Finally, we observed that the highest intracellular specific activity was obtained by combining all the approaches studied in this work (i.e., 5% (w/v) of sucrose, the strain *E. coli* SHuffle® T7 and the promoter *proU*_*mod*_). This improvement corresponds to 2,095% of the activity observed under the initial culture conditions [0.22 U/(mg total protein)]. This arrangement permitted statistically significant improvements of the other studied variables, but it was not as high as with other combinations.

## Conclusions

In summary, several approaches were used to increase the production of the human recombinant enzyme GALNS produced in *E. coli*. These approaches included the control of rhGALNS expression using a promoter regulated under *σ* ^*s*^, the induction of osmoprotectans production through osmotic shock by using sucrose or sodium chloride, the improvement in the formation of disulfide bonds in the cytoplasmic space, and the combination of different angles to explore additive effects. The use of the promoter *proU*_*mod*_ permitted an improvement in protein folding, with estimated increments of rhGALNS specific activities of 20X and 15X in the intracellular and extracellular fractions, respectively. This improvement could be attributed to the reduction of gene expression, as evidenced in Fig. 1E, permitting the protein folding machinery of the bacteria to cope with the folding of the recombinant protein. The nature of *proU*_*mod*_ eliminates the use of inducers, due to the regulation of the promoter by the sigma factor *σ* ^*s*^, thus facilitating the protein production bioprocess. We also utilized 5% (w/v) sucrose to expose bacterial cultures to osmotic shock, favoring a proper protein folding. We found important improvements at the intracellular level (Table 2), possibly due to a global stress response. This hypothesis was supported when several chaperones were co-expressed alongside rhGALNS. In this instance, none of the tested chaperones increased the rhGALNS activity, which agrees with the idea that the increase in the rhGALNS activity after osmotic shock induction was probably due to a general stress response and not to the action of a particular chaperone protein. On the other hand, as an approximation to increase proper folding of the recombinant protein by boosting the formation of disulfide bonds, we used the strain *E. coli* SHuffle® T7 as a host for protein production. We observed important improvements in protein activity in the intracellular fraction when rhGALNS was driven by either the promoter *tac* or *proU*_*mod*_. However, the low biomass yield hinders the use of this strain in the production of large amounts of rhGALNS. Lastly, we reported that high concentrations of sucrose in conjunction with the physiological regulated promoter *proU*_*mod*_ significantly increased the rhGALNS production and activity. Taken together, these results represent valuable information for the production of human lysosomal enzymes in *E. coli*, and could have a significant impact on the development of enzyme replacement therapies for lysosomal storage diseases.

## Materials and Methods

### Bacterial strains and plasmids construction

The *E. coli* strains, BL21(DE3) (*B F- ompT gl dcm lon hsdSB* (*rB* ^−^ *mB* ^−^) λ (DE3[*lacI lacUV5-T7 gene1 ind1 sam7 nin5*]) [*malB* ^+^] K-12 (λS)) and SHuffle® T7 (New England Biolabs, Ipswich, MA, USA) (F- *lac pro laclQ*|Δ(*ara-leu*)*7697 araD139 fhuA2 lacZ::T7 gene1 Δ(phoA)*PvuII phoR ahpC* galE (or U) galK λatt::pNEB3-r1-cDsbC (SpecR, *lacIq*) Δ*trxB rpsL150*(StrR) Δ*gor* Δ(*malF*)3) were used in this study.

Previously, we showed that recombinant GALNS produced in *E. coli* BL21(DE3) had a lower enzyme activity in the crude extract (*i.e*. production) than that reported using CHO cells^9^. In an attempt to increase the enzyme production, GALNS cDNA sequence (GenBank accession number NM_000512.4) was optimized by adapting the codon usage to the bias of *E. coli*, as well as by removing any negative cis-acting sites (*i.e*. splice sites, TATA-boxes, etc.). Optimized GALNS cDNA (GALNSopt) was synthesized by InvitrogenTM GeneArt® (Invitrogen, Carlsbad, CA, USA). The synthetic gene was inserted between the *Eco*RI and *Xho*I sites of pGEX-5X-3 (GE Healthcare, Piscataway, NJ, USA) to generate pGEX-5X-GALNSopt plasmid (6.4 kb) where rhGALNS expression is driven by the *tac* promoter. This plasmid was used as control of rhGALNS expression in most of the experiments carried out in this study.

The pGEX-5X-GALNSopt plasmid was used as backbone for the expression of rhGALNS under the control of the physiologically regulated promoters *proU*_*CCTATAAT*_ (5′-GGG GCC GCC TCA GAT TCT CAG TAT GTT ATA ATA GAA AA-3′)^22^ and *osmY* (5′-TAT CCC GAG CGG TTT CAA AAT TGT GAT CTA TAT TTA ACA AA-3′)^23^. The ribosomal binding sites were designed to maximize the translational initiation rates, using the RBS Calculator from Salis Lab^59^. The RBS designed for the promoters *osmY* and *proU*_mod_ (*proU*_*CCTATAAT*_) were 5′-CGA AAT CAA CAA AAG CGG TTA CTA AC-3′ and 5′-GCG AAC GGA AAT CTA CGG TTA ACA T-3′, respectively. All primers used in this study are summarized in Table 3. For the construction of the plasmid pGEXosmY, the backbone was amplified via PCR with high-fidelity Bestaq™ DNA Polymerase (Applied Biological Materials, Richmond, BC, Canada), using the primers *pGEX-5X-f* and *osmY-r* and the rhGALNS gene was amplified using the primers *osmY-f* and *pGEX-5X-r*. For the generation of these amplicons, the plasmid pGEX-5X-GALNSopt was used as template for PCR. The “scarless” cloning technique, Sequence and Ligation Independent Cloning (SLIC)^60^ was used to create the DNA assembles RBS/promoter/backbone. In short, equimolar amounts of the amplicons (total 200 ng) were mixed with 1 µL of 1/5 diluted (in water) 100X bovine serum albumin (New England Biolabs) and 1 µL 1/5 diluted T4 DNA Polymerase [1 uL NEBuffer2, 7 uL H_2_0, 2 µL T4-Polymerase (3 U/ml) (New England Biolabs)]. The total reaction volume was 20 µL. The mixture was incubated at 22 °C for 5 min, heated up to 70 °C for 20 minutes, followed by 30 minutes at 37 °C using a 10% ramp, and then kept at 4 °C for 18 hours. The reaction product was transformed via electroporation into *E. coli* DH5α and grown onto solid LB agar supplemented with 100 ng/µL of ampicillin. Transformants were screened with restriction endonucleases and the correct construct was transformed in *E. coli* BL21(DE3) using heat shock. A similar protocol was implemented for the construction of the plasmid pGEXproUmod, where the primers *pGEX-5X-f* and *proU-r*, and *proU-f* and *pGEX-5X-r* were used to obtain the amplicons corresponding to the backbone and rhGALNS, assembled together using SLIC as previously described.

**Table 3:** Primers used in this study.

Name	Sequence
*pGEX-5X-f*	5′-CCT ATT TTT ATA GGT TAA TGT CAT GAT AAT AAT GGT TTC TTA GAC GTC-3′
*pGEX-5X-r*	5′-CAT TAA CCT ATA AAA ATA GGC GTA TCA CGA GGC CCT TTC -3′
*osmY-f*	5′-TCT ATA TTT AAC AAA CGA AAT CAA CAA AAG CGG TTA CTA ACA TGT CCC CTA TAC TAG GTT ATT G-3′
*osmY-r*	5′-TTT CGT TTG TTA AAT ATA GAT CAC AAT TTT GAA ACC GCT CGG GAT AGG AAT TTA TGC GGT GTG AAA TAC -3′
*proU-f*	5′-ATT CTC AGT ATG TTA TAA TAG AAA AGC GAA CGG AAA TCT ACG GTT AAC ATA TGT CCC CTA TAC TAG GTT ATT G-3′
*proU-r*	5′-ATT TCC GTT CGC TTT TCT ATT ATA ACA TAC TGA GAA TCT GAG GCG GCC CCG GAA TTT ATG CGG TGT GAA ATA C-3′
*groS-f*	5′-ATC CGA ATT CAT GAA TAT TCG TCC ATT GCA TGA T-3′
*groS-r*	5′-CGC AAG CTT TTA CGC TTC AAC AAT TGC CAG-3′
*groL-f*	5′-TAT ACA TAT GAT GGC AGC TAA AGA CGT AA-3′
*groL-r*	5′-CAG ACT CGA GTT ACA TCA TGC CGC CCA TG-3′
*dnaK-f*	5′-ATT CGA GCT CAT GGG TAA AAT AAT TGG TAT CGA C-3′
*dnaK-r*	5′-CCG CAA GCT TTT ATT TTT TGT CTT TGA CTT CTT CAA ATT C-3′
*dnaJ-f*	5′-TAT ACA TAT GAT GGC TAA GCA AGA TTA TTA CGA G-3′
*dnaJ-r*	5′-CAG ACT CGA GTT AGC GGG TCA GGT CGT C-3′
*dsbA-f*	5′-ATC CGA ATT CAT GAA AAA GAT TTG GCT GGC G-3′
*dsbA-r*	5′-CCG CAA GCT TTT ATT TTT TCT CGG ACA GAT ATT TCA CT-3′
*dsbB-f*	5′-TAT ACA TAT GAT GTT GCG ATT TTT GAA CCA ATG-3′
*dsbB-r*	5′-CAG ACT CGA GTT AGC GAC CGA ACA GAT CAC G-3′
*ibpA-f*	5′-ATC CGA ATT CAT GCG TAA CTT TGA TTT ATC CCC-3′
*ibpA-r*	5′-CCG CAA GCT TTT AGT TGA TTT CGA TAC GGC GC-3′
*ibpB-f*	5′-TAT ACA TAT GAT GCG TAA CTT CGA TTT ATC CCC-3′
*ibpB-r*	5′-AGA CTC GAG TTA GCT ATT TAA CGC GGG ACG T-3′
*grpE-f*	5′-ATC CGA ATT CAT GAG TAG TAA AGA ACA GAA AAC G-3′
*grpE-r*	5′-ATG CGG CCG CTT AAG CTT TTG CTT TCG CTA CAG-3′
*clpB-f*	5′-TAT ACA TAT GAT GCG TCT GGA TCG TCT TAC-3′
*clpB-r*	5′-CAG ACT CGA GTT ACT GGA CGG CGA CAA TC-3′
*GALNS -f*	5′-GTG AAC CGA GCC GTG AAA CC-3′
*GALNS-r*	5′-ACC AAT GCA CAT GCA CGC AA-3′

All the corresponding genes for the chaperones in study were amplified via PCR from genomic DNA of *E. coli* BL21(DE3) using the high-fidelity Bestaq™ DNA Polymerase (Applied Biological Materials) and cloned into the bicistronic plasmid pACYCDuet™-1 (Novagen, Merck Millipore, Billerica, MA, USA) by restriction digest. All the constructs are summarized in Table 1 and their respective maps in Supplementary Data. For the co-expression of the chaperone protein and rhGALNS, the corresponding plasmid was transformed using heat-shock into the strain *E. coli* BL21(DE3)/pGEX-5X-GALNSopt and screened using double antibiotic selection (AmpR + CmR).

### Culture conditions

Overnight cultures were grown in LB liquid medium or on solid LB agar plates supplemented with appropriated antibiotics and incubated at 37 °C. Production of rhGALNS was carried out in 100 mL cultures using M9 minimum medium [2 mM MgSO_4_, 0.1 mM CaCl_2_, 6.78 g/L Na_2_HPO_4_, 3 g/L KH_2_PO, 4.5 g/L NaCl, 1 g/L NH_4_Cl, 1 mg/L thiamine and trace elements. 1000X trace elements solution: 13.4 mM ethylenediamine tetraacetic acid (EDTA), 3.1 mM FeCl_3_.6H_2_O, 0.62 mM ZnCl_2_, 76 µM CuCl_2_.2H_2_O, 42 µM CoCl_2_.2H_2_O, 162 µM H_3_BO_3_, 8.1 µM MnCl_2_.4H_2_O, 36.3 mg/L AlCl, 2.9 mg/L NiCl_2_·6H_2_O] supplemented with 20 g/L of D-glucose under aerobic conditions, using appropriated antibiotics. When necessary, gene expression was induced using isopropyl β-D-1-thiogalactopyranoside (IPTG, Gold Biotechnology, St. Louis, MO, USA) at 0.5 mM after 8 hours of incubation. All cultures were maintained at 180 RPM during cultivation. Cells were cultivated for 24 hours after induction. Biomass was estimated using optical density at 540 nm. To evaluate the effect of osmotic stress on protein production and activity, sucrose was added to the cultures at 5% and 10% (w/v) or sodium chloride to a concentration of 0.5 mM. All assays were done in triplicate.

### Crude protein assays

Fifty mL cultures were centrifuged and the growth media was saved for further analysis. The pellets were resuspended in 5 mL lysis buffer (25 mM Tris, 1 mM EDTA, 1 mM phenylmethylsulfonyl fluoride, 5% (v/v) glycerol, and 1% (v/v) Triton X-100, to a pH 7.2). Samples were sonicated during 1 minute at 4 °C and 25% amplitude (Vibra-Cell, Sonics & Materials Inc., Newtown, CT, USA) and centrifuged at 3000 × g and 4 °C during 20 minutes^9, 61^. The soluble fraction (supernatant) was stored at −20 °C for further analysis. Recovered pellets were processed for analysis of the insoluble fraction. The protein aggregates were solubilized by mixing 2 µL of lysed cells in 30 µL of 6X SDS loading buffer (375 mM Tris-HCl pH 6.8, 6% sodium dodecyl sulfate (SDS), 48% glycerol, 9% 2-mercaptoethanol, and 0.03% bromophenol blue) and boiled at 95 °C for 10 min.

### Quantification of GALNS activity

rhGALNS activity was assessed using the fluorescent substrate 4-methylumbelliferyl-β-D-galactopyranoside-6-sulfate (Toronto Chemicals Research, North York, ON, Canada) as described elsewhere^62^. One unit (U) was defined as the amount of rhGALNS catalyzing 1 nmol substrate per hour, and rhGALNS activity was expressed as U/(mg total protein) (total protein determined by Lowry protein assay^63^) or as U/mL. Enzyme activity was assayed in the soluble fraction (intracellular) and growth media (extracellular).

### SDS–PAGE Analysis

Crude proteins extracts and growth media were analyzed by SDS - polyacrylamide gel electrophoresis (SDS-PAGE) under reducing conditions as described by Laemmli *et al*.^64^. SDS-PAGE was carried out using 12% (w/v) polyacrylamide gels and 3% (w/v) polyacrylamide stacking gel. The molecular masses were estimated using the protein ladder Precision Plus Protein™ (BioRad, Hercules, CA, USA). The gels were stained with Coomassie blue R-250 (BioRad).

### Western blotting

The proteins from different fractions were homogenized in lysis buffer and equivalent amounts of total protein extracts were loaded and processed by SDS-PAGE gels and electroblotted onto nitrocellulose membranes (Amershan Protan^TM^ 0.45 µm, GE Healthcare, Little Chalfont, UK). The membranes were blocked with a blocking buffer containing TTBS [0.3% Tween 20, and 5% BSA (Sigma-Aldrich, St. Louis, MO, USA)] at room temperature for 1 hour. Membranes containing resuspended protein aggregates, extracellular and intracellular proteins were incubated overnight at 4 °C with a specific primary antibody rabbit anti human GALNS (1:1000 in blocking buffer)^9^. The specificity of this antibody was previously assessed, and no band was recognized in samples from non-induced and plasmid-free strains^9^. A peroxidase conjugated goat anti-rabbit (Sigma-Aldrich) was added (1:2000 in blocking buffer) for 1 hour at room temperature. The specific bands were visualized using enhanced chemiluminescence (SuperSignal™ West Pico Chemiluminescent Substrate, Thermo Fisher Scientific®, Waltham, MA, USA).

### qRT-PCR experiments

Frozen stocks of the strain BL21(DE3)/pGEXproU_mod_ (0.5 mL) were transferred into 10 mL of fresh LB media supplemented with 100 ng/mL of ampicillin and incubated for 18 h at 37 °C and 180 rpm. Production of rhGALNS was carried out in 100 mL cultures using M9 minimum medium supplemented with 20 g/L of D-glucose under aerobic conditions, inoculated with 10 mL of overnight culture, kept at 37 °C and 180 rpm. Samples (10 mL) were taken 2, 4, 7, 12 and 24 hours after inoculation of all three biological replicas. The samples were pelleted by centrifugation at 3000 x g and 4 °C during 10 minutes. The supernatant was discarded and the pellets were immediately placed at −80 °C until further processing. The samples were processed in a period lower than 12 hours after pelleting. Total RNA was extracted using the ZR Fungal/Bacterial RNA Miniprep^TM^ Kit (Zymo Research, Irvine, CA, USA), following manufacturer’s indications and immediately stored at −80 °C. The cDNA was generated using the RevertAid First Strand cDNA Synthesis Kit (Thermo Fisher Scientific) using 100 ng of total RNA and following the random hexamer primed synthesis manufacturer’s protocol. Two primers (*GALNS-f* and *GALNS-r*) (Table 3)) were designed for the quantification of rhGALNS transcripts using NCBI Primer Design Tool and they were used for the qPCR experiments. Primer amplification efficiency was assessed using the plasmid pGEXproUmod (purified using the Wizard SV Gel and PCR Clean-up System (Promega, Madison, WI, USA)) as template. The amplification efficiency of these primers was estimated as 94%. To generate a standard curve for absolute rhGALNS copy number quantification, the plasmid pGEXproUmod was serially diluted to obtain different plasmid copies ranging from 300,000 to 30. Triplicates of each data-point were used to ensure reliability of the data. All qPCR experiments were carried out using Luna® Universal qPCR Master Mix (New England Biolabs) following manufacturer’s indications and read in a QuantStudio 3 Real-Time PCR System (Thermo Fisher Scientific). The results as reported as rhGALNS copy number per ng of total RNA.

### Statistical analysis

Statistical significance was evaluated using a Student’s t-test, employing the software GraphPad Prism version 6 for Windows (GraphPad Software, San Diego, California, USA).

## Electronic supplementary material

Supplementary Information

## References

[1] Montano AM, Tomatsu S, Gottesman GS, Smith M, Orii T (2007). International Morquio A Registry: clinical manifestation and natural course of Morquio A disease. J Inherit Metab Dis.

[2] Tomatsu, S. *et al*. Mucopolysaccharidosis type IVA (Morquio A disease): clinical review and current treatment. *Curr Pharm Biotechnol* **12**, 931–945, doi:1389-2010/11 $58.00+0.00 (2011). 10.2174/138920111795542615 21506915

[3] Tomatsu S (2014). Morquio A syndrome: diagnosis and current and future therapies. Pediatr Endocrinol Rev.

[4] Hendriksz CJ (2014). Efficacy and safety of enzyme replacement therapy with BMN 110 (elosulfase alfa) for Morquio A syndrome (mucopolysaccharidosis IVA): a phase 3 randomised placebo-controlled study. J Inherit Metab Dis.

[5] Tomatsu S (2015). Enzyme replacement therapy for treating mucopolysaccharidosis type IVA (Morquio A syndrome): effect and limitations. Expert Opinion on Orphan Drugs.

[6] Tomatsu S (2010). Enhancement of drug delivery: enzyme-replacement therapy for murine Morquio A syndrome. Mol Ther.

[7] Espejo-Mojica Á (2015). Human recombinant lysosomal enzymes produced in microorganisms. Mol Genet Metab.

[8] Bielicki J (1995). Expression, purification and characterization of recombinant human N-acetylgalactosamine-6-sulphatase. Biochem J.

[9] Rodriguez A (2010). Enzyme replacement therapy for Morquio A: an active recombinant N-acetylgalactosamine-6-sulfate sulfatase produced in *Escherichia coli* BL21. J Ind Microbiol Biotechnol.

[10] Rodríguez-López, A. *et al*. Recombinant human N-acetylgalactosamine-6-sulfate sulfatase (GALNS) produced in the methylotrophic yeast *Pichia pastoris*. *Sci Rep* **6**, 29329, doi:10.1038/srep29329http://www.nature.com/articles/srep29329-supplementary-information (2016). 10.1038/srep29329 PMC4932491 27378276

[11] Kamionka M (2011). Engineering of therapeutic proteins production in *Escherichia coli*. Curr Pharm Biotechnol.

[12] Critchley RJ (2004). Potential therapeutic applications of recombinant, invasive *E. coli*. Gene Ther.

[13] Baeshen MN (2015). Production of Biopharmaceuticals in *E. coli*: Current Scenario and Future Perspectives. J Microbiol Biotechnol.

[14] Baneyx F, Mujacic M (2004). Recombinant protein folding and misfolding in *Escherichia coli*. Nat Biotechnol.

[15] Mosquera A (2012). Characterization of a recombinant N-acetylgalactosamine-6-sulfate sulfatase produced in *E. coli* for enzyme replacement therapy of Morquio A disease. Process Biochem..

[16] Hernandez A (2013). Effect of culture conditions and signal peptide on production of human recombinant N-acetylgalactosamine-6-sulfate sulfatase in *Escherichia coli* BL21. J Microbiol Biotechnol.

[17] Carrio MM, Villaverde A (2002). Construction and deconstruction of bacterial inclusion bodies. J Biotechnol.

[18] Muller-Hill B, Crapo L, Gilbert W (1968). Mutants that make more lac repressor. Proc Natl Acad Sci USA.

[19] Sweet CR (2003). Expression of recombinant proteins from *lac* promoters. Methods Mol Biol.

[20] Hannig G, Makrides SC (1998). Strategies for optimizing heterologous protein expression in *Escherichia coli*. Trends Biotechnol.

[21] de Boer HA, Comstock LJ, Vasser M (1983). The *tac* promoter: a functional hybrid derived from the *trp* and *lac* promoters. Proc Natl Acad Sci USA.

[22] Wise A, Brems R, Ramakrishnan V, Villarejo M (1996). Sequences in the −35 region of *Escherichia coli* rpoS-dependent genes promote transcription by E sigma S. J Bacteriol.

[23] Yim HH, Brems RL, Villarejo M (1994). Molecular characterization of the promoter of *osmY*, an *rpoS*-dependent gene. J Bacteriol.

[24] Schneider CA, Rasband WS, Eliceiri KW (2012). NIH Image to ImageJ: 25 years of image analysis. Nat Methods.

[25] Miksch G (2006). A rapid reporter system using GFP as a reporter protein for identification and screening of synthetic stationary-phase promoters in *Escherichia coli*. Applied Microbiology and Biotechnology.

[26] Mergulhao FJ, Taipa MA, Cabral JM, Monteiro GA (2004). Evaluation of bottlenecks in proinsulin secretion by *Escherichia coli*. J Biotechnol.

[27] Rosenberg, H. F. Isolation of recombinant secretory proteins by limited induction and quantitative harvest. *Biotechniques* **24**, 188–190, 192 (1998). 10.2144/98242bm03 9494711

[28] Mergulhao FJ, Summers DK, Monteiro GA (2005). Recombinant protein secretion in *Escherichia coli*. Biotechnol Adv.

[29] Kempf B, Bremer E (1998). Uptake and synthesis of compatible solutes as microbial stress responses to high-osmolality environments. Arch Microbiol.

[30] Chen J, Acton TB, Basu SK, Montelione GT, Inouye M (2002). Enhancement of the solubility of proteins overexpressed in *Escherichia coli* by heat shock. J Mol Microbiol Biotechnol.

[31] de Marco A, Vigh L, Diamant S, Goloubinoff P (2005). Native folding of aggregation-prone recombinant proteins in *Escherichia coli* by osmolytes, plasmid- or benzyl alcohol-overexpressed molecular chaperones. Cell Stress Chaperones.

[32] Barth S (2000). Compatible-solute-supported periplasmic expression of functional recombinant proteins under stress conditions. Appl Environ Microbiol.

[33] Csonka LN (1989). Physiological and genetic responses of bacteria to osmotic stress. Microbiological Reviews.

[34] de Marco, A. In *Protein Aggregation in Bacteria* 77–92 (John Wiley & Sons, Inc., 2014).

[35] Kim S-H, Yan Y-B, Zhou H-M (2006). Role of osmolytes as chemical chaperones during the refolding of aminoacylase. Biochemistry and Cell Biology.

[36] Martin J (1998). Protein folding assisted by the GroEL/GroES chaperonin system. Biochemistry (Mosc).

[37] Schroder H, Langer T, Hartl FU, Bukau B (1993). DnaK, DnaJ and GrpE form a cellular chaperone machinery capable of repairing heat-induced protein damage. EMBO J.

[38] Laskowska E, Wawrzynow A, Taylor A (1996). IbpA and IbpB, the new heat-shock proteins, bind to endogenous *Escherichia coli* proteins aggregated intracellularly by heat shock. Biochimie.

[39] Whitley P, von Heijne G (1993). The DsbA-DsbB system affects the formation of disulfide bonds in periplasmic but not in intramembraneous protein domains. FEBS Lett.

[40] Barnett ME, Zolkiewska A, Zolkiewski M (2000). Structure and activity of ClpB from *Escherichia coli*. Role of the amino-and -carboxyl-terminal domains. J Biol Chem.

[41] de Marco A (2007). Protocol for preparing proteins with improved solubility by co-expressing with molecular chaperones in *Escherichia coli*. Nat Protoc.

[42] de Marco A, De Marco V (2004). Bacteria co-transformed with recombinant proteins and chaperones cloned in independent plasmids are suitable for expression tuning. J Biotechnol.

[43] Kondo A (2000). Improvement of productivity of active horseradish peroxidase in *Escherichia coli* by coexpression of Dsb proteins. J Biosci Bioeng.

[44] Ikura K (2002). Co-overexpression of folding modulators improves the solubility of the recombinant guinea pig liver transglutaminase expressed in *Escherichia coli*. Prep Biochem Biotechnol.

[45] Rinas U, Hoffmann F, Betiku E, Estape D, Marten S (2007). Inclusion body anatomy and functioning of chaperone-mediated *in vivo* inclusion body disassembly during high-level recombinant protein production in *Escherichia coli*. J Biotechnol.

[46] Kim SG, Kweon DH, Lee DH, Park YC, Seo JH (2005). Coexpression of folding accessory proteins for production of active cyclodextrin glycosyltransferase of *Bacillus macerans* in recombinant *Escherichia coli*. Protein Expr Purif.

[47] Hu X (2007). Optimisation of production of a domoic acid-binding scFv antibody fragment in *Escherichia coli* using molecular chaperones and functional immobilisation on a mesoporous silicate support. Protein Expression and Purification.

[48] Levy R, Weiss R, Chen G, Iverson BL, Georgiou G (2001). Production of correctly folded Fab antibody fragment in the cytoplasm of *Escherichia coli* trxB gor mutants via the coexpression of molecular chaperones. Protein Expr Purif.

[49] Berges H, Joseph-Liauzun E, Fayet O (1996). Combined effects of the signal sequence and the major chaperone proteins on the export of human cytokines in *Escherichia coli*. Appl Environ Microbiol.

[50] Fass D (2012). Disulfide bonding in protein biophysics. Annu Rev Biophys.

[51] Wong JW, Ho SY, Hogg PJ (2011). Disulfide bond acquisition through eukaryotic protein evolution. Mol Biol Evol.

[52] Rivera-Colon Y, Schutsky EK, Kita AZ, Garman SC (2012). The structure of human GALNS reveals the molecular basis for mucopolysaccharidosis IV A. J Mol Biol.

[53] Bardwell JC, McGovern K, Beckwith J (1991). Identification of a protein required for disulfide bond formation *in vivo*. Cell.

[54] Weissman JS, Kim PS (1993). Efficient catalysis of disulphide bond rearrangements by protein disulphide isomerase. Nature.

[55] Lobstein J (2012). SHuffle, a novel *Escherichia coli* protein expression strain capable of correctly folding disulfide bonded proteins in its cytoplasm. Microb Cell Fact.

[56] Tait AR, Straus SK (2011). Overexpression and purification of U24 from human herpesvirus type-6 in *E*. *coli*: unconventional use of oxidizing environments with a maltose binding protein-hexahistine dual tag to enhance membrane protein yield. Microbial Cell Factories.

[57] Masuda K, Furumitsu M, Taniuchi S, Iwakoshi-Ukena E, Ukena K (2015). Production and characterization of neurosecretory protein GM using *Escherichia coli* and Chinese Hamster Ovary cells. FEBS Open Bio.

[58] Aspedon A, Palmer K, Whiteley M (2006). Microarray Analysis of the Osmotic Stress Response in *Pseudomonas aeruginosa*. Journal of Bacteriology.

[59] Espah Borujeni A, Channarasappa AS, Salis HM (2014). Translation rate is controlled by coupled trade-offs between site accessibility, selective RNA unfolding and sliding at upstream standby sites. Nucleic Acids Res.

[60] Li MZ, Elledge SJ (2007). Harnessing homologous recombination *in vitro* to generate recombinant DNA via SLIC. Nat Methods.

[61] Thomas JG, Baneyx F (1996). Protein misfolding and inclusion body formation in recombinant *Escherichia coli* cells overexpressing Heat-shock proteins. J Biol Chem.

[62] van Diggelen OP (1990). A fluorimetric enzyme assay for the diagnosis of Morquio disease type A (MPS IV A). Clin Chim Acta.

[63] Dawson JM, Heatlie PL (1984). Lowry method of protein quantification: evidence for photosensitivity. Anal Biochem.

[64] Laemmli UK (1970). Cleavage of Structural Proteins during the Assembly of the Head of Bacteriophage T4. Nature.

