# Supplementary material for: Improvement in the production of the human recombinant enzyme N-acetylgalactosamine-6-sulfatase (rhGALNS) in Escherichia coli using synthetic biology approaches
Source: Sci Rep. 2017 Jul 19;7:5844. doi: 10.1038/s41598-017-06367-w (PMC5517531; doi:10.1038/s41598-017-06367-w)

SUPPLEMENTARY DATA

Improvement in the production of the human recombinant enzyme N-acetylgalactosamine-6-sulfatase (rhGALNS) in *Escherichia coli* using synthetic biology approaches

Luis H. Reyes[1,*], Carolina Cardona[1], Luisa Pimentel[1], Alexander Rodríguez-López[1,2], Carlos J. Alméciga-Díaz[1,*].

[1] Institute for the Study of Inborn Errors of Metabolism, Faculty of Science, Pontificia Universidad Javeriana, Bogotá, Colombia

[2] Chemistry Department, Faculty of Science, Pontificia Universidad Javeriana, Bogotá, Colombia

* Corresponding author

Luis H. Reyes. Protein Expression and Purification Laboratory, Institute for the Study of Inborn Errors of Metabolism, Faculty of Science, Pontificia Universidad Javeriana, Cra 7 No 43 E 82, Building 54, Room 303A, Bogotá, Colombia. Tel.: +57 1 3208320 Ext. 4099. luishreyesbarrios@gmail.com

Carlos Javier Alméciga-Díaz. Institute for the Study of Inborn Errors of Metabolism, Faculty of Science, Pontificia Universidad Javeriana, Cra 7 No 43 E 82, Building 54, Room 303A, Bogotá, Colombia. Tel.: +57 1 3208320 Ext. 4125. cjalmeciga@javeriana.edu.co

1. Contents

2. Plasmid maps used in this study ... 3
a. pGEX-5X-GALNSopt ... 3
b. pGEXosmY ... 7
c. pGEXproUmod ... 10
d. pACYCDuet™-1 ... 13
e. pDuet::GroS ... 16
f. pDuet::GroL ... 19
g. pDuet::GroSL ... 23
h. pDuet::DnaK ... 27
i. pDuet::DnaJ ... 31
j. pDuet::DnaKJ ... 34
k. pDuet::IbpA ... 38
l. pDuet::IbpB ... 41
m. pDuet::IbpAB ... 44
n. pDuet::DsbA ... 47
o. pDuet::DsbB ... 50
p. pDuet::DsbAB ... 53
q. pDuet::GrpE ... 56
r. pDuet::ClpB ... 59
3. Non-cropped Western Blots ... 63
a. Production of rhGALNS in *E. coli* BL21(DE3) using different promoters ... 63
b. Dynamics of the expression of rhGALNS under the control of the promoter *proU*$_{mod}$. ... 64
c. Production of rhGALNS using the promoters *tac* and *proU*$_{mod}$, with two different *E. coli* strains: BL21(DE3) and SHuffle® ... 65
4. Purification via affinity chromatography ... 66
5. Indirect Enzyme-Linked Immunosorbent Assay (ELISA) ... 67

2. Plasmid maps used in this study

a. pGEX-5X-GALNSopt

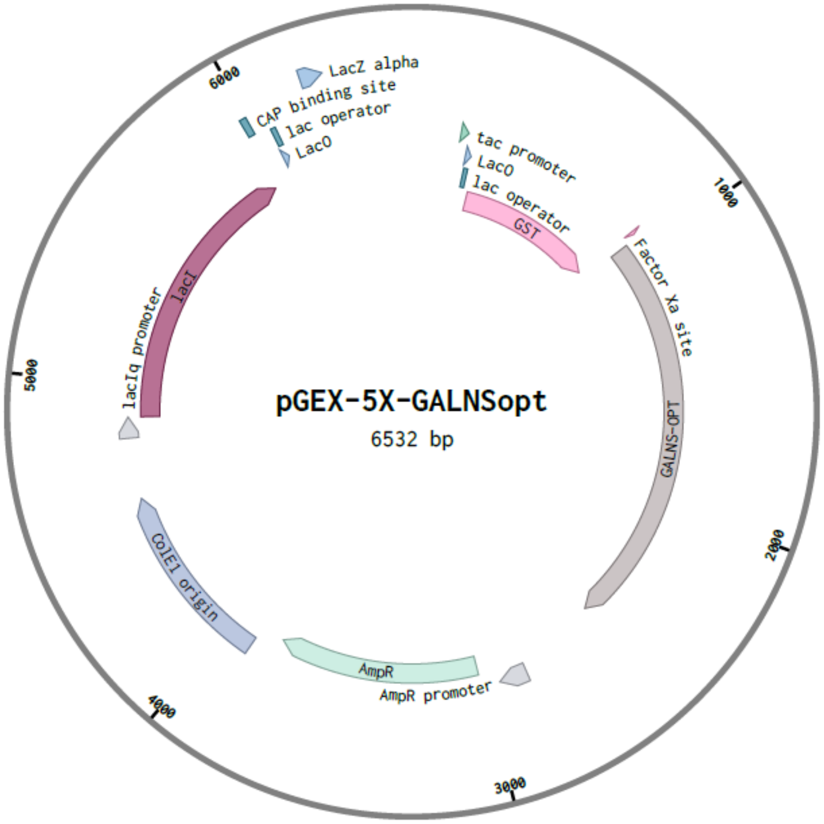

AGCTTATCGACTGCACGGTGCACCAATGCTTCTGGCGTCAGGCAGCCATCGGAAGCTGTGGT
ATGGCTGTGCAGGTCGTAAATCACTGCATAATTCGTGTCGCTCAAGGCGCACTCCCGTTCTGG
ATAATGTTTTTTGCGCCGACATCATAACGGTTCTGGCAAATATTCTGAAATGAGCTGTTGACA
ATTAATCATCGGCTCGTATAATGTGTGGAATTGTGAGCGGATAACAATTTCACACAGGAAAC
AGTATTCATGTCCCCTATACTAGGTTATTGGAAAATTAAGGGCCTTGTGCAACCCACTCGACT
TCTTTTGGAATATCTTGAAGAAAAATATGAAGAGCATTTGTATGAGCGCGATGAAGGTGATA
AATGGCGAAACAAAAAGTTTGAATTGGGTTTGGAGTTTCCCAATCTTCCTTATTATATTGATG
GTGATGTTAAATTAACACAGTCTATGGCCATCATACGTTATATAGCTGACAAGCACAACATG
TTGGGTGGTTGTCCAAAAGAGCGTGCAGAGATTTCAATGCTTGAAGGAGCGGTTTTGGATAT
TAGATACGGTGTTTCGAGAATTGCATATAGTAAAGACTTTGAAACTCTCAAAGTTGATTTTCT
TAGCAAGCTACCTGAAATGCTGAAAATGTTCGAAGATCGTTTATGTCATAAAACATATTTAA
ATGGTGATCATGTAACCCATCCTGACTTCATGTTGTATGACGCTCTTGATGTTGTTTTATACAT
GGACCCAATGTGCCTGGATGCGTTCCCAAAATTAGTTTGTTTTAAAAAACGTATTGAAGCTAT
CCCACAAATTGATAAGTACTTGAAATCCAGCAAGTATATAGCATGGCCTTTGCAGGGCTGGC
AAGCCACGTTTGGTGGTGGCGACCATCCTCCAAAATCGGATCTGATCGAAGGTCGTGGGATC

CCCGAATTCATGGCAGCAGTTGTTGCAGCAACCCGTTGGTGGCAGCTGCTGCTGGTTCTGAG
CGCAGCAGGTATGGGTGCAAGTGGTGCACCGCAGCCTCCGAATATTCTGCTGCTGCTGATGG
ATGATATGGGTTGGGGTGATCTGGGTGTTTATGGTGAACCGAGCCGTGAAACCCCGAATCTG
GATCGTATGGCAGCAGAAGGTCTGCTGTTTCCGAACTTTTATAGCGCAAATCCGCTGAGCAG
CCCGAGCCGTGCAGCACTGCTGACAGGTCGTCTGCCGATTCGTAATGGTTTTTATACCACCAA
TGCACATGCACGCAATGCATATACACCGCAAGAAATTGTTGGTGGTATTCCGGATAGCGAAC
AGCTGCTGCCGGAACTGCTGAAAAAAGCAGGTTATGTTAGCAAAATTGTGGGCAAATGGCAT
CTGGGTCATCGTCCGCAGTTTCATCCGCTGAAACATGGTTTTGATGAATGGTTTGGTAGCCCG
AATTGTCATTTTGGTCCGTATGATAATAAAGCCCGTCCGAATATTCCGGTTTATCGTGATTGG
GAAATGGTGGGTCGTTATTATGAAGAATTTCCGATCAATCTGAAAACCGGTGAAGCAAATCT
GACCCAGATTTATCTGCAAGAAGCACTGGATTTCATTAAACGTCAGGCACGTCATCATCCGTT
TTTTCTGTATTGGGCAGTTGATGCAACCCATGCACCGGTTTATGCAAGCAAACCGTTTCTGGG
CACCAGCCAGCGTGGTCGTTATGGTGATGCAGTTCGTGAAATTGATGATAGCATTGGCAAAA
TTCTGGAACTGCTGCAGGATCTGCATGTTGCAGATAATACCTTTGTGTTTTTCACCAGCGATA
ATGGTGCAGCACTGATTAGCGCACCGGAACAGGGTGGTAGCAATGGTCCGTTTCTGTGTGGT
AAACAGACCACCTTTGAAGGTGGTATGCGTGAACCGGCACTGGCATGGTGGCCTGGTCATGT
TACCGCAGGTCAGGTTAGCCATCAGCTGGGTAGCATTATGGACCTGTTTACCACCAGTCTGG
CACTGGCAGGTCTGACCCCTCCGAGCGATCGTGCAATTGATGGTCTGAATCTGCTGCCGACA
CTGCTGCAGGGACGTCTGATGGATCGTCCGATTTTCTATTATCGTGGTGATACCCTGATGGCA
GCAACCCTGGGTCAGCATAAAGCACATTTTTGGACCTGGACCAATAGCTGGGAAAATTTTCG
TCAGGGCATTGATTTTTGTCCGGGTCAGAATGTTAGCGGTGTTACCACCCATAATCTGGAAGA
TCATACCAAACTGCCGCTGATTTTTCATCTGGGTCGTGATCCGGGTGAACGTTTTCCGCTGAG
CTTTGCAAGCGCAGAATATCAAGAAGCCCTGAGCCGTATTACCAGCGTTGTTCAGCAGCATC
AAGAAGCGCTGGTTCCGGCACAGCCGCAGCTGAATGTTTGTAATTGGGCAGTTATGAATTGG
GCACCGCCTGGTTGTGAAAAACTGGGTAAATGTCTGACCCCACCGGAAAGCATTCCGAAAAA
ATGTCTGTGGTCACATTAACTCGAGCGGCCGCATCGTGACTGACTGACGATCTGCCTCGCGC
GTTTCGGTGATGACGGTGAAAACCTCTGACACATGCAGCTCCCGGAGACGGTCACAGCTTGT
CTGTAAGCGGATGCCGGGAGCAGACAAGCCCGTCAGGGCGCGTCAGCGGGTGTTGGCGGGT
GTCGGGGCGCAGCCATGACCCAGTCACGTAGCGATAGCGGAGTGTATAATTCTTGAAGACGA
AAGGGCCTCGTGATACGCCTATTTTTATAGGTTAATGTCATGATAATAATGGTTTCTTAGACG
TCAGGTGGCACTTTTCGGGGAAATGTGCGCGGAACCCCTATTTGTTTATTTTTCTAAATACAT
TCAAATATGTATCCGCTCATGAGACAATAACCCTGATAAATGCTTCAATAATATTGAAAAAG
GAAGAGTATGAGTATTCAACATTTCCGTGTCGCCCTTATTCCCTTTTTTGCGGCATTTTGCCTT
CCTGTTTTTGCTCACCCAGAAACGCTGGTGAAAGTAAAAGATGCTGAAGATCAGTTGGGTGC

ACGAGTGGGTTACATCGAACTGGATCTCAACAGCGGTAAGATCCTTGAGAGTTTTCGCCCCG
AAGAACGTTTTCCAATGATGAGCACTTTTAAAGTTCTGCTATGTGGCGCGGTATTATCCCGTG
TTGACGCCGGGCAAGAGCAACTCGGTCGCCGCATACACTATTCTCAGAATGACTTGGTTGAG
TACTCACCAGTCACAGAAAAGCATCTTACGGATGGCATGACAGTAAGAGAATTATGCAGTGC
TGCCATAACCATGAGTGATAACACTGCGGCCAACTTACTTCTGACAACGATCGGAGGACCGA
AGGAGCTAACCGCTTTTTTGCACAACATGGGGGATCATGTAACTCGCCTTGATCGTTGGGAA
CCGGAGCTGAATGAAGCCATACCAAACGACGAGCGTGACACCACGATGCCTGCAGCAATGG
CAACAACGTTGCGCAAACTATTAACTGGCGAACTACTTACTCTAGCTTCCCGGCAACAATTA
ATAGACTGGATGGAGGCGGATAAAGTTGCAGGACCACTTCTGCGCTCGGCCCTTCCGGCTGG
CTGGTTTATTGCTGATAAATCTGGAGCCGGTGAGCGTGGGTCTCGCGGTATCATTGCAGCACT
GGGGCCAGATGGTAAGCCCTCCCGTATCGTAGTTATCTACACGACGGGGAGTCAGGCAACTA
TGGATGAACGAAATAGACAGATCGCTGAGATAGGTGCCTCACTGATTAAGCATTGGTAACTG
TCAGACCAAGTTTACTCATATATACTTTAGATTGATTTAAAACTTCATTTTTAATTTAAAAGG
ATCTAGGTGAAGATCCTTTTTGATAATCTCATGACCAAAATCCCTTAACGTGAGTTTTCGTTC
CACTGAGCGTCAGACCCCGTAGAAAAGATCAAAGGATCTTCTTGAGATCCTTTTTTTCTGCGC
GTAATCTGCTGCTTGCAAACAAAAAAACCACCGCTACCAGCGGTGGTTTGTTTGCCGGATCA
AGAGCTACCAACTCTTTTTCCGAAGGTAACTGGCTTCAGCAGAGCGCAGATACCAAATACTG
TCCTTCTAGTGTAGCCGTAGTTAGGCCACCACTTCAAGAACTCTGTAGCACCGCCTACATACC
TCGCTCTGCTAATCCTGTTACCAGTGGCTGCTGCCAGTGGCGATAAGTCGTGTCTTACCGGGT
TGGACTCAAGACGATAGTTACCGGATAAGGCGCAGCGGTCGGGCTGAACGGGGGGTTCGTG
CACACAGCCCAGCTTGGAGCGAACGACCTACACCGAACTGAGATACCTACAGCGTGAGCTAT
GAGAAAGCGCCACGCTTCCCGAAGGGAGAAAGGCGGACAGGTATCCGGTAAGCGGCAGGGT
CGGAACAGGAGAGCGCACGAGGGAGCTTCCAGGGGGAAACGCCTGGTATCTTTATAGTCCT
GTCGGGTTTCGCCACCTCTGACTTGAGCGTCGATTTTTGTGATGCTCGTCAGGGGGGCGGAGC
CTATGGAAAAACGCCAGCAACGCGGCCTTTTTACGGTTCCTGGCCTTTTGCTGGCCTTTTGCT
CACATGTTCTTTCCTGCGTTATCCCCTGATTCTGTGGATAACCGTATTACCGCCTTTGAGTGAG
CTGATACCGCTCGCCGCAGCCGAACGACCGAGCGCAGCGAGTCAGTGAGCGAGGAAGCGGA
AGAGCGCCTGATGCGGTATTTTCTCCTTACGCATCTGTGCGGTATTTCACACCGCATAAATTC
CGACACCATCGAATGGTGCAAAACCTTTCGCGGTATGGCATGATAGCGCCCGGAAGAGAGTC
AATTCAGGGTGGTGAATGTGAAACCAGTAACGTTATACGATGTCGCAGAGTATGCCGGTGTC
TCTTATCAGACCGTTTCCCGCGTGGTGAACCAGGCCAGCCACGTTTCTGCGAAAACGCGGGA
AAAAGTGGAAGCGGCGATGGCGGAGCTGAATTACATTCCCAACCGCGTGGCACAACAACTG
GCGGGCAAACAGTCGTTGCTGATTGGCGTTGCCACCTCCAGTCTGGCCCTGCACGCGCCGTC
GCAAATTGTCGCGGCGATTAAATCTCGCGCCGATCAACTGGGTGCCAGCGTGGTGGTGTCGA

TGGTAGAACGAAGCGGCGTCGAAGCCTGTAAAGCGGCGGTGCACAATCTTCTCGCGCAACGC
GTCAGTGGGCTGATCATTAACTATCCGCTGGATGACCAGGATGCCATTGCTGTGGAAGCTGC
CTGCACTAATGTTCCGGCGTTATTTCTTGATGTCTCTGACCAGACACCCATCAACAGTATTAT
TTTCTCCCATGAAGACGGTACGCGACTGGGCGTGGAGCATCTGGTCGCATTGGGTCACCAGC
AAATCGCGCTGTTAGCGGGCCCATTAAGTTCTGTCTCGGCGCGTCTGCGTCTGGCTGGCTGGC
ATAAATATCTCACTCGCAATCAAATTCAGCCGATAGCGGAACGGGAAGGCGACTGGAGTGCC
ATGTCCGGTTTTCAACAAACCATGCAAATGCTGAATGAGGGCATCGTTCCCACTGCGATGCT
GGTTGCCAACGATCAGATGGCGCTGGGCGCAATGCGCGCCATTACCGAGTCCGGGCTGCGCG
TTGGTGCGGATATCTCGGTAGTGGGATACGACGATACCGAAGACAGCTCATGTTATATCCCG
CCGTTAACCACCATCAAACAGGATTTTCGCCTGCTGGGGCAAACCAGCGTGGACCGCTTGCT
GCAACTCTCTCAGGGCCAGGCGGTGAAGGGCAATCAGCTGTTGCCCGTCTCACTGGTGAAAA
GAAAAACCACCCTGGCGCCCAATACGCAAACCGCCTCTCCCCGCGCGTTGGCCGATTCATTA
ATGCAGCTGGCACGACAGGTTTCCCGACTGGAAAGCGGGCAGTGAGCGCAACGCAATTAAT
GTGAGTTAGCTCACTCATTAGGCACCCCAGGCTTTACACTTTATGCTTCCGGCTCGTATGTTG
TGTGGAATTGTGAGCGGATAACAATTTCACACAGGAAACAGCTATGACCATGATTACGGATT
CACTGGCCGTCGTTTTACAACGTCGTGACTGGGAAAACCCTGGCGTTACCCAACTTAATCGCC
TTGCAGCACATCCCCCTTTCGCCAGCTGGCGTAATAGCGAAGAGGCCCGCACCGATCGCCCT
TCCCAACAGTTGCGCAGCCTGAATGGCGAATGGCGCTTTGCCTGGTTTCCGGCACCAGAAGC
GGTGCCGGAAAGCTGGCTGGAGTGCGATCTTCCTGAGGCCGATACTGTCGTCGTCCCCTCAA
ACTGGCAGATGCACGGTTACGATGCGCCCATCTACACCAACGTAACCTATCCCATTACGGTC
AATCCGCCGTTTGTTCCCACGGAGAATCCGACGGGTTGTTACTCGCTCACATTTAATGTTGAT
GAAAGCTGGCTACAGGAAGGCCAGACGCGAATTATTTTTGATGGCGTTGGAATT

b. pGEXosmY

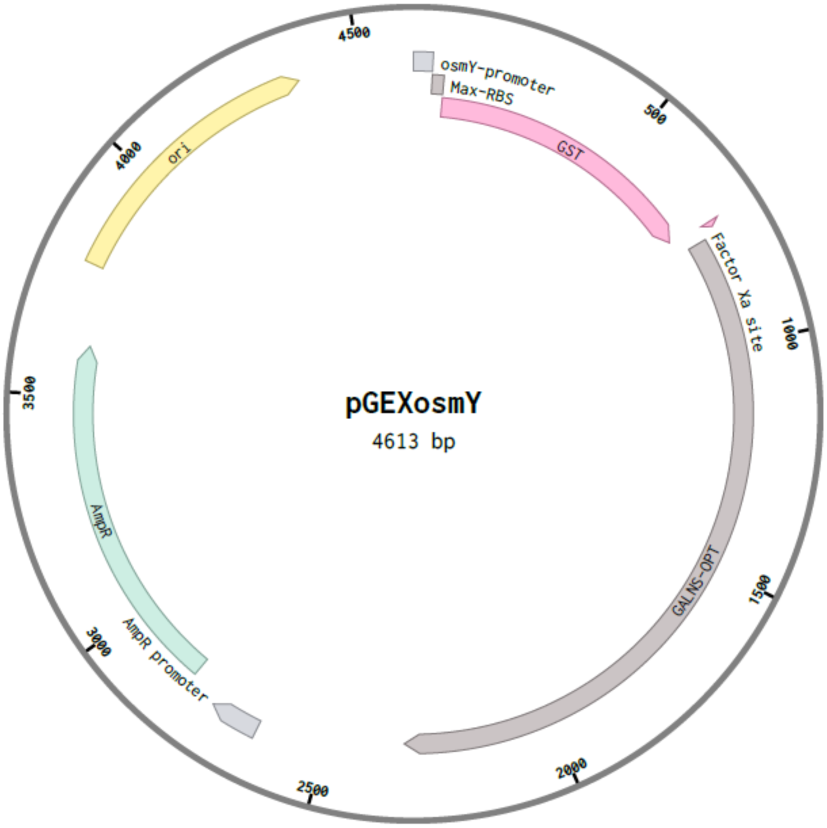

TATCCCGAGCGGTTTCAAAATTGTGATCTATATTTAACAAACGAAATCAACAAAAGCGGTTA
CTAACATGTCCCCTATACTAGGTTATTGGAAAATTAAGGGCCTTGTGCAACCCACTCGACTTC
TTTTGGAATATCTTGAAGAAAAATATGAAGAGCATTTGTATGAGCGCGATGAAGGTGATAAA
TGGCGAAACAAAAAGTTTGAATTGGGTTTGGAGTTTCCCAATCTTCCTTATTATATTGATGGT
GATGTTAAATTAACACAGTCTATGGCCATCATACGTTATATAGCTGACAAGCACAACATGTT
GGGTGGTTGTCCAAAAGAGCGTGCAGAGATTTCAATGCTTGAAGGAGCGGTTTTGGATATTA
GATACGGTGTTTCGAGAATTGCATATAGTAAAGACTTTGAAACTCTCAAAGTTGATTTTCTTA
GCAAGCTACCTGAAATGCTGAAAATGTTCGAAGATCGTTTATGTCATAAAACATATTTAAAT
GGTGATCATGTAACCCATCCTGACTTCATGTTGTATGACGCTCTTGATGTTGTTTTATACATG
GACCCAATGTGCCTGGATGCGTTCCCAAAATTAGTTTGTTTTAAAAAACGTATTGAAGCTATC
CCACAAATTGATAAGTACTTGAAATCCAGCAAGTATATAGCATGGCCTTTGCAGGGCTGGCA
AGCCACGTTTGGTGGTGGCGACCATCCTCCAAAATCGGATCTGATCGAAGGTCGTGGGATCC
CCGAATTCGATGGCAGCAGTTGTTGCAGCAACCCGTTGGTGGCAGCTGCTGCTGGTTCTGAG
CGCAGCAGGTATGGGTGCAAGTGGTGCACCGCAGCCTCCGAATATTCTGCTGCTGCTGATGG
ATGATATGGGTTGGGGTGATCTGGGTGTTTATGGTGAACCGAGCCGTGAAACCCCGAATCTG
GATCGTATGGCAGCAGAAGGTCTGCTGTTTCCGAACTTTTATAGCGCAAATCCGCTGAGCAG

CCCGAGCCGTGCAGCACTGCTGACAGGTCGTCTGCCGATTCGTAATGGTTTTTATACCACCAA
TGCACATGCACGCAATGCATATACACCGCAAGAAATTGTTGGTGGTATTCCGGATAGCGAAC
AGCTGCTGCCGGAACTGCTGAAAAAAGCAGGTTATGTTAGCAAAATTGTGGGCAAATGGCAT
CTGGGTCATCGTCCGCAGTTTCATCCGCTGAAACATGGTTTTGATGAATGGTTTGGTAGCCCG
AATTGTCATTTTGGTCCGTATGATAATAAAGCCCGTCCGAATATTCCGGTTTATCGTGATTGG
GAAATGGTGGGTCGTTATTATGAAGAATTTCCGATCAATCTGAAAACCGGTGAAGCAAATCT
GACCCAGATTTATCTGCAAGAAGCACTGGATTTCATTAAACGTCAGGCACGTCATCATCCGTT
TTTTCTGTATTGGGCAGTTGATGCAACCCATGCACCGGTTTATGCAAGCAAACCGTTTCTGGG
CACCAGCCAGCGTGGTCGTTATGGTGATGCAGTTCGTGAAATTGATGATAGCATTGGCAAAA
TTCTGGAACTGCTGCAGGATCTGCATGTTGCAGATAATACCTTTGTGTTTTTCACCAGCGATA
ATGGTGCAGCACTGATTAGCGCACCGGAACAGGGTGGTAGCAATGGTCCGTTTCTGTGTGGT
AAACAGACCACCTTTGAAGGTGGTATGCGTGAACCGGCACTGGCATGGTGGCCTGGTCATGT
TACCGCAGGTCAGGTTAGCCATCAGCTGGGTAGCATTATGGACCTGTTTACCACCAGTCTGG
CACTGGCAGGTCTGACCCCTCCGAGCGATCGTGCAATTGATGGTCTGAATCTGCTGCCGACA
CTGCTGCAGGGACGTCTGATGGATCGTCCGATTTTCTATTATCGTGGTGATACCCTGATGGCA
GCAACCCTGGGTCAGCATAAAGCACATTTTTGGACCTGGACCAATAGCTGGGAAAATTTTCG
TCAGGGCATTGATTTTTGTCCGGGTCAGAATGTTAGCGGTGTTACCACCCATAATCTGGAAGA
TCATACCAAACTGCCGCTGATTTTTCATCTGGGTCGTGATCCGGGTGAACGTTTTCCGCTGAG
CTTTGCAAGCGCAGAATATCAAGAAGCCCTGAGCCGTATTACCAGCGTTGTTCAGCAGCATC
AAGAAGCGCTGGTTCCGGCACAGCCGCAGCTGAATGTTTGTAATTGGGCAGTTATGAATTGG
GCACCGCCTGGTTGTGAAAAACTGGGTAAATGTCTGACCCCACCGGAAAGCATTCCGAAAAA
ATGTCTGTGGTCACATTAACTCGAGCGGCCGCATCGTGACTGACTGACGATCTGCCTCGCGC
GTTTCGGTGATGACGGTGAAAACCTCTGACACATGCAGCTCCCGGAGACGGTCACAGCTTGT
CTGTAAGCGGATGCCGGGAGCAGACAAGCCCGTCAGGGCGCGTCAGCGGGTGTTGGCGGGT
GTCGGGGCGCAGCCATGACCCAGTCACGTAGCGATAGCGGAGTGTATAATTCTTGAAGACGA
AAGGGCCTCGTGATACGCCTATTTTTATAGGTTAATGTCATGATAATAATGGTTTCTTAGACG
TCAGGTGGCACTTTTCGGGGAAATGTGCGCGGAACCCCTATTTGTTTATTTTTCTAAATACAT
TCAAATATGTATCCGCTCATGAGACAATAACCCTGATAAATGCTTCAATAATATTGAAAAAG
GAAGAGTATGAGTATTCAACATTTCCGTGTCGCCCTTATTCCCTTTTTTGCGGCATTTTGCCTT
CCTGTTTTTGCTCACCCAGAAACGCTGGTGAAAGTAAAAGATGCTGAAGATCAGTTGGGTGC
ACGAGTGGGTTACATCGAACTGGATCTCAACAGCGGTAAGATCCTTGAGAGTTTTCGCCCCG
AAGAACGTTTTCCAATGATGAGCACTTTTAAAGTTCTGCTATGTGGCGCGGTATTATCCCGTG
TTGACGCCGGGCAAGAGCAACTCGGTCGCCGCATACACTATTCTCAGAATGACTTGGTTGAG
TACTCACCAGTCACAGAAAAGCATCTTACGGATGGCATGACAGTAAGAGAATTATGCAGTGC

TGCCATAACCATGAGTGATAACACTGCGGCCAACTTACTTCTGACAACGATCGGAGGACCGA
AGGAGCTAACCGCTTTTTTGCACAACATGGGGGATCATGTAACTCGCCTTGATCGTTGGGAA
CCGGAGCTGAATGAAGCCATACCAAACGACGAGCGTGACACCACGATGCCTGCAGCAATGG
CAACAACGTTGCGCAAACTATTAACTGGCGAACTACTTACTCTAGCTTCCCGGCAACAATTA
ATAGACTGGATGGAGGCGGATAAAGTTGCAGGACCACTTCTGCGCTCGGCCCTTCCGGCTGG
CTGGTTTATTGCTGATAAATCTGGAGCCGGTGAGCGTGGGTCTCGCGGTATCATTGCAGCACT
GGGGCCAGATGGTAAGCCCTCCCGTATCGTAGTTATCTACACGACGGGGAGTCAGGCAACTA
TGGATGAACGAAATAGACAGATCGCTGAGATAGGTGCCTCACTGATTAAGCATTGGTAACTG
TCAGACCAAGTTTACTCATATATACTTTAGATTGATTTAAAACTTCATTTTTAATTTAAAAGG
ATCTAGGTGAAGATCCTTTTTGATAATCTCATGACCAAAATCCCTTAACGTGAGTTTTCGTTC
CACTGAGCGTCAGACCCCGTAGAAAAGATCAAAGGATCTTCTTGAGATCCTTTTTTTCTGCGC
GTAATCTGCTGCTTGCAAACAAAAAAACCACCGCTACCAGCGGTGGTTTGTTTGCCGGATCA
AGAGCTACCAACTCTTTTTCCGAAGGTAACTGGCTTCAGCAGAGCGCAGATACCAAATACTG
TCCTTCTAGTGTAGCCGTAGTTAGGCCACCACTTCAAGAACTCTGTAGCACCGCCTACATACC
TCGCTCTGCTAATCCTGTTACCAGTGGCTGCTGCCAGTGGCGATAAGTCGTGTCTTACCGGGT
TGGACTCAAGACGATAGTTACCGGATAAGGCGCAGCGGTCGGGCTGAACGGGGGGTTCGTG
CACACAGCCCAGCTTGGAGCGAACGACCTACACCGAACTGAGATACCTACAGCGTGAGCTAT
GAGAAAGCGCCACGCTTCCCGAAGGGAGAAAGGCGGACAGGTATCCGGTAAGCGGCAGGGT
CGGAACAGGAGAGCGCACGAGGGAGCTTCCAGGGGGAAACGCCTGGTATCTTTATAGTCCT
GTCGGGTTTCGCCACCTCTGACTTGAGCGTCGATTTTTGTGATGCTCGTCAGGGGGGCGGAGC
CTATGGAAAAACGCCAGCAACGCGGCCTTTTTACGGTTCCTGGCCTTTTGCTGGCCTTTTGCT
CACATGTTCTTTCCTGCGTTATCCCCTGATTCTGTGGATAACCGTATTACCGCCTTTGAGTGAG
CTGATACCGCTCGCCGCAGCCGAACGACCGAGCGCAGCGAGTCAGTGAGCGAGGAAGCGGA
AGAGCGCCTGATGCGGTATTTTCTCCTTACGCATCTGTGCGGTATTTCACACCGCATAAATTC
C

c. pGEXproUmod

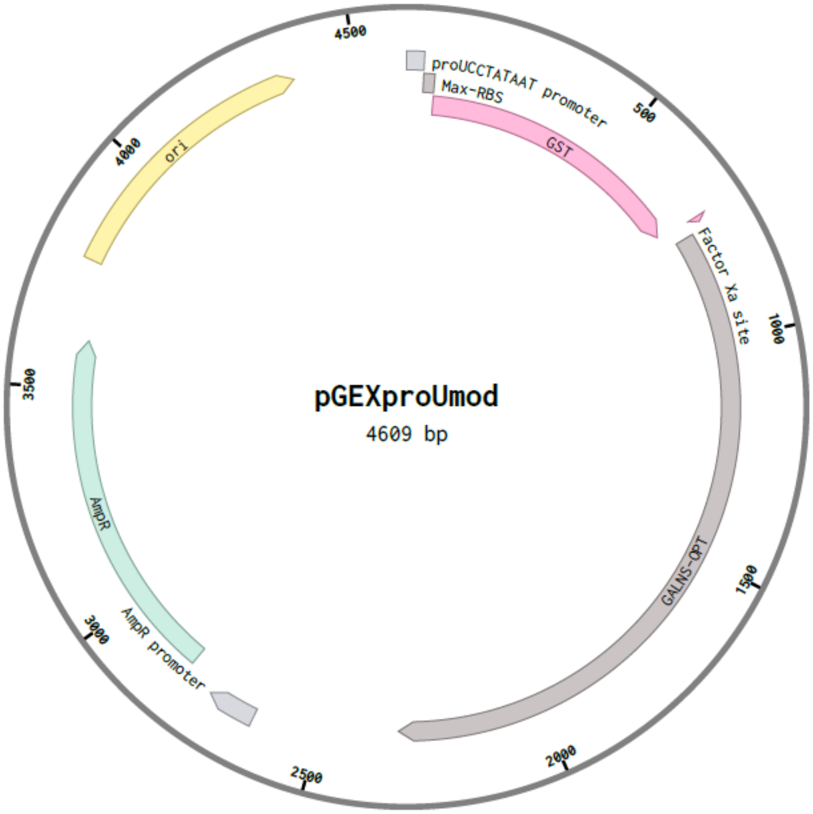

GGGGCCGCCTCAGATTCTCAGTATGTTATAATAGAAAAGCGAACGGAAATCTACGGTTAACA
TATGTCCCCTATACTAGGTTATTGGAAAATTAAGGGCCTTGTGCAACCCACTCGACTTCTTTT
GGAATATCTTGAAGAAAAATATGAAGAGCATTTGTATGAGCGCGATGAAGGTGATAAATGG
CGAAACAAAAAGTTTGAATTGGGTTTGGAGTTTCCCAATCTTCCTTATTATATTGATGGTGAT
GTTAAATTAACACAGTCTATGGCCATCATACGTTATATAGCTGACAAGCACAACATGTTGGG
TGGTTGTCCAAAAGAGCGTGCAGAGATTTCAATGCTTGAAGGAGCGGTTTTGGATATTAGAT
ACGGTGTTTCGAGAATTGCATATAGTAAAGACTTTGAAACTCTCAAAGTTGATTTTCTTAGCA
AGCTACCTGAAATGCTGAAAATGTTCGAAGATCGTTTATGTCATAAAACATATTTAAATGGT
GATCATGTAACCCATCCTGACTTCATGTTGTATGACGCTCTTGATGTTGTTTTATACATGGAC
CCAATGTGCCTGGATGCGTTCCCAAAATTAGTTTGTTTTAAAAAACGTATTGAAGCTATCCCA
CAAATTGATAAGTACTTGAAATCCAGCAAGTATATAGCATGGCCTTTGCAGGGCTGGCAAGC
CACGTTTGGTGGTGGCGACCATCCTCCAAAATCGGATCTGATCGAAGGTCGTGGGATCCCCG
AATTCGATGGCAGCAGTTGTTGCAGCAACCCGTTGGTGGCAGCTGCTGCTGGTTCTGAGCGC
AGCAGGTATGGGTGCAAGTGGTGCACCGCAGCCTCCGAATATTCTGCTGCTGCTGATGGATG
ATATGGGTTGGGGTGATCTGGGTGTTTATGGTGAACCGAGCCGTGAAACCCCGAATCTGGAT
CGTATGGCAGCAGAAGGTCTGCTGTTTCCGAACTTTTATAGCGCAAATCCGCTGAGCAGCCC

GAGCCGTGCAGCACTGCTGACAGGTCGTCTGCCGATTCGTAATGGTTTTATACCACCAATGC
ACATGCACGCAATGCATATACACCGCAAGAAATTGTTGGTGGTATTCCGGATAGCGAACAGC
TGCTGCCGGAACTGCTGAAAAAAGCAGGTTATGTTAGCAAAATTGTGGGCAAATGGCATCTG
GGTCATCGTCCGCAGTTTCATCCGCTGAAACATGGTTTTGATGAATGGTTTGGTAGCCCGAAT
TGTCATTTTGGTCCGTATGATAATAAAGCCCGTCCGAATATTCCGGTTTATCGTGATTGGGAA
ATGGTGGGTCGTTATTATGAAGAATTTCCGATCAATCTGAAAACCGGTGAAGCAAATCTGAC
CCAGATTTATCTGCAAGAAGCACTGGATTTCATTAAACGTCAGGCACGTCATCATCCGTTTTT
TCTGTATTGGGCAGTTGATGCAACCCATGCACCGGTTTATGCAAGCAAACCGTTTCTGGGCAC
CAGCCAGCGTGGTCGTTATGGTGATGCAGTTCGTGAAATTGATGATAGCATTGGCAAAATTC
TGGAACTGCTGCAGGATCTGCATGTTGCAGATAATACCTTTGTGTTTTTCACCAGCGATAATG
GTGCAGCACTGATTAGCGCACCGGAACAGGGTGGTAGCAATGGTCCGTTTCTGTGTGGTAAA
CAGACCACCTTTGAAGGTGGTATGCGTGAACCGGCACTGGCATGGTGGCCTGGTCATGTTAC
CGCAGGTCAGGTTAGCCATCAGCTGGGTAGCATTATGGACCTGTTTACCACCAGTCTGGCAC
TGGCAGGTCTGACCCCTCCGAGCGATCGTGCAATTGATGGTCTGAATCTGCTGCCGACACTG
CTGCAGGGACGTCTGATGGATCGTCCGATTTTCTATTATCGTGGTGATACCCTGATGGCAGCA
ACCCTGGGTCAGCATAAAGCACATTTTTGGACCTGGACCAATAGCTGGGAAAATTTTCGTCA
GGGCATTGATTTTTGTCCGGGTCAGAATGTTAGCGGTGTTACCACCCATAATCTGGAAGATCA
TACCAAACTGCCGCTGATTTTTCATCTGGGTCGTGATCCGGGTGAACGTTTTCCGCTGAGCTT
TGCAAGCGCAGAATATCAAGAAGCCCTGAGCCGTATTACCAGCGTTGTTCAGCAGCATCAAG
AAGCGCTGGTTCCGGCACAGCCGCAGCTGAATGTTTGTAATTGGGCAGTTATGAATTGGGCA
CCGCCTGGTTGTGAAAAACTGGGTAAATGTCTGACCCCACCGGAAAGCATTCCGAAAAAATG
TCTGTGGTCACATTAACTCGAGCGGCCGCATCGTGACTGACTGACGATCTGCCTCGCGCGTTT
CGGTGATGACGGTGAAAACCTCTGACACATGCAGCTCCCGGAGACGGTCACAGCTTGTCTGT
AAGCGGATGCCGGGAGCAGACAAGCCCGTCAGGGCGCGTCAGCGGGTGTTGGCGGGTGTCG
GGGCGCAGCCATGACCCAGTCACGTAGCGATAGCGGAGTGTATAATTCTTGAAGACGAAAG
GGCCTCGTGATACGCCTATTTTTATAGGTTAATGTCATGATAATAATGGTTTCTTAGACGTCA
GGTGGCACTTTTCGGGGAAATGTGCGCGGAACCCCTATTTGTTTATTTTTCTAAATACATTCA
AATATGTATCCGCTCATGAGACAATAACCCTGATAAATGCTTCAATAATATTGAAAAAGGAA
GAGTATGAGTATTCAACATTTCCGTGTCGCCCTTATTCCCTTTTTTGCGGCATTTTGCCTTCCT
GTTTTTGCTCACCCAGAAACGCTGGTGAAAGTAAAAGATGCTGAAGATCAGTTGGGTGCACG
AGTGGGTTACATCGAACTGGATCTCAACAGCGGTAAGATCCTTGAGAGTTTTCGCCCCGAAG
AACGTTTTCCAATGATGAGCACTTTTAAAGTTCTGCTATGTGGCGCGGTATTATCCCGTGTTG
ACGCCGGGCAAGAGCAACTCGGTCGCCGCATACACTATTCTCAGAATGACTTGGTTGAGTAC
TCACCAGTCACAGAAAAGCATCTTACGGATGGCATGACAGTAAGAGAATTATGCAGTGCTGC

CATAACCATGAGTGATAACACTGCGGCCAACTTACTTCTGACAACGATCGGAGGACCGAAGG
AGCTAACCGCTTTTTTGCACAACATGGGGGATCATGTAACTCGCCTTGATCGTTGGGAACCG
GAGCTGAATGAAGCCATACCAAACGACGAGCGTGACACCACGATGCCTGCAGCAATGGCAA
CAACGTTGCGCAAACTATTAACTGGCGAACTACTTACTCTAGCTTCCCGGCAACAATTAATA
GACTGGATGGAGGCGGATAAAGTTGCAGGACCACTTCTGCGCTCGGCCCTTCCGGCTGGCTG
GTTTATTGCTGATAAATCTGGAGCCGGTGAGCGTGGGTCTCGCGGTATCATTGCAGCACTGG
GGCCAGATGGTAAGCCCTCCCGTATCGTAGTTATCTACACGACGGGGAGTCAGGCAACTATG
GATGAACGAAATAGACAGATCGCTGAGATAGGTGCCTCACTGATTAAGCATTGGTAACTGTC
AGACCAAGTTTACTCATATATACTTTAGATTGATTTAAAACTTCATTTTTAATTTAAAAGGAT
CTAGGTGAAGATCCTTTTTGATAATCTCATGACCAAAATCCCTTAACGTGAGTTTTCGTTCCA
CTGAGCGTCAGACCCCGTAGAAAAGATCAAAGGATCTTCTTGAGATCCTTTTTTTCTGCGCGT
AATCTGCTGCTTGCAAACAAAAAAACCACCGCTACCAGCGGTGGTTTGTTTGCCGGATCAAG
AGCTACCAACTCTTTTTCCGAAGGTAACTGGCTTCAGCAGAGCGCAGATACCAAATACTGTC
CTTCTAGTGTAGCCGTAGTTAGGCCACCACTTCAAGAACTCTGTAGCACCGCCTACATACCTC
GCTCTGCTAATCCTGTTACCAGTGGCTGCTGCCAGTGGCGATAAGTCGTGTCTTACCGGGTTG
GACTCAAGACGATAGTTACCGGATAAGGCGCAGCGGTCGGGCTGAACGGGGGGTTCGTGCA
CACAGCCCAGCTTGGAGCGAACGACCTACACCGAACTGAGATACCTACAGCGTGAGCTATGA
GAAAGCGCCACGCTTCCCGAAGGGAGAAAGGCGGACAGGTATCCGGTAAGCGGCAGGGTCG
GAACAGGAGAGCGCACGAGGGAGCTTCCAGGGGGAAACGCCTGGTATCTTTATAGTCCTGTC
GGGTTTCGCCACCTCTGACTTGAGCGTCGATTTTTGTGATGCTCGTCAGGGGGGCGGAGCCTA
TGGAAAAACGCCAGCAACGCGGCCTTTTTACGGTTCCTGGCCTTTTGCTGGCCTTTTGCTCAC
ATGTTCTTTCCTGCGTTATCCCCTGATTCTGTGGATAACCGTATTACCGCCTTTGAGTGAGCTG
ATACCGCTCGCCGCAGCCGAACGACCGAGCGCAGCGAGTCAGTGAGCGAGGAAGCGGAAGA
GCGCCTGATGCGGTATTTTCTCCTTACGCATCTGTGCGGTATTTCACACCGCATAAATTCC

d. pACYCDuet™-1

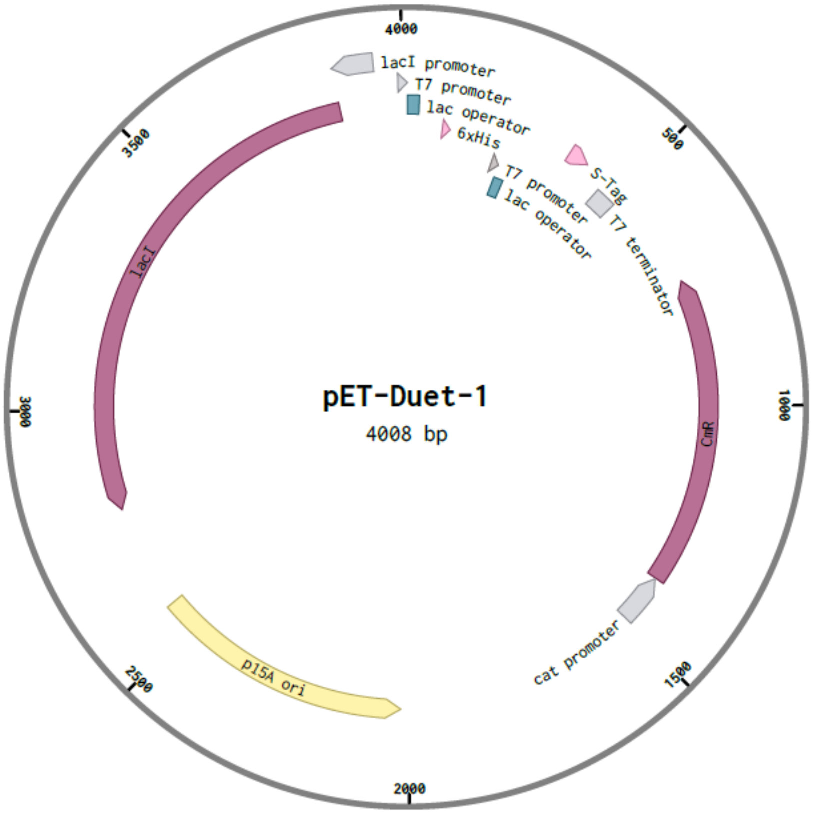

GGGGAATTGTGAGCGGATAACAATTCCCCTGTAGAAATAATTTTGTTTAACTTTAATAAGGA
GATATACCATGGGCAGCAGCCATCACCATCATCACCACAGCCAGGATCCGAATTCGAGCTCG
GCGCGCCTGCAGGTCGACAAGCTTGCGGCCGCATAATGCTTAAGTCGAACAGAAAGTAATCG
TATTGTACACGGCCGCATAATCGAAATTAATACGACTCACTATAGGGGAATTGTGAGCGGAT
AACAATTCCCCATCTTAGTATATTAGTTAAGTATAAGAAGGAGATATACATATGGCAGATCT
CAATTGGATATCGGCCGGCCACGCGATCGCTGACGTCGGTACCCTCGAGTCTGGTAAAGAAA
CCGCTGCTGCGAAATTTGAACGCCAGCACATGGACTCGTCTACTAGCGCAGCTTAATTAACC
TAGGCTGCTGCCACCGCTGAGCAATAACTAGCATAACCCCTTGGGGCCTCTAAACGGGTCTT
GAGGGGTTTTTTGCTGAAACCTCAGGCATTTGAGAAGCACACGGTCACACTGCTTCCGGTAG
TCAATAAACCGGTAAACCAGCAATAGACATAAGCGGCTATTTAACGACCCTGCCCTGAACCG
ACGACCGGGTCGAATTTGCTTTCGAATTTCTGCCATTCATCCGCTTATTATCACTTATTCAGGC
GTAGCACCAGGCGTTTAAGGGCACCAATAACTGCCTTAAAAAAATTACGCCCCGCCCTGCCA
CTCATCGCAGTACTGTTGTAATTCATTAAGCATTCTGCCGACATGGAAGCCATCACAGACGG
CATGATGAACCTGAATCGCCAGCGGCATCAGCACCTTGTCGCCTTGCGTATAATATTTGCCCA
TAGTGAAAACGGGGGCGAAGAAGTTGTCCATATTGGCCACGTTTAAATCAAAACTGGTGAAA
CTCACCCAGGGATTGGCTGAGACGAAAAACATATTCTCAATAAACCCTTTAGGGAAATAGGC

CAGGTTTTCACCGTAACACGCCACATCTTGCGAATATATGTGTAGAAACTGCCGGAAATCGT
CGTGGTATTCACTCCAGAGCGATGAAAACGTTTCAGTTTGCTCATGGAAAACGGTGTAACAA
GGGTGAACACTATCCCATATCACCAGCTCACCGTCTTTCATTGCCATACGGAACTCCGGATGA
GCATTCATCAGGCGGGCAAGAATGTGAATAAAGGCCGGATAAAACTTGTGCTTATTTTTCTTT
ACGGTCTTTAAAAAGGCCGTAATATCCAGCTGAACGGTCTGGTTATAGGTACATTGAGCAAC
TGACTGAAATGCCTCAAAATGTTCTTTACGATGCCATTGGGATATATCAACGGTGGTATATCC
AGTGATTTTTTTCTCCATTTTAGCTTCCTTAGCTCCTGAAAATCTCGATAACTCAAAAAATAC
GCCCGGTAGTGATCTTATTTCATTATGGTGAAAGTTGGAACCTCTTACGTGCCGATCAACGTC
TCATTTTCGCCAAAAGTTGGCCCAGGGCTTCCCGGTATCAACAGGGACACCAGGATTTATTTA
TTCTGCGAAGTGATCTTCCGTCACAGGTATTTATTCGGCGCAAAGTGCGTCGGGTGATGCTGC
CAACTTACTGATTTAGTGTATGATGGTGTTTTTGAGGTGCTCCAGTGGCTTCTGTTTCTATCAG
CTGTCCCTCCTGTTCAGCTACTGACGGGGTGGTGCGTAACGGCAAAAGCACCGCCGGACATC
AGCGCTAGCGGAGTGTATACTGGCTTACTATGTTGGCACTGATGAGGGTGTCAGTGAAGTGC
TTCATGTGGCAGGAGAAAAAAGGCTGCACCGGTGCGTCAGCAGAATATGTGATACAGGATA
TATTCCGCTTCCTCGCTCACTGACTCGCTACGCTCGGTCGTTCGACTGCGGCGAGCGGAAATG
GCTTACGAACGGGGCGGAGATTTCCTGGAAGATGCCAGGAAGATACTTAACAGGGAAGTGA
GAGGGCCGCGGCAAAGCCGTTTTTCCATAGGCTCCGCCCCCCTGACAAGCATCACGAAATCT
GACGCTCAAATCAGTGGTGGCGAAACCCGACAGGACTATAAAGATACCAGGCGTTTCCCCTG
GCGGCTCCCTCGTGCGCTCTCCTGTTCCTGCCTTTCGGTTTACCGGTGTCATTCCGCTGTTATG
GCCGCGTTTGTCTCATTCCACGCCTGACACTCAGTTCCGGGTAGGCAGTTCGCTCCAAGCTGG
ACTGTATGCACGAACCCCCCGTTCAGTCCGACCGCTGCGCCTTATCCGGTAACTATCGTCTTG
AGTCCAACCCGGAAAGACATGCAAAAGCACCACTGGCAGCAGCCACTGGTAATTGATTTAG
AGGAGTTAGTCTTGAAGTCATGCGCCGGTTAAGGCTAAACTGAAAGGACAAGTTTTGGTGAC
TGCGCTCCTCCAAGCCAGTTACCTCGGTTCAAAGAGTTGGTAGCTCAGAGAACCTTCGAAAA
ACCGCCCTGCAAGGCGGTTTTTTCGTTTTCAGAGCAAGAGATTACGCGCAGACCAAAACGAT
CTCAAGAAGATCATCTTATTAATCAGATAAAATATTTCTAGATTTCAGTGCAATTTATCTCTT
CAAATGTAGCACCTGAAGTCAGCCCCATACGATATAAGTTGTAATTCTCATGTTAGTCATGCC
CCGCGCCCACCGGAAGGAGCTGACTGGGTTGAAGGCTCTCAAGGGCATCGGTCGAGATCCCG
GTGCCTAATGAGTGAGCTAACTTACATTAATTGCGTTGCGCTCACTGCCCGCTTTCCAGTCGG
GAAACCTGTCGTGCCAGCTGCATTAATGAATCGGCCAACGCGCGGGGAGAGGCGGTTTGCGT
ATTGGGCGCCAGGGTGGTTTTTCTTTTCACCAGTGAGACGGGCAACAGCTGATTGCCCTTCAC
CGCCTGGCCCTGAGAGAGTTGCAGCAAGCGGTCCACGCTGGTTTGCCCCAGCAGGCGAAAAT
CCTGTTTGATGGTGGTTAACGGCGGGATATAACATGAGCTGTCTTCGGTATCGTCGTATCCCA
CTACCGAGATGTCCGCACCAACGCGCAGCCCGGACTCGGTAATGGCGCGCATTGCGCCCAGC

GCCATCTGATCGTTGGCAACCAGCATCGCAGTGGGAACGATGCCCTCATTCAGCATTTGCAT
GGTTTGTTGAAAACCGGACATGGCACTCCAGTCGCCTTCCCGTTCCGCTATCGGCTGAATTTG
ATTGCGAGTGAGATATTTATGCCAGCCAGCCAGACGCAGACGCGCCGAGACAGAACTTAATG
GGCCCGCTAACAGCGCGATTTGCTGGTGACCCAATGCGACCAGATGCTCCACGCCCAGTCGC
GTACCGTCTTCATGGGAGAAAATAATACTGTTGATGGGTGTCTGGTCAGAGACATCAAGAAA
TAACGCCGGAACATTAGTGCAGGCAGCTTCCACAGCAATGGCATCCTGGTCATCCAGCGGAT
AGTTAATGATCAGCCCACTGACGCGTTGCGCGAGAAGATTGTGCACCGCCGCTTTACAGGCT
TCGACGCCGCTTCGTTCTACCATCGACACCACCACGCTGGCACCCAGTTGATCGGCGCGAGA
TTTAATCGCCGCGACAATTTGCGACGGCGCGTGCAGGGCCAGACTGGAGGTGGCAACGCCAA
TCAGCAACGACTGTTTGCCCGCCAGTTGTTGTGCCACGCGGTTGGGAATGTAATTCAGCTCCG
CCATCGCCGCTTCCACTTTTTCCCGCGTTTTCGCAGAAACGTGGCTGGCCTGGTTCACCACGC
GGGAAACGGTCTGATAAGAGACACCGGCATACTCTGCGACATCGTATAACGTTACTGGTTTC
ACATTCACCACCCTGAATTGACTCTCTTCCGGGCGCTATCATGCCATACCGCGAAAGGTTTTG
CGCCATTCGATGGTGTCCGGGATCTCGACGCTCTCCCTTATGCGACTCCTGCATTAGGAAATT
AATACGACTCACTATA

e. pDuet::GroS

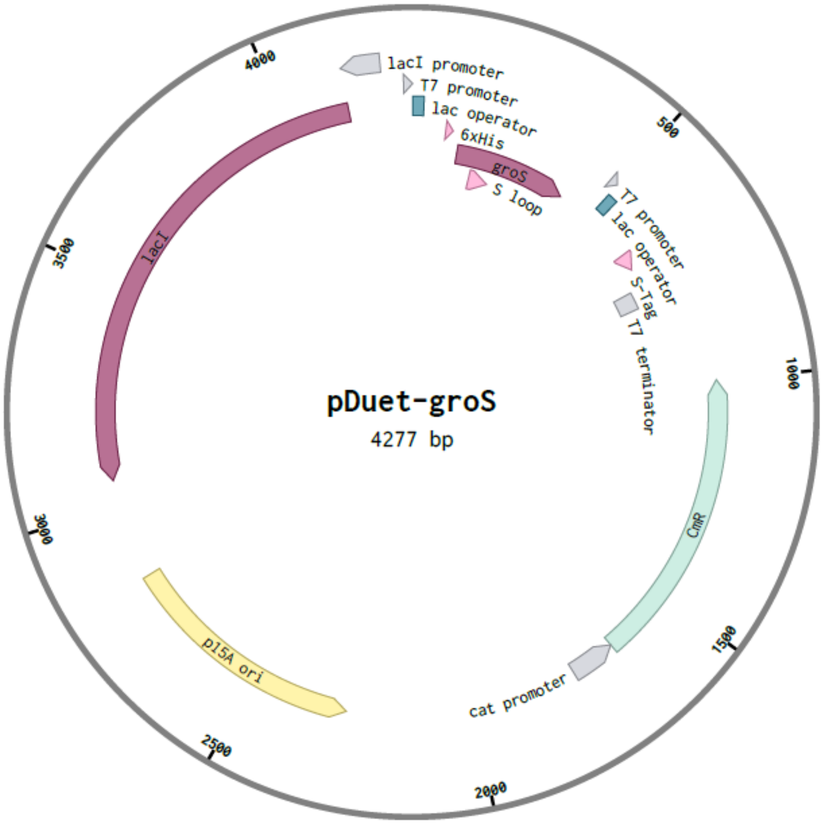

GGGGAATTGTGAGCGGATAACAATTCCCCTGTAGAAATAATTTTGTTTAACTTTAATAAGGA
GATATACCATGGGCAGCAGCCATCACCATCATCACCACAGCCAGGATCCGAATTCATGAATA
TTCGTCCATTGCATGATCGCGTGATCGTCAAGCGTAAAGAAGTTGAAACTAAATCTGCTGGC
GGCATCGTTCTGACCGGCTCTGCAGCGGCTAAATCCACCCGCGGCGAAGTGCTGGCTGTCGG
CAATGGCCGTATCCTTGAAAATGGCGAAGTGAAGCCGCTGGATGTGAAAGTTGGCGACATCG
TTATTTTCAACGATGGCTACGGTGTGAAATCTGAGAAGATCGACAATGAAGAAGTGTTGATC
ATGTCCGAAAGCGACATTCTGGCAATTGTTGAAGCGTAAAAGCTTGCGGCCGCATAATGCTT
AAGTCGAACAGAAAGTAATCGTATTGTACACGGCCGCATAATCGAAATTAATACGACTCACT
ATAGGGGAATTGTGAGCGGATAACAATTCCCCATCTTAGTATATTAGTTAAGTATAAGAAGG
AGATATACATATGGCAGATCTCAATTGGATATCGGCCGGCCACGCGATCGCTGACGTCGGTA
CCCTCGAGTCTGGTAAAGAAACCGCTGCTGCGAAATTTGAACGCCAGCACATGGACTCGTCT
ACTAGCGCAGCTTAATTAACCTAGGCTGCTGCCACCGCTGAGCAATAACTAGCATAACCCCT
TGGGGCCTCTAAACGGGTCTTGAGGGGTTTTTTGCTGAAACCTCAGGCATTTGAGAAGCACA
CGGTCACACTGCTTCCGGTAGTCAATAAACCGGTAAACCAGCAATAGACATAAGCGGCTATT
TAACGACCCTGCCCTGAACCGACGACCGGGTCGAATTTGCTTTCGAATTTCTGCCATTCATCC
GCTTATTATCACTTATTCAGGCGTAGCACCAGGCGTTTAAGGGCACCAATAACTGCCTTAAA

AAAATTACGCCCCGCCCTGCCACTCATCGCAGTACTGTTGTAATTCATTAAGCATTCTGCCGA
CATGGAAGCCATCACAGACGGCATGATGAACCTGAATCGCCAGCGGCATCAGCACCTTGTCG
CCTTGCGTATAATATTTGCCCATAGTGAAAACGGGGGCGAAGAAGTTGTCCATATTGGCCAC
GTTTAAATCAAAACTGGTGAAACTCACCCAGGGATTGGCTGAGACGAAAAACATATTCTCAA
TAAACCCTTTAGGGAAATAGGCCAGGTTTTCACCGTAACACGCCACATCTTGCGAATATATG
TGTAGAAACTGCCGGAAATCGTCGTGGTATTCACTCCAGAGCGATGAAAACGTTTCAGTTTG
CTCATGGAAAACGGTGTAACAAGGGTGAACACTATCCCATATCACCAGCTCACCGTCTTTCA
TTGCCATACGGAACTCCGGATGAGCATTCATCAGGCGGGCAAGAATGTGAATAAAGGCCGG
ATAAAACTTGTGCTTATTTTTCTTTACGGTCTTTAAAAAGGCCGTAATATCCAGCTGAACGGT
CTGGTTATAGGTACATTGAGCAACTGACTGAAATGCCTCAAAATGTTCTTTACGATGCCATTG
GGATATATCAACGGTGGTATATCCAGTGATTTTTTTCTCCATTTTAGCTTCCTTAGCTCCTGAA
AATCTCGATAACTCAAAAAATACGCCCGGTAGTGATCTTATTTCATTATGGTGAAAGTTGGA
ACCTCTTACGTGCCGATCAACGTCTCATTTTCGCCAAAAGTTGGCCCAGGGCTTCCCGGTATC
AACAGGGACACCAGGATTTATTTATTCTGCGAAGTGATCTTCCGTCACAGGTATTTATTCGGC
GCAAAGTGCGTCGGGTGATGCTGCCAACTTACTGATTTAGTGTATGATGGTGTTTTTGAGGTG
CTCCAGTGGCTTCTGTTTCTATCAGCTGTCCCTCCTGTTCAGCTACTGACGGGGTGGTGCGTA
ACGGCAAAAGCACCGCCGGACATCAGCGCTAGCGGAGTGTATACTGGCTTACTATGTTGGCA
CTGATGAGGGTGTCAGTGAAGTGCTTCATGTGGCAGGAGAAAAAAGGCTGCACCGGTGCGTC
AGCAGAATATGTGATACAGGATATATTCCGCTTCCTCGCTCACTGACTCGCTACGCTCGGTCG
TTCGACTGCGGCGAGCGGAAATGGCTTACGAACGGGGCGGAGATTTCCTGGAAGATGCCAG
GAAGATACTTAACAGGGAAGTGAGAGGGCCGCGGCAAAGCCGTTTTTCCATAGGCTCCGCCC
CCCTGACAAGCATCACGAAATCTGACGCTCAAATCAGTGGTGGCGAAACCCGACAGGACTAT
AAAGATACCAGGCGTTTCCCCTGGCGGCTCCCTCGTGCGCTCTCCTGTTCCTGCCTTTCGGTTT
ACCGGTGTCATTCCGCTGTTATGGCCGCGTTTGTCTCATTCCACGCCTGACACTCAGTTCCGG
GTAGGCAGTTCGCTCCAAGCTGGACTGTATGCACGAACCCCCCGTTCAGTCCGACCGCTGCG
CCTTATCCGGTAACTATCGTCTTGAGTCCAACCCGGAAAGACATGCAAAAGCACCACTGGCA
GCAGCCACTGGTAATTGATTTAGAGGAGTTAGTCTTGAAGTCATGCGCCGGTTAAGGCTAAA
CTGAAAGGACAAGTTTTGGTGACTGCGCTCCTCCAAGCCAGTTACCTCGGTTCAAAGAGTTG
GTAGCTCAGAGAACCTTCGAAAAACCGCCCTGCAAGGCGGTTTTTTCGTTTTCAGAGCAAGA
GATTACGCGCAGACCAAAACGATCTCAAGAAGATCATCTTATTAATCAGATAAAATATTTCT
AGATTTCAGTGCAATTTATCTCTTCAAATGTAGCACCTGAAGTCAGCCCCATACGATATAAGT
TGTAATTCTCATGTTAGTCATGCCCCGCGCCCACCGGAAGGAGCTGACTGGGTTGAAGGCTC
TCAAGGGCATCGGTCGAGATCCCGGTGCCTAATGAGTGAGCTAACTTACATTAATTGCGTTG
CGCTCACTGCCCGCTTTCCAGTCGGGAAACCTGTCGTGCCAGCTGCATTAATGAATCGGCCA

ACGCGCGGGGAGAGGCGGTTTGCGTATTGGGCGCCAGGGTGGTTTTTCTTTTCACCAGTGAG
ACGGGCAACAGCTGATTGCCCTTCACCGCCTGGCCCTGAGAGAGTTGCAGCAAGCGGTCCAC
GCTGGTTTGCCCCAGCAGGCGAAAATCCTGTTTGATGGTGGTTAACGGCGGGATATAACATG
AGCTGTCTTCGGTATCGTCGTATCCCACTACCGAGATGTCCGCACCAACGCGCAGCCCGGAC
TCGGTAATGGCGCGCATTGCGCCCAGCGCCATCTGATCGTTGGCAACCAGCATCGCAGTGGG
AACGATGCCCTCATTCAGCATTTGCATGGTTTGTTGAAAACCGGACATGGCACTCCAGTCGCC
TTCCCGTTCCGCTATCGGCTGAATTTGATTGCGAGTGAGATATTTATGCCAGCCAGCCAGACG
CAGACGCGCCGAGACAGAACTTAATGGGCCCGCTAACAGCGCGATTTGCTGGTGACCCAATG
CGACCAGATGCTCCACGCCCAGTCGCGTACCGTCTTCATGGGAGAAAATAATACTGTTGATG
GGTGTCTGGTCAGAGACATCAAGAAATAACGCCGGAACATTAGTGCAGGCAGCTTCCACAGC
AATGGCATCCTGGTCATCCAGCGGATAGTTAATGATCAGCCCACTGACGCGTTGCGCGAGAA
GATTGTGCACCGCCGCTTTACAGGCTTCGACGCCGCTTCGTTCTACCATCGACACCACCACGC
TGGCACCCAGTTGATCGGCGCGAGATTTAATCGCCGCGACAATTTGCGACGGCGCGTGCAGG
GCCAGACTGGAGGTGGCAACGCCAATCAGCAACGACTGTTTGCCCGCCAGTTGTTGTGCCAC
GCGGTTGGGAATGTAATTCAGCTCCGCCATCGCCGCTTCCACTTTTTCCCGCGTTTTCGCAGA
AACGTGGCTGGCCTGGTTCACCACGCGGGAAACGGTCTGATAAGAGACACCGGCATACTCTG
CGACATCGTATAACGTTACTGGTTTCACATTCACCACCCTGAATTGACTCTCTTCCGGGCGCT
ATCATGCCATACCGCGAAAGGTTTTGCGCCATTCGATGGTGTCCGGGATCTCGACGCTCTCCC
TTATGCGACTCCTGCATTAGGAAATTAATACGACTCACTATA

f. pDuet::GroL

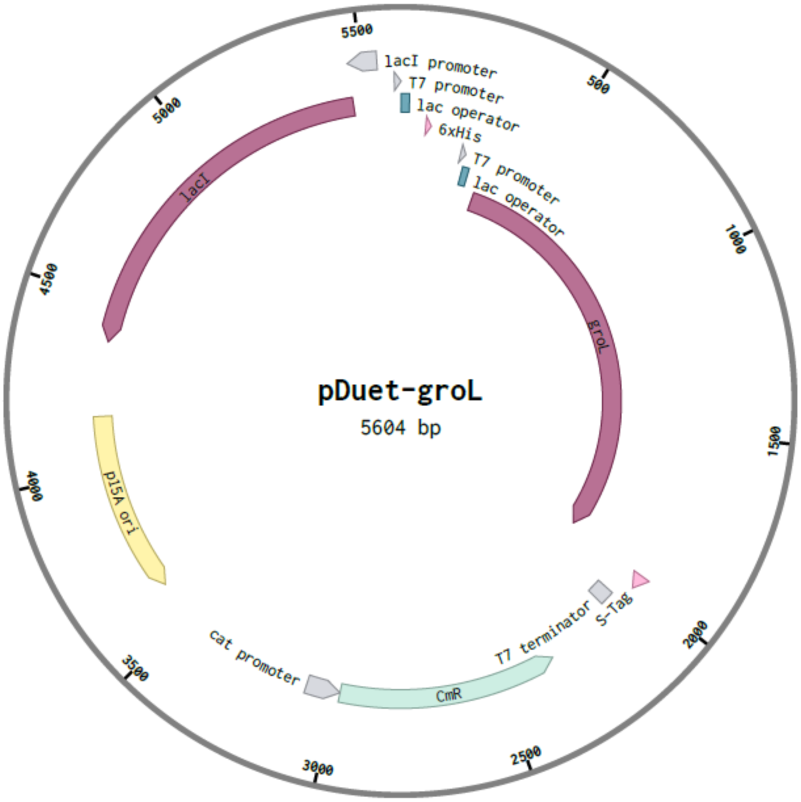

GGGGAATTGTGAGCGGATAACAATTCCCCTGTAGAAATAATTTTGTTTAACTTTAATAAGGA
GATATACCATGGGCAGCAGCCATCACCATCATCACCACAGCCAGGATCCGAATTCGAGCTCG
GCGCGCCTGCAGGTCGACAAGCTTGCGGCCGCATAATGCTTAAGTCGAACAGAAAGTAATCG
TATTGTACACGGCCGCATAATCGAAATTAATACGACTCACTATAGGGGAATTGTGAGCGGAT
AACAATTCCCCATCTTAGTATATTAGTTAAGTATAAGAAGGAGATATACATATGATGGCAGC
TAAAGACGTAAAATTCGGTAACGACGCTCGTGTGAAAATGCTGCGCGGCGTAAACGTACTGG
CAGATGCAGTGAAAGTTACCCTCGGTCCAAAAGGCCGTAACGTAGTTCTGGATAAATCTTTC
GGTGCACCGACCATCACCAAAGATGGTGTTTCCGTTGCTCGTGAAATCGAACTGGAAGACAA
GTTCGAAAATATGGGTGCGCAGATGGTGAAAGAAGTTGCCTCTAAAGCAAACGACGCTGCA
GGCGACGGTACCACCACTGCAACCGTACTGGCTCAGGCTATCATCACTGAAGGTCTGAAAGC
TGTTGCTGCGGGCATGAACCCGATGGACCTGAAACGTGGTATCGACAAAGCGGTTACCGCTG
CAGTTGAAGAACTGAAAGCGCTGTCCGTACCATGCTCTGACTCTAAAGCGATTGCTCAGGTT
GGTACCATCTCCGCTAACTCCGACGAAACCGTAGGTAAACTGATCGCTGAAGCGATGGACAA
AGTCGGTAAAGAAGGCGTTATCACCGTTGAAGACGGTACCGGTCTGCAGGACGAACTGGAC
GTGGTTGAAGGTATGCAGTTCGACCGTGGCTACCTGTCTCCTTACTTCATCAACAAGCCGGAA
ACTGGCGCAGTAGAACTGGAAAGCCCGTTCATCCTGCTGGCTGACAAGAAAATCTCCAACAT

CCGCGAAATGCTGCCGGTTCTGGAAGCTGTTGCCAAAGCAGGCAAACCGCTGCTGATCATCG
CTGAAGATGTAGAAGGCGAAGCGCTGGCAACTCTGGTTGTTAACACCATGCGTGGCATCGTG
AAAGTCGCTGCGGTTAAAGCACCGGGCTTCGGCGATCGTCGTAAAGCTATGCTGCAGGATAT
CGCAACCCTGACTGGCGGTACCGTGATCTCTGAAGAGATCGGTATGGAGCTGGAAAAAGCA
ACCCTGGAAGACCTGGGTCAGGCTAAACGTGTTGTGATCAACAAAGACACCACCACTATCAT
CGATGGCGTGGGTGAAGAAGCTGCAATCCAGGGCCGTGTTGCTCAGATCCGTCAGCAGATTG
AAGAAGCAACTTCTGACTACGACCGTGAAAAACTGCAGGAACGCGTAGCGAAACTGGCAGG
CGGCGTTGCAGTTATCAAAGTGGGTGCTGCTACCGAAGTTGAAATGAAAGAGAAAAAAGCA
CGCGTTGAAGATGCCCTGCACGCGACCCGTGCTGCGGTAGAAGAAGGCGTGGTTGCTGGTGG
TGGTGTTGCGCTGATCCGCGTAGCGTCTAAACTGGCTGACCTGCGTGGTCAGAACGAAGACC
AGAACGTGGGTATCAAAGTTGCACTGCGTGCAATGGAAGCTCCGCTGCGTCAGATCGTATTG
AACTGCGGCGAAGAACCGTCTGTTGTTGCTAACACCGTTAAAGGCGGCGACGGCAACTACGG
TTACAACGCAGCAACCGAAGAATACGGCAACATGATCGACATGGGTATCCTGGATCCAACCA
AAGTAACTCGTTCTGCTCTGCAGTACGCAGCTTCTGTGGCTGGCCTGATGATCACCACCGAAT
GCATGGTTACCGACCTGCCGAAAAACGATGCAGCTGACTTAGGCGCTGCTGGCGGTATGGGC
GGCATGGGTGGCATGGGCGGCATGATGTAACTCGAGTCTGGTAAAGAAACCGCTGCTGCGA
AATTTGAACGCCAGCACATGGACTCGTCTACTAGCGCAGCTTAATTAACCTAGGCTGCTGCC
ACCGCTGAGCAATAACTAGCATAACCCCTTGGGGCCTCTAAACGGGTCTTGAGGGGTTTTTT
GCTGAAACCTCAGGCATTTGAGAAGCACACGGTCACACTGCTTCCGGTAGTCAATAAACCGG
TAAACCAGCAATAGACATAAGCGGCTATTTAACGACCCTGCCCTGAACCGACGACCGGGTCG
AATTTGCTTTCGAATTTCTGCCATTCATCCGCTTATTATCACTTATTCAGGCGTAGCACCAGGC
GTTTAAGGGCACCAATAACTGCCTTAAAAAAATTACGCCCCGCCCTGCCACTCATCGCAGTA
CTGTTGTAATTCATTAAGCATTCTGCCGACATGGAAGCCATCACAGACGGCATGATGAACCT
GAATCGCCAGCGGCATCAGCACCTTGTCGCCTTGCGTATAATATTTGCCCATAGTGAAAACG
GGGGCGAAGAAGTTGTCCATATTGGCCACGTTTAAATCAAAACTGGTGAAACTCACCCAGGG
ATTGGCTGAGACGAAAAACATATTCTCAATAAACCCTTTAGGGAAATAGGCCAGGTTTTCAC
CGTAACACGCCACATCTTGCGAATATATGTGTAGAAACTGCCGGAAATCGTCGTGGTATTCA
CTCCAGAGCGATGAAAACGTTTCAGTTTGCTCATGGAAAACGGTGTAACAAGGGTGAACACT
ATCCCATATCACCAGCTCACCGTCTTTCATTGCCATACGGAACTCCGGATGAGCATTCATCAG
GCGGGCAAGAATGTGAATAAAGGCCGGATAAAACTTGTGCTTATTTTTCTTTACGGTCTTTAA
AAAGGCCGTAATATCCAGCTGAACGGTCTGGTTATAGGTACATTGAGCAACTGACTGAAATG
CCTCAAAATGTTCTTTACGATGCCATTGGGATATATCAACGGTGGTATATCCAGTGATTTTTT
TCTCCATTTTAGCTTCCTTAGCTCCTGAAAATCTCGATAACTCAAAAAATACGCCCGGTAGTG
ATCTTATTTCATTATGGTGAAAGTTGGAACCTCTTACGTGCCGATCAACGTCTCATTTTCGCC

AAAAGTTGGCCCAGGGCTTCCCGGTATCAACAGGGACACCAGGATTTATTTATTCTGCGAAG
TGATCTTCCGTCACAGGTATTTATTCGGCGCAAAGTGCGTCGGGTGATGCTGCCAACTTACTG
ATTTAGTGTATGATGGTGTTTTTGAGGTGCTCCAGTGGCTTCTGTTTCTATCAGCTGTCCCTCC
TGTTCAGCTACTGACGGGGTGGTGCGTAACGGCAAAAGCACCGCCGGACATCAGCGCTAGCG
GAGTGTATACTGGCTTACTATGTTGGCACTGATGAGGGTGTCAGTGAAGTGCTTCATGTGGC
AGGAGAAAAAAGGCTGCACCGGTGCGTCAGCAGAATATGTGATACAGGATATATTCCGCTTC
CTCGCTCACTGACTCGCTACGCTCGGTCGTTCGACTGCGGCGAGCGGAAATGGCTTACGAAC
GGGGCGGAGATTTCCTGGAAGATGCCAGGAAGATACTTAACAGGGAAGTGAGAGGGCCGCG
GCAAAGCCGTTTTTCCATAGGCTCCGCCCCCCTGACAAGCATCACGAAATCTGACGCTCAAA
TCAGTGGTGGCGAAACCCGACAGGACTATAAAGATACCAGGCGTTTCCCCTGGCGGCTCCCT
CGTGCGCTCTCCTGTTCCTGCCTTTCGGTTTACCGGTGTCATTCCGCTGTTATGGCCGCGTTTG
TCTCATTCCACGCCTGACACTCAGTTCCGGGTAGGCAGTTCGCTCCAAGCTGGACTGTATGCA
CGAACCCCCCGTTCAGTCCGACCGCTGCGCCTTATCCGGTAACTATCGTCTTGAGTCCAACCC
GGAAAGACATGCAAAAGCACCACTGGCAGCAGCCACTGGTAATTGATTTAGAGGAGTTAGT
CTTGAAGTCATGCGCCGGTTAAGGCTAAACTGAAAGGACAAGTTTTGGTGACTGCGCTCCTC
CAAGCCAGTTACCTCGGTTCAAAGAGTTGGTAGCTCAGAGAACCTTCGAAAAACCGCCCTGC
AAGGCGGTTTTTTCGTTTTCAGAGCAAGAGATTACGCGCAGACCAAAACGATCTCAAGAAGA
TCATCTTATTAATCAGATAAAATATTTCTAGATTTCAGTGCAATTTATCTCTTCAAATGTAGC
ACCTGAAGTCAGCCCCATACGATATAAGTTGTAATTCTCATGTTAGTCATGCCCCGCGCCCAC
CGGAAGGAGCTGACTGGGTTGAAGGCTCTCAAGGGCATCGGTCGAGATCCCGGTGCCTAATG
AGTGAGCTAACTTACATTAATTGCGTTGCGCTCACTGCCCGCTTTCCAGTCGGGAAACCTGTC
GTGCCAGCTGCATTAATGAATCGGCCAACGCGCGGGGAGAGGCGGTTTGCGTATTGGGCGCC
AGGGTGGTTTTTCTTTTCACCAGTGAGACGGGCAACAGCTGATTGCCCTTCACCGCCTGGCCC
TGAGAGAGTTGCAGCAAGCGGTCCACGCTGGTTTGCCCCAGCAGGCGAAAATCCTGTTTGAT
GGTGGTTAACGGCGGGATATAACATGAGCTGTCTTCGGTATCGTCGTATCCCACTACCGAGA
TGTCCGCACCAACGCGCAGCCCGGACTCGGTAATGGCGCGCATTGCGCCCAGCGCCATCTGA
TCGTTGGCAACCAGCATCGCAGTGGGAACGATGCCCTCATTCAGCATTTGCATGGTTTGTTGA
AAACCGGACATGGCACTCCAGTCGCCTTCCCGTTCCGCTATCGGCTGAATTTGATTGCGAGTG
AGATATTTATGCCAGCCAGCCAGACGCAGACGCGCCGAGACAGAACTTAATGGGCCCGCTA
ACAGCGCGATTTGCTGGTGACCCAATGCGACCAGATGCTCCACGCCCAGTCGCGTACCGTCT
TCATGGGAGAAAATAATACTGTTGATGGGTGTCTGGTCAGAGACATCAAGAAATAACGCCGG
AACATTAGTGCAGGCAGCTTCCACAGCAATGGCATCCTGGTCATCCAGCGGATAGTTAATGA
TCAGCCCACTGACGCGTTGCGCGAGAAGATTGTGCACCGCCGCTTTACAGGCTTCGACGCCG
CTTCGTTCTACCATCGACACCACCACGCTGGCACCCAGTTGATCGGCGCGAGATTTAATCGCC

GCGACAATTTGCGACGGCGCGTGCAGGGCCAGACTGGAGGTGGCAACGCCAATCAGCAACG
ACTGTTTGCCCGCCAGTTGTTGTGCCACGCGGTTGGGAATGTAATTCAGCTCCGCCATCGCCG
CTTCCACTTTTTCCCGCGTTTTCGCAGAAACGTGGCTGGCCTGGTTCACCACGCGGGAAACGG
TCTGATAAGAGACACCGGCATACTCTGCGACATCGTATAACGTTACTGGTTTCACATTCACCA
CCCTGAATTGACTCTCTTCCGGGCGCTATCATGCCATACCGCGAAAGGTTTTGCGCCATTCGA
TGGTGTCCGGGATCTCGACGCTCTCCCTTATGCGACTCCTGCATTAGGAAATTAATACGACTC
ACTATA

g. pDuet::GroSL

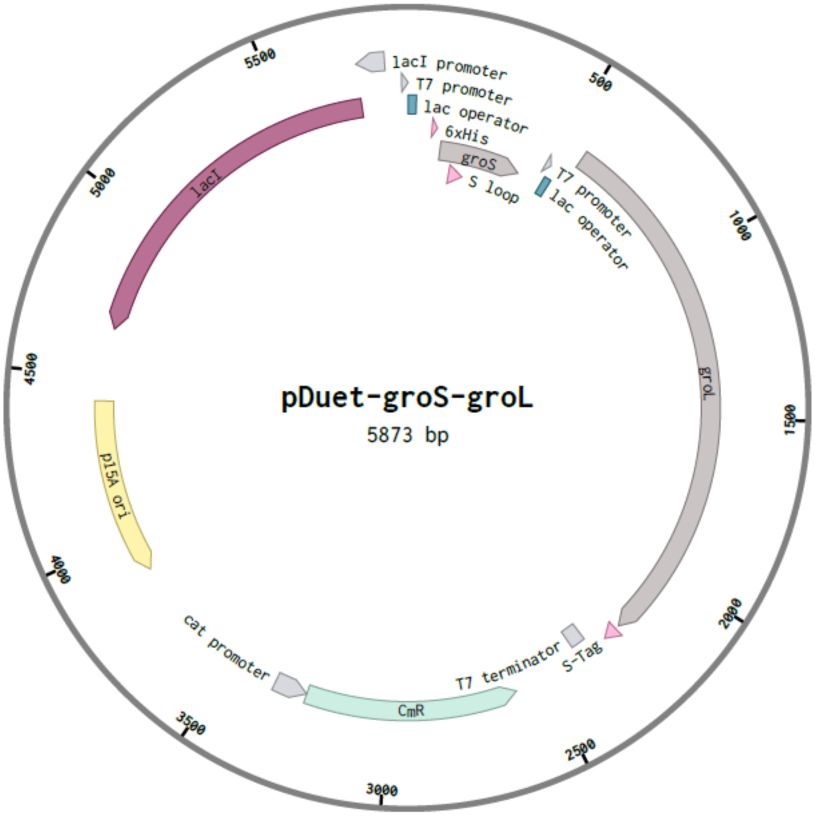

GGGGAATTGTGAGCGGATAACAATTCCCCTGTAGAAATAATTTTGTTTAACTTTAATAAGGA
GATATACCATGGGCAGCAGCCATCACCATCATCACCACAGCCAGGATCCGAATTCATGAATA
TTCGTCCATTGCATGATCGCGTGATCGTCAAGCGTAAAGAAGTTGAAACTAAATCTGCTGGC
GGCATCGTTCTGACCGGCTCTGCAGCGGCTAAATCCACCCGCGGCGAAGTGCTGGCTGTCGG
CAATGGCCGTATCCTTGAAAATGGCGAAGTGAAGCCGCTGGATGTGAAAGTTGGCGACATCG
TTATTTTCAACGATGGCTACGGTGTGAAATCTGAGAAGATCGACAATGAAGAAGTGTTGATC
ATGTCCGAAAGCGACATTCTGGCAATTGTTGAAGCGTAAAAGCTTGCGGCCGCATAATGCTT
AAGTCGAACAGAAAGTAATCGTATTGTACACGGCCGCATAATCGAAATTAATACGACTCACT
ATAGGGGAATTGTGAGCGGATAACAATTCCCCATCTTAGTATATTAGTTAAGTATAAGAAGG
AGATATACATATGATGGCAGCTAAAGACGTAAAATTCGGTAACGACGCTCGTGTGAAAATGC
TGCGCGGCGTAAACGTACTGGCAGATGCAGTGAAAGTTACCCTCGGTCCAAAAGGCCGTAAC
GTAGTTCTGGATAAATCTTTCGGTGCACCGACCATCACCAAAGATGGTGTTTCCGTTGCTCGT
GAAATCGAACTGGAAGACAAGTTCGAAAATATGGGTGCGCAGATGGTGAAAGAAGTTGCCT
CTAAAGCAAACGACGCTGCAGGCGACGGTACCACCACTGCAACCGTACTGGCTCAGGCTATC
ATCACTGAAGGTCTGAAAGCTGTTGCTGCGGGCATGAACCCGATGGACCTGAAACGTGGTAT
CGACAAAGCGGTTACCGCTGCAGTTGAAGAACTGAAAGCGCTGTCCGTACCATGCTCTGACT

CTAAAGCGATTGCTCAGGTTGGTACCATCTCCGCTAACTCCGACGAAACCGTAGGTAAACTG
ATCGCTGAAGCGATGGACAAAGTCGGTAAAGAAGGCGTTATCACCGTTGAAGACGGTACCG
GTCTGCAGGACGAACTGGACGTGGTTGAAGGTATGCAGTTCGACCGTGGCTACCTGTCTCCT
TACTTCATCAACAAGCCGGAAACTGGCGCAGTAGAACTGGAAAGCCCGTTCATCCTGCTGGC
TGACAAGAAAATCTCCAACATCCGCGAAATGCTGCCGGTTCTGGAAGCTGTTGCCAAAGCAG
GCAAACCGCTGCTGATCATCGCTGAAGATGTAGAAGGCGAAGCGCTGGCAACTCTGGTTGTT
AACACCATGCGTGGCATCGTGAAAGTCGCTGCGGTTAAAGCACCGGGCTTCGGCGATCGTCG
TAAAGCTATGCTGCAGGATATCGCAACCCTGACTGGCGGTACCGTGATCTCTGAAGAGATCG
GTATGGAGCTGGAAAAAGCAACCCTGGAAGACCTGGGTCAGGCTAAACGTGTTGTGATCAA
CAAAGACACCACCACTATCATCGATGGCGTGGGTGAAGAAGCTGCAATCCAGGGCCGTGTTG
CTCAGATCCGTCAGCAGATTGAAGAAGCAACTTCTGACTACGACCGTGAAAAACTGCAGGAA
CGCGTAGCGAAACTGGCAGGCGGCGTTGCAGTTATCAAAGTGGGTGCTGCTACCGAAGTTGA
AATGAAAGAGAAAAAAGCACGCGTTGAAGATGCCCTGCACGCGACCCGTGCTGCGGTAGAA
GAAGGCGTGGTTGCTGGTGGTGGTGTTGCGCTGATCCGCGTAGCGTCTAAACTGGCTGACCT
GCGTGGTCAGAACGAAGACCAGAACGTGGGTATCAAAGTTGCACTGCGTGCAATGGAAGCT
CCGCTGCGTCAGATCGTATTGAACTGCGGCGAAGAACCGTCTGTTGTTGCTAACACCGTTAA
AGGCGGCGACGGCAACTACGGTTACAACGCAGCAACCGAAGAATACGGCAACATGATCGAC
ATGGGTATCCTGGATCCAACCAAAGTAACTCGTTCTGCTCTGCAGTACGCAGCTTCTGTGGCT
GGCCTGATGATCACCACCGAATGCATGGTTACCGACCTGCCGAAAAACGATGCAGCTGACTT
AGGCGCTGCTGGCGGTATGGGCGGCATGGGTGGCATGGGCGGCATGATGTAACTCGAGTCTG
GTAAAGAAACCGCTGCTGCGAAATTTGAACGCCAGCACATGGACTCGTCTACTAGCGCAGCT
TAATTAACCTAGGCTGCTGCCACCGCTGAGCAATAACTAGCATAACCCCTTGGGGCCTCTAA
ACGGGTCTTGAGGGGTTTTTTGCTGAAACCTCAGGCATTTGAGAAGCACACGGTCACACTGC
TTCCGGTAGTCAATAAACCGGTAAACCAGCAATAGACATAAGCGGCTATTTAACGACCCTGC
CCTGAACCGACGACCGGGTCGAATTTGCTTTCGAATTTCTGCCATTCATCCGCTTATTATCAC
TTATTCAGGCGTAGCACCAGGCGTTTAAGGGCACCAATAACTGCCTTAAAAAATTACGCCC
CGCCCTGCCACTCATCGCAGTACTGTTGTAATTCATTAAGCATTCTGCCGACATGGAAGCCAT
CACAGACGGCATGATGAACCTGAATCGCCAGCGGCATCAGCACCTTGTCGCCTTGCGTATAA
TATTTGCCCATAGTGAAAACGGGGGCGAAGAAGTTGTCCATATTGGCCACGTTTAAATCAAA
ACTGGTGAAACTCACCCAGGGATTGGCTGAGACGAAAAACATATTCTCAATAAACCCTTTAG
GGAAATAGGCCAGGTTTTCACCGTAACACGCCACATCTTGCGAATATATGTGTAGAAACTGC
CGGAAATCGTCGTGGTATTCACTCCAGAGCGATGAAAACGTTTCAGTTTGCTCATGGAAAAC
GGTGTAACAAGGGTGAACACTATCCCATATCACCAGCTCACCGTCTTTCATTGCCATACGGA
ACTCCGGATGAGCATTCATCAGGCGGGCAAGAATGTGAATAAAGGCCGGATAAAACTTGTG

CTTATTTTTCTTTACGGTCTTTAAAAAGGCCGTAATATCCAGCTGAACGGTCTGGTTATAGGT
ACATTGAGCAACTGACTGAAATGCCTCAAAATGTTCTTTACGATGCCATTGGGATATATCAA
CGGTGGTATATCCAGTGATTTTTTTCTCCATTTTAGCTTCCTTAGCTCCTGAAAATCTCGATAA
CTCAAAAAATACGCCCGGTAGTGATCTTATTTCATTATGGTGAAAGTTGGAACCTCTTACGTG
CCGATCAACGTCTCATTTTCGCCAAAAGTTGGCCCAGGGCTTCCCGGTATCAACAGGGACAC
CAGGATTTATTTATTCTGCGAAGTGATCTTCCGTCACAGGTATTTATTCGGCGCAAAGTGCGT
CGGGTGATGCTGCCAACTTACTGATTTAGTGTATGATGGTGTTTTTGAGGTGCTCCAGTGGCT
TCTGTTTCTATCAGCTGTCCCTCCTGTTCAGCTACTGACGGGGTGGTGCGTAACGGCAAAAGC
ACCGCCGGACATCAGCGCTAGCGGAGTGTATACTGGCTTACTATGTTGGCACTGATGAGGGT
GTCAGTGAAGTGCTTCATGTGGCAGGAGAAAAAAGGCTGCACCGGTGCGTCAGCAGAATAT
GTGATACAGGATATATTCCGCTTCCTCGCTCACTGACTCGCTACGCTCGGTCGTTCGACTGCG
GCGAGCGGAAATGGCTTACGAACGGGGCGGAGATTTCCTGGAAGATGCCAGGAAGATACTT
AACAGGGAAGTGAGAGGGCCGCGGCAAAGCCGTTTTTCCATAGGCTCCGCCCCCCTGACAAG
CATCACGAAATCTGACGCTCAAATCAGTGGTGGCGAAACCCGACAGGACTATAAAGATACC
AGGCGTTTCCCCTGGCGGCTCCCTCGTGCGCTCTCCTGTTCCTGCCTTTCGGTTTACCGGTGTC
ATTCCGCTGTTATGGCCGCGTTTGTCTCATTCCACGCCTGACACTCAGTTCCGGGTAGGCAGT
TCGCTCCAAGCTGGACTGTATGCACGAACCCCCCGTTCAGTCCGACCGCTGCGCCTTATCCGG
TAACTATCGTCTTGAGTCCAACCCGGAAAGACATGCAAAAGCACCACTGGCAGCAGCCACTG
GTAATTGATTTAGAGGAGTTAGTCTTGAAGTCATGCGCCGGTTAAGGCTAAACTGAAAGGAC
AAGTTTTGGTGACTGCGCTCCTCCAAGCCAGTTACCTCGGTTCAAAGAGTTGGTAGCTCAGA
GAACCTTCGAAAAACCGCCCTGCAAGGCGGTTTTTTCGTTTTCAGAGCAAGAGATTACGCGC
AGACCAAAACGATCTCAAGAAGATCATCTTATTAATCAGATAAAATATTTCTAGATTTCAGT
GCAATTTATCTCTTCAAATGTAGCACCTGAAGTCAGCCCCATACGATATAAGTTGTAATTCTC
ATGTTAGTCATGCCCCGCGCCCACCGGAAGGAGCTGACTGGGTTGAAGGCTCTCAAGGGCAT
CGGTCGAGATCCCGGTGCCTAATGAGTGAGCTAACTTACATTAATTGCGTTGCGCTCACTGCC
CGCTTTCCAGTCGGGAAACCTGTCGTGCCAGCTGCATTAATGAATCGGCCAACGCGCGGGGA
GAGGCGGTTTGCGTATTGGGCGCCAGGGTGGTTTTTCTTTTCACCAGTGAGACGGGCAACAG
CTGATTGCCCTTCACCGCCTGGCCCTGAGAGAGTTGCAGCAAGCGGTCCACGCTGGTTTGCCC
CAGCAGGCGAAAATCCTGTTTGATGGTGGTTAACGGCGGGATATAACATGAGCTGTCTTCGG
TATCGTCGTATCCCACTACCGAGATGTCCGCACCAACGCGCAGCCCGGACTCGGTAATGGCG
CGCATTGCGCCCAGCGCCATCTGATCGTTGGCAACCAGCATCGCAGTGGGAACGATGCCCTC
ATTCAGCATTTGCATGGTTTGTTGAAAACCGGACATGGCACTCCAGTCGCCTTCCCGTTCCGC
TATCGGCTGAATTTGATTGCGAGTGAGATATTTATGCCAGCCAGCCAGACGCAGACGCGCCG
AGACAGAACTTAATGGGCCCGCTAACAGCGCGATTTGCTGGTGACCCAATGCGACCAGATGC

TCCACGCCCAGTCGCGTACCGTCTTCATGGGAGAAAATAATACTGTTGATGGGTGTCTGGTC
AGAGACATCAAGAAATAACGCCGGAACATTAGTGCAGGCAGCTTCCACAGCAATGGCATCC
TGGTCATCCAGCGGATAGTTAATGATCAGCCCACTGACGCGTTGCGCGAGAAGATTGTGCAC
CGCCGCTTTACAGGCTTCGACGCCGCTTCGTTCTACCATCGACACCACCACGCTGGCACCCAG
TTGATCGGCGCGAGATTTAATCGCCGCGACAATTTGCGACGGCGCGTGCAGGGCCAGACTGG
AGGTGGCAACGCCAATCAGCAACGACTGTTTGCCCGCCAGTTGTTGTGCCACGCGGTTGGGA
ATGTAATTCAGCTCCGCCATCGCCGCTTCCACTTTTTCCCGCGTTTTCGCAGAAACGTGGCTG
GCCTGGTTCACCACGCGGGAAACGGTCTGATAAGAGACACCGGCATACTCTGCGACATCGTA
TAACGTTACTGGTTTCACATTCACCACCCTGAATTGACTCTCTTCCGGGCGCTATCATGCCAT
ACCGCGAAAGGTTTTGCGCCATTCGATGGTGTCCGGGATCTCGACGCTCTCCCTTATGCGACT
CCTGCATTAGGAAATTAATACGACTCACTATA

h. pDuet::DnaK

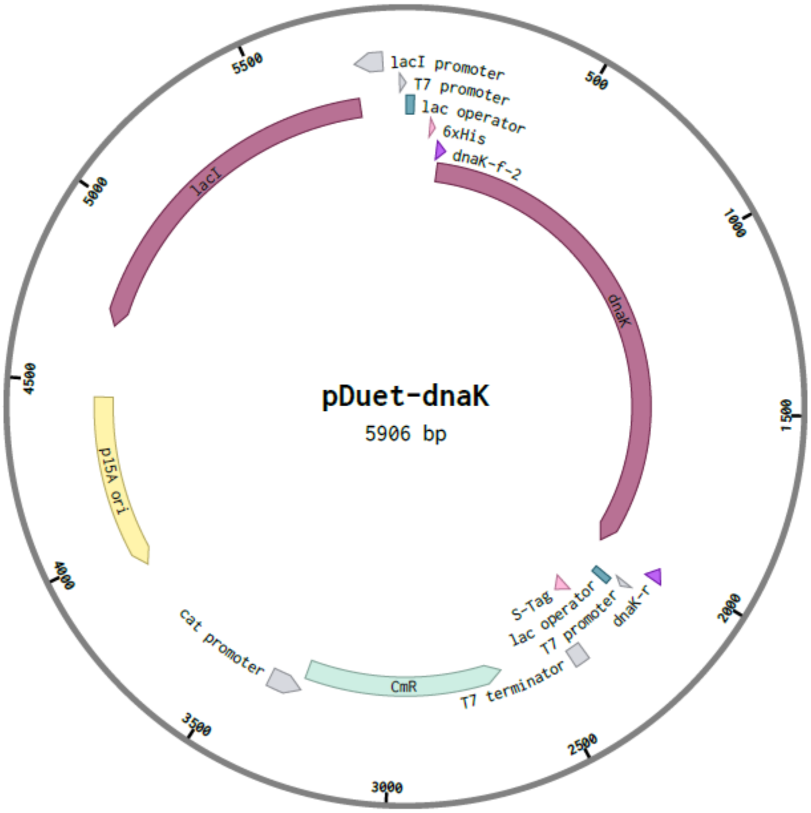

GGGGAATTGTGAGCGGATAACAATTCCCCTGTAGAAATAATTTTGTTTAACTTTAATAAGGA
GATATACCATGGGCAGCAGCCATCACCATCATCACCACAGCCAGGATCCGAATTCGAGCTCA
TGGGTAAAATAATTGGTATCGACCTGGGTACTACCAACTCTTGTGTAGCGATTATGGATGGC
ACCACTCCTCGCGTGCTGGAGAACGCCGAAGGCGATCGCACCACGCCTTCTATCATTGCCTA
TACCCAGGATGGTGAAACTCTAGTTGGTCAGCCGGCTAAACGTCAGGCAGTGACGAACCCGC
AAAACACTCTGTTTGCGATTAAACGCCTGATTGGTCGCCGCTTCCAGGACGAAGAAGTACAG
CGTGATGTTTCCATCATGCCGTTCAAAATTATTGCTGCTGATAACGGCGACGCATGGGTCGAA
GTTAAAGGCCAGAAAATGGCACCGCCGCAGATTTCTGCTGAAGTGCTGAAAAAAATGAAGA
AAACCGCTGAAGATTACCTGGGTGAACCGGTAACTGAAGCTGTTATCACCGTACCGGCATAC
TTTAACGATGCTCAGCGTCAGGCAACCAAAGACGCAGGCCGTATCGCTGGTCTGGAAGTAAA
ACGTATCATCAACGAACCGACCGCAGCTGCGCTGGCTTACGGTCTGGACAAAGGCACTGGCA
ACCGTACTATCGCGGTTTATGACCTGGGTGGTGGTACTTTCGATATTTCTATTATCGAAATCG
ACGAAGTTGACGGCGAAAAAACCTTCGAAGTTCTGGCAACCAACGGTGATACCCACCTGGG
GGGTGAAGACTTCGACAGCCGTCTGATCAACTATCTGGTTGAAGAATTCAAGAAAGATCAGG
GCATTGACCTGCGCAACGATCCGCTGGCAATGCAGCGCCTGAAAGAAGCGGCAGAAAAAGC
GAAAATCGAACTGTCTTCCGCTCAGCAGACCGACGTTAACCTGCCATACATCACTGCAGACG

CGACCGGTCCGAAACACATGAACATCAAAGTGACTCGTGCGAAACTGGAAAGCCTGGTTGA
AGATCTGGTAAACCGTTCCATTGAGCCGCTGAAAGTTGCACTGCAGGACGCTGGCCTGTCCG
TATCTGATATCGACGACGTTATCCTCGTTGGTGGTCAGACTCGTATGCCAATGGTTCAGAAGA
AAGTTGCTGAGTTCTTTGGTAAAGAGCCGCGTAAAGACGTTAACCCGGACGAAGCTGTAGCA
ATCGGTGCTGCTGTTCAGGGTGGTGTTCTGACTGGTGACGTAAAAGACGTACTGCTGCTGGA
CGTTACCCCGCTGTCTCTGGGTATCGAAACCATGGGCGGTGTGATGACGACGCTGATCGCGA
AAAACACCACTATCCCGACCAAGCACAGCCAGGTGTTCTCTACCGCTGAAGACAACCAGTCT
GCGGTAACCATCCATGTGCTGCAGGGTGAACGTAAACGTGCGGCTGATAACAAATCTCTGGG
TCAGTTCAACCTAGATGGTATCAACCCGGCACCGCGCGGCATGCCGCAGATCGAAGTTACCT
TCGATATCGATGCTGACGGTATCCTGCACGTTTCCGCGAAAGATAAAAACAGCGGTAAAGAG
CAGAAGATCACCATCAAGGCTTCTTCTGGTCTGAACGAAGATGAAATCCAGAAAATGGTACG
CGACGCAGAAGCTAACGCCGAAGCTGACCGTAAGTTTGAAGAGCTGGTACAGACTCGCAAC
CAGGGCGACCATCTGCTGCACAGCACCCGTAAGCAGGTTGAAGAAGCAGGCGACAAACTGC
CGGCTGACGACAAAACTGCTATCGAGTCTGCGCTGACTGCACTGGAAACTGCTCTGAAAGGT
GAAGACAAAGCCGCTATCGAAGCGAAAATGCAGGAACTGGCACAGGTTTCCCAGAAACTGA
TGGAAATCGCCCAGCAGCAACATGCCCAGCAGCAGACTGCCGGTGCTGATGCTTCTGCAAAC
AACGCGAAAGATGACGATGTTGTCGACGCTGAATTTGAAGAAGTCAAAGACAAAAAATAAA
AGCTTGCGGCCGCATAATGCTTAAGTCGAACAGAAAGTAATCGTATTGTACACGGCCGCATA
ATCGAAATTAATACGACTCACTATAGGGGAATTGTGAGCGGATAACAATTCCCCATCTTAGT
ATATTAGTTAAGTATAAGAAGGAGATATACATATGGCAGATCTCAATTGGATATCGGCCGGC
CACGCGATCGCTGACGTCGGTACCCTCGAGTCTGGTAAAGAAACCGCTGCTGCGAAATTTGA
ACGCCAGCACATGGACTCGTCTACTAGCGCAGCTTAATTAACCTAGGCTGCTGCCACCGCTG
AGCAATAACTAGCATAACCCCTTGGGGCCTCTAAACGGGTCTTGAGGGGTTTTTTGCTGAAA
CCTCAGGCATTTGAGAAGCACACGGTCACACTGCTTCCGGTAGTCAATAAACCGGTAAACCA
GCAATAGACATAAGCGGCTATTTAACGACCCTGCCCTGAACCGACGACCGGGTCGAATTTGC
TTTCGAATTTCTGCCATTCATCCGCTTATTATCACTTATTCAGGCGTAGCACCAGGCGTTTAA
GGGCACCAATAACTGCCTTAAAAAAATTACGCCCCGCCCTGCCACTCATCGCAGTACTGTTG
TAATTCATTAAGCATTCTGCCGACATGGAAGCCATCACAGACGGCATGATGAACCTGAATCG
CCAGCGGCATCAGCACCTTGTCGCCTTGCGTATAATATTTGCCCATAGTGAAAACGGGGGCG
AAGAAGTTGTCCATATTGGCCACGTTTAAATCAAAACTGGTGAAACTCACCCAGGGATTGGC
TGAGACGAAAAACATATTCTCAATAAACCCTTTAGGGAAATAGGCCAGGTTTTCACCGTAAC
ACGCCACATCTTGCGAATATATGTGTAGAAACTGCCGGAAATCGTCGTGGTATTCACTCCAG
AGCGATGAAAACGTTTCAGTTTGCTCATGGAAAACGGTGTAACAAGGGTGAACACTATCCCA
TATCACCAGCTCACCGTCTTTCATTGCCATACGGAACTCCGGATGAGCATTCATCAGGCGGGC

AAGAATGTGAATAAAGGCCGGATAAAACTTGTGCTTATTTTTCTTTACGGTCTTTAAAAAGGC
CGTAATATCCAGCTGAACGGTCTGGTTATAGGTACATTGAGCAACTGACTGAAATGCCTCAA
AATGTTCTTTACGATGCCATTGGGATATATCAACGGTGGTATATCCAGTGATTTTTTTCTCCAT
TTTAGCTTCCTTAGCTCCTGAAAATCTCGATAACTCAAAAAATACGCCCGGTAGTGATCTTAT
TTCATTATGGTGAAAGTTGGAACCTCTTACGTGCCGATCAACGTCTCATTTTCGCCAAAAGTT
GGCCCAGGGCTTCCCGGTATCAACAGGGACACCAGGATTTATTTATTCTGCGAAGTGATCTTC
CGTCACAGGTATTTATTCGGCGCAAAGTGCGTCGGGTGATGCTGCCAACTTACTGATTTAGTG
TATGATGGTGTTTTTGAGGTGCTCCAGTGGCTTCTGTTTCTATCAGCTGTCCCTCCTGTTCAGC
TACTGACGGGGTGGTGCGTAACGGCAAAAGCACCGCCGGACATCAGCGCTAGCGGAGTGTA
TACTGGCTTACTATGTTGGCACTGATGAGGGTGTCAGTGAAGTGCTTCATGTGGCAGGAGAA
AAAAGGCTGCACCGGTGCGTCAGCAGAATATGTGATACAGGATATATTCCGCTTCCTCGCTC
ACTGACTCGCTACGCTCGGTCGTTCGACTGCGGCGAGCGGAAATGGCTTACGAACGGGGCGG
AGATTTCCTGGAAGATGCCAGGAAGATACTTAACAGGGAAGTGAGAGGGCCGCGGCAAAGC
CGTTTTTCCATAGGCTCCGCCCCCCTGACAAGCATCACGAAATCTGACGCTCAAATCAGTGGT
GGCGAAACCCGACAGGACTATAAAGATACCAGGCGTTTCCCCTGGCGGCTCCCTCGTGCGCT
CTCCTGTTCCTGCCTTTCGGTTTACCGGTGTCATTCCGCTGTTATGGCCGCGTTTGTCTCATTC
CACGCCTGACACTCAGTTCCGGGTAGGCAGTTCGCTCCAAGCTGGACTGTATGCACGAACCC
CCCGTTCAGTCCGACCGCTGCGCCTTATCCGGTAACTATCGTCTTGAGTCCAACCCGGAAAGA
CATGCAAAAGCACCACTGGCAGCAGCCACTGGTAATTGATTTAGAGGAGTTAGTCTTGAAGT
CATGCGCCGGTTAAGGCTAAACTGAAAGGACAAGTTTTGGTGACTGCGCTCCTCCAAGCCAG
TTACCTCGGTTCAAAGAGTTGGTAGCTCAGAGAACCTTCGAAAAACCGCCCTGCAAGGCGGT
TTTTTCGTTTTCAGAGCAAGAGATTACGCGCAGACCAAAACGATCTCAAGAAGATCATCTTA
TTAATCAGATAAAATATTTCTAGATTTCAGTGCAATTTATCTCTTCAAATGTAGCACCTGAAG
TCAGCCCCATACGATATAAGTTGTAATTCTCATGTTAGTCATGCCCCGCGCCCACCGGAAGG
AGCTGACTGGGTTGAAGGCTCTCAAGGGCATCGGTCGAGATCCCGGTGCCTAATGAGTGAGC
TAACTTACATTAATTGCGTTGCGCTCACTGCCCGCTTTCCAGTCGGGAAACCTGTCGTGCCAG
CTGCATTAATGAATCGGCCAACGCGCGGGGAGAGGCGGTTTGCGTATTGGGCGCCAGGGTGG
TTTTTCTTTTCACCAGTGAGACGGGCAACAGCTGATTGCCCTTCACCGCCTGGCCCTGAGAGA
GTTGCAGCAAGCGGTCCACGCTGGTTTGCCCCAGCAGGCGAAAATCCTGTTTGATGGTGGTT
AACGGCGGGATATAACATGAGCTGTCTTCGGTATCGTCGTATCCCACTACCGAGATGTCCGC
ACCAACGCGCAGCCCGGACTCGGTAATGGCGCGCATTGCGCCCAGCGCCATCTGATCGTTGG
CAACCAGCATCGCAGTGGGAACGATGCCCTCATTCAGCATTTGCATGGTTTGTTGAAAACCG
GACATGGCACTCCAGTCGCCTTCCCGTTCCGCTATCGGCTGAATTTGATTGCGAGTGAGATAT
TTATGCCAGCCAGCCAGACGCAGACGCGCCGAGACAGAACTTAATGGGCCCGCTAACAGCG

CGATTTGCTGGTGACCCAATGCGACCAGATGCTCCACGCCCAGTCGCGTACCGTCTTCATGG
GAGAAAATAATACTGTTGATGGGTGTCTGGTCAGAGACATCAAGAAATAACGCCGGAACATT
AGTGCAGGCAGCTTCCACAGCAATGGCATCCTGGTCATCCAGCGGATAGTTAATGATCAGCC
CACTGACGCGTTGCGCGAGAAGATTGTGCACCGCCGCTTTACAGGCTTCGACGCCGCTTCGTT
CTACCATCGACACCACCACGCTGGCACCCAGTTGATCGGCGCGAGATTTAATCGCCGCGACA
ATTTGCGACGGCGCGTGCAGGGCCAGACTGGAGGTGGCAACGCCAATCAGCAACGACTGTTT
GCCCGCCAGTTGTTGTGCCACGCGGTTGGGAATGTAATTCAGCTCCGCCATCGCCGCTTCCAC
TTTTTCCCGCGTTTTCGCAGAAACGTGGCTGGCCTGGTTCACCACGCGGGAAACGGTCTGATA
AGAGACACCGGCATACTCTGCGACATCGTATAACGTTACTGGTTTCACATTCACCACCCTGA
ATTGACTCTCTTCCGGGCGCTATCATGCCATACCGCGAAAGGTTTTGCGCCATTCGATGGTGT
CCGGGATCTCGACGCTCTCCCTTATGCGACTCCTGCATTAGGAAATTAATACGACTCACTATA

i. pDuet::DnaJ

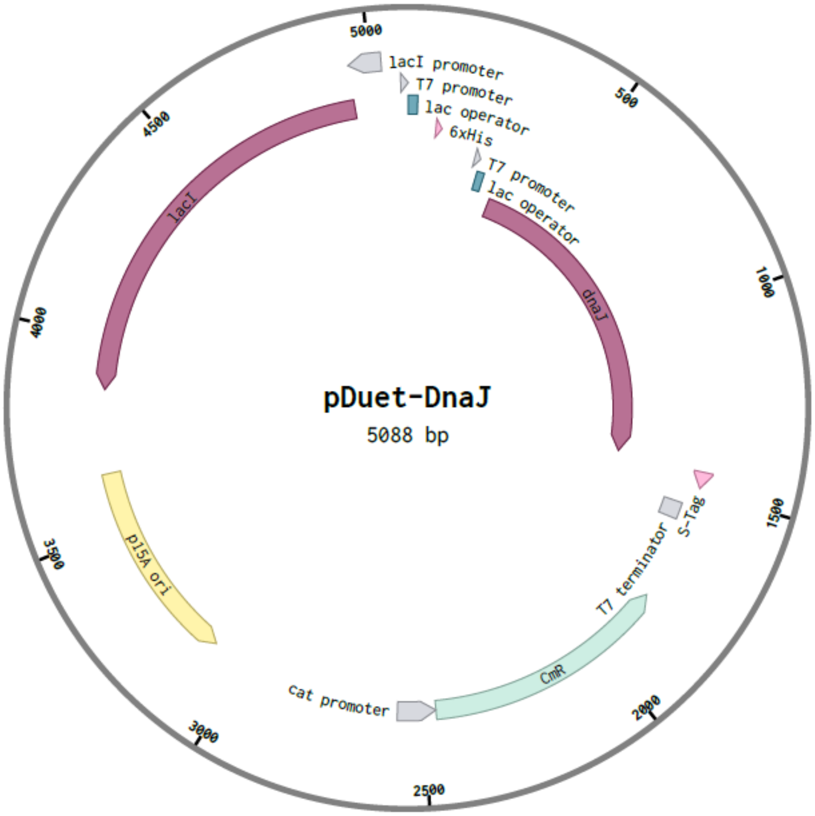

GGGGAATTGTGAGCGGATAACAATTCCCCTGTAGAAATAATTTTGTTTAACTTTAATAAGGA
GATATACCATGGGCAGCAGCCATCACCATCATCACCACAGCCAGGATCCGAATTCGAGCTCG
GCGCGCCTGCAGGTCGACAAGCTTGCGGCCGCATAATGCTTAAGTCGAACAGAAAGTAATCG
TATTGTACACGGCCGCATAATCGAAATTAATACGACTCACTATAGGGGAATTGTGAGCGGAT
AACAATTCCCCATCTTAGTATATTAGTTAAGTATAAGAAGGAGATATACATATGATGGCTAA
GCAAGATTATTACGAGATTTTAGGCGTTTCCAAAACAGCGGAAGAGCGTGAAATCAGAAAG
GCCTACAAACGCCTGGCCATGAAATACCACCCGGACCGTAACCAGGGTGACAAAGAGGCCG
AGGCGAAATTTAAAGAGATCAAGGAAGCTTATGAAGTTCTGACCGACTCGCAAAAACGTGC
GGCATACGATCAGTATGGTCATGCTGCGTTTGAGCAAGGTGGCATGGGCGGCGGCGGTTTTG
GCGGCGGCGCAGACTTCAGCGATATTTTTGGTGACGTTTTCGGCGATATTTTTGGCGGCGGAC
GTGGTCGTCAACGTGCGGCGCGCGGTGCTGATTTACGCTATAACATGGAGCTCACCCTCGAA
GAAGCTGTACGTGGCGTGACCAAAGAGATCCGCATTCCGACTCTGGAAGAGTGTGACGTTTG
CCACGGTAGCGGTGCAAAACCAGGTACACAGCCGCAGACTTGTCCGACCTGTCATGGTTCTG
GTCAGGTGCAGATGCGCCAGGGATTCTTCGCTGTACAGCAGACCTGTCCACACTGTCAGGGC
CGCGGTACGCTGATCAAAGATCCGTGCAACAAATGTCATGGTCATGGTCGTGTTGAGCGCAG
CAAAACGCTGTCCGTTAAAATCCCGGCAGGGGTGGACACTGGAGACCGCATCCGTCTTGCGG

GCGAAGGTGAAGCGGGCGAGCATGGCGCACCGGCAGGCGATCTGTACGTTCAGGTTCAGGT
TAAACAGCACCCGATTTTCGAGCGTGAAGGCAACAACCTGTATTGCGAAGTCCCGATCAACT
TCGCTATGGCGGCGCTGGGTGGCGAAATCGAAGTACCGACCCTTGATGGTCGCGTCAAACTG
AAAGTGCCTGGCGAAACCCAGACCGGTAAGCTATTCCGTATGCGCGGTAAAGGCGTCAAGTC
TGTCCGCGGTGGCGCACAGGGTGATTTGCTGTGCCGCGTTGTCGTCGAAACACCGGTAGGCC
TGAACGAAAGGCAGAAACAGCTGCTGCAAGAGCTGCAAGAAAGCTTCGGTGGCCCAACCGG
CGAGCACAACAGCCCGCGCTCAAAGAGCTTCTTTGATGGTGTGAAGAAGTTTTTTGACGACC
TGACCCGCTAACTCGAGTCTGGTAAAGAAACCGCTGCTGCGAAATTTGAACGCCAGCACATG
GACTCGTCTACTAGCGCAGCTTAATTAACCTAGGCTGCTGCCACCGCTGAGCAATAACTAGC
ATAACCCCTTGGGGCCTCTAAACGGGTCTTGAGGGGTTTTTTGCTGAAACCTCAGGCATTTGA
GAAGCACACGGTCACACTGCTTCCGGTAGTCAATAAACCGGTAAACCAGCAATAGACATAA
GCGGCTATTTAACGACCCTGCCCTGAACCGACGACCGGGTCGAATTTGCTTTCGAATTTCTGC
CATTCATCCGCTTATTATCACTTATTCAGGCGTAGCACCAGGCGTTTAAGGGCACCAATAACT
GCCTTAAAAAAATTACGCCCCGCCCTGCCACTCATCGCAGTACTGTTGTAATTCATTAAGCAT
TCTGCCGACATGGAAGCCATCACAGACGGCATGATGAACCTGAATCGCCAGCGGCATCAGCA
CCTTGTCGCCTTGCGTATAATATTTGCCCATAGTGAAAACGGGGGCGAAGAAGTTGTCCATA
TTGGCCACGTTTAAATCAAAACTGGTGAAACTCACCCAGGGATTGGCTGAGACGAAAAACAT
ATTCTCAATAAACCCTTTAGGGAAATAGGCCAGGTTTTCACCGTAACACGCCACATCTTGCG
AATATATGTGTAGAAACTGCCGGAAATCGTCGTGGTATTCACTCCAGAGCGATGAAAACGTT
TCAGTTTGCTCATGGAAAACGGTGTAACAAGGGTGAACACTATCCCATATCACCAGCTCACC
GTCTTTCATTGCCATACGGAACTCCGGATGAGCATTCATCAGGCGGGCAAGAATGTGAATAA
AGGCCGGATAAAACTTGTGCTTATTTTTCTTTACGGTCTTTAAAAAGGCCGTAATATCCAGCT
GAACGGTCTGGTTATAGGTACATTGAGCAACTGACTGAAATGCCTCAAAATGTTCTTTACGA
TGCCATTGGGATATATCAACGGTGGTATATCCAGTGATTTTTTTCTCCATTTTAGCTTCCTTAG
CTCCTGAAAATCTCGATAACTCAAAAAATACGCCCGGTAGTGATCTTATTTCATTATGGTGAA
AGTTGGAACCTCTTACGTGCCGATCAACGTCTCATTTTCGCCAAAAGTTGGCCCAGGGCTTCC
CGGTATCAACAGGGACACCAGGATTTATTTATTCTGCGAAGTGATCTTCCGTCACAGGTATTT
ATTCGGCGCAAAGTGCGTCGGGTGATGCTGCCAACTTACTGATTTAGTGTATGATGGTGTTTT
TGAGGTGCTCCAGTGGCTTCTGTTTCTATCAGCTGTCCCTCCTGTTCAGCTACTGACGGGGTG
GTGCGTAACGGCAAAAGCACCGCCGGACATCAGCGCTAGCGGAGTGTATACTGGCTTACTAT
GTTGGCACTGATGAGGGTGTCAGTGAAGTGCTTCATGTGGCAGGAGAAAAAAGGCTGCACC
GGTGCGTCAGCAGAATATGTGATACAGGATATATTCCGCTTCCTCGCTCACTGACTCGCTACG
CTCGGTCGTTCGACTGCGGCGAGCGGAAATGGCTTACGAACGGGGCGGAGATTTCCTGGAAG
ATGCCAGGAAGATACTTAACAGGGAAGTGAGAGGGCCGCGGCAAAGCCGTTTTTCCATAGG

CTCCGCCCCCCTGACAAGCATCACGAAATCTGACGCTCAAATCAGTGGTGGCGAAACCCGAC
AGGACTATAAAGATACCAGGCGTTTCCCCTGGCGGCTCCCTCGTGCGCTCTCCTGTTCCTGCC
TTTCGGTTTACCGGTGTCATTCCGCTGTTATGGCCGCGTTTGTCTCATTCCACGCCTGACACTC
AGTTCCGGGTAGGCAGTTCGCTCCAAGCTGGACTGTATGCACGAACCCCCCGTTCAGTCCGA
CCGCTGCGCCTTATCCGGTAACTATCGTCTTGAGTCCAACCCGGAAAGACATGCAAAAGCAC
CACTGGCAGCAGCCACTGGTAATTGATTTAGAGGAGTTAGTCTTGAAGTCATGCGCCGGTTA
AGGCTAAACTGAAAGGACAAGTTTTGGTGACTGCGCTCCTCCAAGCCAGTTACCTCGGTTCA
AAGAGTTGGTAGCTCAGAGAACCTTCGAAAAACCGCCCTGCAAGGCGGTTTTTTCGTTTTCA
GAGCAAGAGATTACGCGCAGACCAAAACGATCTCAAGAAGATCATCTTATTAATCAGATAA
AATATTTCTAGATTTCAGTGCAATTTATCTCTTCAAATGTAGCACCTGAAGTCAGCCCCATAC
GATATAAGTTGTAATTCTCATGTTAGTCATGCCCCGCGCCCACCGGAAGGAGCTGACTGGGT
TGAAGGCTCTCAAGGGCATCGGTCGAGATCCCGGTGCCTAATGAGTGAGCTAACTTACATTA
ATTGCGTTGCGCTCACTGCCCGCTTTCCAGTCGGGAAACCTGTCGTGCCAGCTGCATTAATGA
ATCGGCCAACGCGCGGGGAGAGGCGGTTTGCGTATTGGGCGCCAGGGTGGTTTTTCTTTTCA
CCAGTGAGACGGGCAACAGCTGATTGCCCTTCACCGCCTGGCCCTGAGAGAGTTGCAGCAAG
CGGTCCACGCTGGTTTGCCCCAGCAGGCGAAAATCCTGTTTGATGGTGGTTAACGGCGGGAT
ATAACATGAGCTGTCTTCGGTATCGTCGTATCCCACTACCGAGATGTCCGCACCAACGCGCA
GCCCGGACTCGGTAATGGCGCGCATTGCGCCCAGCGCCATCTGATCGTTGGCAACCAGCATC
GCAGTGGGAACGATGCCCTCATTCAGCATTTGCATGGTTTGTTGAAAACCGGACATGGCACT
CCAGTCGCCTTCCCGTTCCGCTATCGGCTGAATTTGATTGCGAGTGAGATATTTATGCCAGCC
AGCCAGACGCAGACGCGCCGAGACAGAACTTAATGGGCCCGCTAACAGCGCGATTTGCTGG
TGACCCAATGCGACCAGATGCTCCACGCCCAGTCGCGTACCGTCTTCATGGGAGAAAATAAT
ACTGTTGATGGGTGTCTGGTCAGAGACATCAAGAAATAACGCCGGAACATTAGTGCAGGCAG
CTTCCACAGCAATGGCATCCTGGTCATCCAGCGGATAGTTAATGATCAGCCCACTGACGCGT
TGCGCGAGAAGATTGTGCACCGCCGCTTTACAGGCTTCGACGCCGCTTCGTTCTACCATCGAC
ACCACCACGCTGGCACCCAGTTGATCGGCGCGAGATTTAATCGCCGCGACAATTTGCGACGG
CGCGTGCAGGGCCAGACTGGAGGTGGCAACGCCAATCAGCAACGACTGTTTGCCCGCCAGTT
GTTGTGCCACGCGGTTGGGAATGTAATTCAGCTCCGCCATCGCCGCTTCCACTTTTTCCCGCG
TTTTCGCAGAAACGTGGCTGGCCTGGTTCACCACGCGGGAAACGGTCTGATAAGAGACACCG
GCATACTCTGCGACATCGTATAACGTTACTGGTTTCACATTCACCACCCTGAATTGACTCTCT
TCCGGGCGCTATCATGCCATACCGCGAAAGGTTTTGCGCCATTCGATGGTGTCCGGGATCTCG
ACGCTCTCCCTTATGCGACTCCTGCATTAGGAAATTAATACGACTCACTATA

j. pDuet::DnaKJ

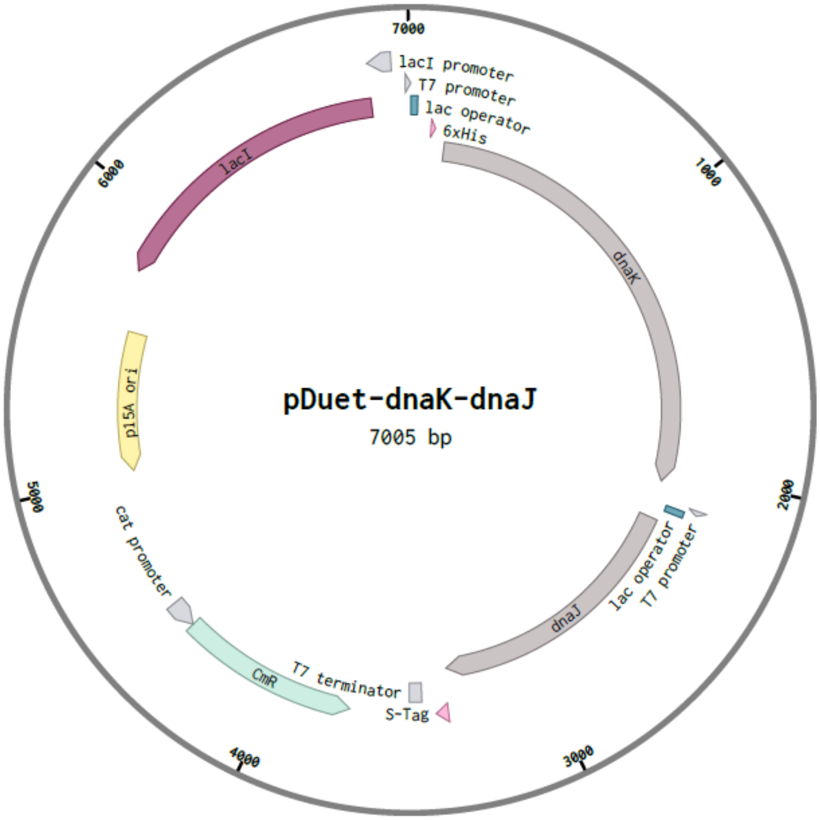

GGGGAATTGTGAGCGGATAACAATTCCCCTGTAGAAATAATTTTGTTTAACTTTAATAAGGA
GATATACCATGGGCAGCAGCCATCACCATCATCACCACAGCCAGGATCCGAATTCGAGCTCG
GCGCGCCTGCAGGTCGACATGGGTAAAATAATTGGTATCGACCTGGGTACTACCAACTCTTG
TGTAGCGATTATGGATGGCACCACTCCTCGCGTGCTGGAGAACGCCGAAGGCGATCGCACCA
CGCCTTCTATCATTGCCTATACCCAGGATGGTGAAACTCTAGTTGGTCAGCCGGCTAAACGTC
AGGCAGTGACGAACCCGCAAAACACTCTGTTTGCGATTAAACGCCTGATTGGTCGCCGCTTC
CAGGACGAAGAAGTACAGCGTGATGTTTCCATCATGCCGTTCAAAATTATTGCTGCTGATAA
CGGCGACGCATGGGTCGAAGTTAAAGGCCAGAAAATGGCACCGCCGCAGATTTCTGCTGAA
GTGCTGAAAAAAATGAAGAAAACCGCTGAAGATTACCTGGGTGAACCGGTAACTGAAGCTG
TTATCACCGTACCGGCATACTTTAACGATGCTCAGCGTCAGGCAACCAAAGACGCAGGCCGT
ATCGCTGGTCTGGAAGTAAAACGTATCATCAACGAACCGACCGCAGCTGCGCTGGCTTACGG
TCTGGACAAAGGCACTGGCAACCGTACTATCGCGGTTTATGACCTGGGTGGTGGTACTTTCG
ATATTTCTATTATCGAAATCGACGAAGTTGACGGCGAAAAAACCTTCGAAGTTCTGGCAACC
AACGGTGATACCCACCTGGGGGGTGAAGACTTCGACAGCCGTCTGATCAACTATCTGGTTGA
AGAATTCAAGAAAGATCAGGGCATTGACCTGCGCAACGATCCGCTGGCAATGCAGCGCCTG
AAAGAAGCGGCAGAAAAAGCGAAAATCGAACTGTCTTCCGCTCAGCAGACCGACGTTAACC

TGCCATACATCACTGCAGACGCGACCGGTCCGAAACACATGAACATCAAAGTGACTCGTGCG
AAACTGGAAAGCCTGGTTGAAGATCTGGTAAACCGTTCCATTGAGCCGCTGAAAGTTGCACT
GCAGGACGCTGGCCTGTCCGTATCTGATATCGACGACGTTATCCTCGTTGGTGGTCAGACTCG
TATGCCAATGGTTCAGAAGAAAGTTGCTGAGTTCTTTGGTAAAGAGCCGCGTAAAGACGTTA
ACCCGGACGAAGCTGTAGCAATCGGTGCTGCTGTTCAGGGTGGTGTTCTGACTGGTGACGTA
AAAGACGTACTGCTGCTGGACGTTACCCCGCTGTCTCTGGGTATCGAAACCATGGGCGGTGT
GATGACGACGCTGATCGCGAAAAACACCACTATCCCGACCAAGCACAGCCAGGTGTTCTCTA
CCGCTGAAGACAACCAGTCTGCGGTAACCATCCATGTGCTGCAGGGTGAACGTAAACGTGCG
GCTGATAACAAATCTCTGGGTCAGTTCAACCTAGATGGTATCAACCCGGCACCGCGCGGCAT
GCCGCAGATCGAAGTTACCTTCGATATCGATGCTGACGGTATCCTGCACGTTTCCGCGAAAG
ATAAAAACAGCGGTAAAGAGCAGAAGATCACCATCAAGGCTTCTTCTGGTCTGAACGAAGA
TGAAATCCAGAAAATGGTACGCGACGCAGAAGCTAACGCCGAAGCTGACCGTAAGTTTGAA
GAGCTGGTACAGACTCGCAACCAGGGCGACCATCTGCTGCACAGCACCCGTAAGCAGGTTGA
AGAAGCAGGCGACAAACTGCCGGCTGACGACAAAACTGCTATCGAGTCTGCGCTGACTGCA
CTGGAAACTGCTCTGAAAGGTGAAGACAAAGCCGCTATCGAAGCGAAAATGCAGGAACTGG
CACAGGTTTCCCAGAAACTGATGGAAATCGCCCAGCAGCAACATGCCCAGCAGCAGACTGCC
GGTGCTGATGCTTCTGCAAACAACGCGAAAGATGACGATGTTGTCGACGCTGAATTTGAAGA
AGTCAAAGACAAAAAATAAAAGCTTGCGGCCGCATAATGCTTAAGTCGAACAGAAAGTAAT
CGTATTGTACACGGCCGCATAATCGAAATTAATACGACTCACTATAGGGGAATTGTGAGCGG
ATAACAATTCCCCATCTTAGTATATTAGTTAAGTATAAGAAGGAGATATACATATGATGGCT
AAGCAAGATTATTACGAGATTTTAGGCGTTTCCAAAACAGCGGAAGAGCGTGAAATCAGAA
AGGCCTACAAACGCCTGGCCATGAAATACCACCCGGACCGTAACCAGGGTGACAAAGAGGC
CGAGGCGAAATTTAAAGAGATCAAGGAAGCTTATGAAGTTCTGACCGACTCGCAAAAACGT
GCGGCATACGATCAGTATGGTCATGCTGCGTTTGAGCAAGGTGGCATGGGCGGCGGCGGTTT
TGGCGGCGGCGCAGACTTCAGCGATATTTTTGGTGACGTTTTCGGCGATATTTTTGGCGGCGG
ACGTGGTCGTCAACGTGCGGCGCGCGGTGCTGATTTACGCTATAACATGGAGCTCACCCTCG
AAGAAGCTGTACGTGGCGTGACCAAAGAGATCCGCATTCCGACTCTGGAAGAGTGTGACGTT
TGCCACGGTAGCGGTGCAAAACCAGGTACACAGCCGCAGACTTGTCCGACCTGTCATGGTTC
TGGTCAGGTGCAGATGCGCCAGGGATTCTTCGCTGTACAGCAGACCTGTCCACACTGTCAGG
GCCGCGGTACGCTGATCAAAGATCCGTGCAACAAATGTCATGGTCATGGTCGTGTTGAGCGC
AGCAAAACGCTGTCCGTTAAAATCCCGGCAGGGGTGGACACTGGAGACCGCATCCGTCTTGC
GGGCGAAGGTGAAGCGGGCGAGCATGGCGCACCGGCAGGCGATCTGTACGTTCAGGTTCAG
GTTAAACAGCACCCGATTTTCGAGCGTGAAGGCAACAACCTGTATTGCGAAGTCCCGATCAA
CTTCGCTATGGCGGCGCTGGGTGGCGAAATCGAAGTACCGACCCTTGATGGTCGCGTCAAAC

TGAAAGTGCCTGGCGAAACCCAGACCGGTAAGCTATTCCGTATGCGCGGTAAAGGCGTCAAG
TCTGTCCGCGGTGGCGCACAGGGTGATTTGCTGTGCCGCGTTGTCGTCGAAACACCGGTAGG
CCTGAACGAAAGGCAGAAACAGCTGCTGCAAGAGCTGCAAGAAAGCTTCGGTGGCCCAACC
GGCGAGCACAACAGCCCGCGCTCAAAGAGCTTCTTTGATGGTGTGAAGAAGTTTTTTGACGA
CCTGACCCGCTAACTCGAGTCTGGTAAAGAAACCGCTGCTGCGAAATTTGAACGCCAGCACA
TGGACTCGTCTACTAGCGCAGCTTAATTAACCTAGGCTGCTGCCACCGCTGAGCAATAACTA
GCATAACCCCTTGGGGCCTCTAAACGGGTCTTGAGGGGTTTTTTGCTGAAACCTCAGGCATTT
GAGAAGCACACGGTCACACTGCTTCCGGTAGTCAATAAACCGGTAAACCAGCAATAGACAT
AAGCGGCTATTTAACGACCCTGCCCTGAACCGACGACCGGGTCGAATTTGCTTTCGAATTTCT
GCCATTCATCCGCTTATTATCACTTATTCAGGCGTAGCACCAGGCGTTTAAGGGCACCAATAA
CTGCCTTAAAAAAATTACGCCCCGCCCTGCCACTCATCGCAGTACTGTTGTAATTCATTAAGC
ATTCTGCCGACATGGAAGCCATCACAGACGGCATGATGAACCTGAATCGCCAGCGGCATCAG
CACCTTGTCGCCTTGCGTATAATATTTGCCCATAGTGAAAACGGGGGCGAAGAAGTTGTCCA
TATTGGCCACGTTTAAATCAAAACTGGTGAAACTCACCCAGGGATTGGCTGAGACGAAAAAC
ATATTCTCAATAAACCCTTTAGGGAAATAGGCCAGGTTTTCACCGTAACACGCCACATCTTGC
GAATATATGTGTAGAAACTGCCGGAAATCGTCGTGGTATTCACTCCAGAGCGATGAAAACGT
TTCAGTTTGCTCATGGAAAACGGTGTAACAAGGGTGAACACTATCCCATATCACCAGCTCAC
CGTCTTTCATTGCCATACGGAACTCCGGATGAGCATTCATCAGGCGGGCAAGAATGTGAATA
AAGGCCGGATAAAACTTGTGCTTATTTTTCTTTACGGTCTTTAAAAAGGCCGTAATATCCAGC
TGAACGGTCTGGTTATAGGTACATTGAGCAACTGACTGAAATGCCTCAAAATGTTCTTTACG
ATGCCATTGGGATATATCAACGGTGGTATATCCAGTGATTTTTTTCTCCATTTTAGCTTCCTTA
GCTCCTGAAAATCTCGATAACTCAAAAAATACGCCCGGTAGTGATCTTATTTCATTATGGTGA
AAGTTGGAACCTCTTACGTGCCGATCAACGTCTCATTTTCGCCAAAAGTTGGCCCAGGGCTTC
CCGGTATCAACAGGGACACCAGGATTTATTTATTCTGCGAAGTGATCTTCCGTCACAGGTATT
TATTCGGCGCAAAGTGCGTCGGGTGATGCTGCCAACTTACTGATTTAGTGTATGATGGTGTTT
TTGAGGTGCTCCAGTGGCTTCTGTTTCTATCAGCTGTCCCTCCTGTTCAGCTACTGACGGGGT
GGTGCGTAACGGCAAAAGCACCGCCGGACATCAGCGCTAGCGGAGTGTATACTGGCTTACTA
TGTTGGCACTGATGAGGGTGTCAGTGAAGTGCTTCATGTGGCAGGAGAAAAAAGGCTGCACC
GGTGCGTCAGCAGAATATGTGATACAGGATATATTCCGCTTCCTCGCTCACTGACTCGCTACG
CTCGGTCGTTCGACTGCGGCGAGCGGAAATGGCTTACGAACGGGGCGGAGATTTCCTGGAAG
ATGCCAGGAAGATACTTAACAGGGAAGTGAGAGGGCCGCGGCAAAGCCGTTTTTCCATAGG
CTCCGCCCCCCTGACAAGCATCACGAAATCTGACGCTCAAATCAGTGGTGGCGAAACCCGAC
AGGACTATAAAGATACCAGGCGTTTCCCCTGGCGGCTCCCTCGTGCGCTCTCCTGTTCCTGCC
TTTCGGTTTACCGGTGTCATTCCGCTGTTATGGCCGCGTTTGTCTCATTCCACGCCTGACACTC

AGTTCCGGGTAGGCAGTTCGCTCCAAGCTGGACTGTATGCACGAACCCCCCGTTCAGTCCGA
CCGCTGCGCCTTATCCGGTAACTATCGTCTTGAGTCCAACCCGGAAAGACATGCAAAAGCAC
CACTGGCAGCAGCCACTGGTAATTGATTTAGAGGAGTTAGTCTTGAAGTCATGCGCCGGTTA
AGGCTAAACTGAAAGGACAAGTTTTGGTGACTGCGCTCCTCCAAGCCAGTTACCTCGGTTCA
AAGAGTTGGTAGCTCAGAGAACCTTCGAAAAACCGCCCTGCAAGGCGGTTTTTTCGTTTTCA
GAGCAAGAGATTACGCGCAGACCAAAACGATCTCAAGAAGATCATCTTATTAATCAGATAA
AATATTTCTAGATTTCAGTGCAATTTATCTCTTCAAATGTAGCACCTGAAGTCAGCCCCATAC
GATATAAGTTGTAATTCTCATGTTAGTCATGCCCCGCGCCCACCGGAAGGAGCTGACTGGGT
TGAAGGCTCTCAAGGGCATCGGTCGAGATCCCGGTGCCTAATGAGTGAGCTAACTTACATTA
ATTGCGTTGCGCTCACTGCCCGCTTTCCAGTCGGGAAACCTGTCGTGCCAGCTGCATTAATGA
ATCGGCCAACGCGCGGGGAGAGGCGGTTTGCGTATTGGGCGCCAGGGTGGTTTTTCTTTTCA
CCAGTGAGACGGGCAACAGCTGATTGCCCTTCACCGCCTGGCCCTGAGAGAGTTGCAGCAAG
CGGTCCACGCTGGTTTGCCCCAGCAGGCGAAAATCCTGTTTGATGGTGGTTAACGGCGGGAT
ATAACATGAGCTGTCTTCGGTATCGTCGTATCCCACTACCGAGATGTCCGCACCAACGCGCA
GCCCGGACTCGGTAATGGCGCGCATTGCGCCCAGCGCCATCTGATCGTTGGCAACCAGCATC
GCAGTGGGAACGATGCCCTCATTCAGCATTTGCATGGTTTGTTGAAAACCGGACATGGCACT
CCAGTCGCCTTCCCGTTCCGCTATCGGCTGAATTTGATTGCGAGTGAGATATTTATGCCAGCC
AGCCAGACGCAGACGCGCCGAGACAGAACTTAATGGGCCCGCTAACAGCGCGATTTGCTGG
TGACCCAATGCGACCAGATGCTCCACGCCCAGTCGCGTACCGTCTTCATGGGAGAAAATAAT
ACTGTTGATGGGTGTCTGGTCAGAGACATCAAGAAATAACGCCGGAACATTAGTGCAGGCAG
CTTCCACAGCAATGGCATCCTGGTCATCCAGCGGATAGTTAATGATCAGCCCACTGACGCGT
TGCGCGAGAAGATTGTGCACCGCCGCTTTACAGGCTTCGACGCCGCTTCGTTCTACCATCGAC
ACCACCACGCTGGCACCCAGTTGATCGGCGCGAGATTTAATCGCCGCGACAATTTGCGACGG
CGCGTGCAGGGCCAGACTGGAGGTGGCAACGCCAATCAGCAACGACTGTTTGCCCGCCAGTT
GTTGTGCCACGCGGTTGGGAATGTAATTCAGCTCCGCCATCGCCGCTTCCACTTTTTCCCGCG
TTTTCGCAGAAACGTGGCTGGCCTGGTTCACCACGCGGGAAACGGTCTGATAAGAGACACCG
GCATACTCTGCGACATCGTATAACGTTACTGGTTTCACATTCACCACCCTGAATTGACTCTCT
TCCGGGCGCTATCATGCCATACCGCGAAAGGTTTTGCGCCATTCGATGGTGTCCGGGATCTCG
ACGCTCTCCCTTATGCGACTCCTGCATTAGGAAATTAATACGACTCACTATA

k. pDuet::IbpA

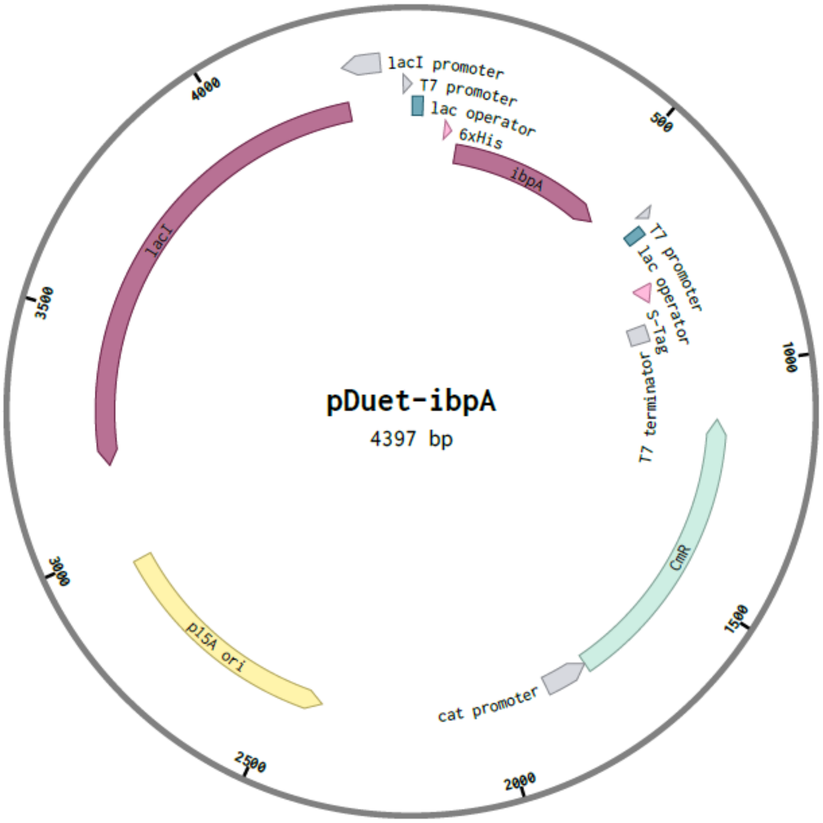

GGGGAATTGTGAGCGGATAACAATTCCCCTGTAGAAATAATTTTGTTTAACTTTAATAAGGA
GATATACCATGGGCAGCAGCCATCACCATCATCACCACAGCCAGGATCCGAATTCATGCGTA
ACTTTGATTTATCCCCGCTTTACCGTTCTGCTATTGGATTTGACCGTTTGTTTAACCACTTAGA
AAACAACCAGAGCCAGAGTAATGGCGGCTACCCTCCGTATAACGTTGAACTGGTAGACGAA
AACCATTACCGCATTGCTATCGCTGTGGCTGGTTTTGCTGAGAGCGAACTGGAAATTACCGCC
CAGGATAATCTGCTGGTGGTGAAAGGTGCTCACGCCGACGAACAAAAAGAGCGCACCTATCT
GTACCAGGGCATCGCTGAACGCAACTTTGAACGCAAATTCCAGTTAGCTGAGAACATTCATG
TTCGTGGTGCTAACCTGGTAAATGGTTTGCTGTATATCGATCTCGAACGCGTGATTCCGGAAG
CGAAAAAACCGCGCCGTATCGAAATCAACTAAAAGCTTGCGGCCGCATAATGCTTAAGTCGA
ACAGAAAGTAATCGTATTGTACACGGCCGCATAATCGAAATTAATACGACTCACTATAGGGG
AATTGTGAGCGGATAACAATTCCCCATCTTAGTATATTAGTTAAGTATAAGAAGGAGATATA
CATATGGCAGATCTCAATTGGATATCGGCCGGCCACGCGATCGCTGACGTCGGTACCCTCGA
GTCTGGTAAAGAAACCGCTGCTGCGAAATTTGAACGCCAGCACATGGACTCGTCTACTAGCG
CAGCTTAATTAACCTAGGCTGCTGCCACCGCTGAGCAATAACTAGCATAACCCCTTGGGGCC
TCTAAACGGGTCTTGAGGGGTTTTTTGCTGAAACCTCAGGCATTTGAGAAGCACACGGTCAC
ACTGCTTCCGGTAGTCAATAAACCGGTAAACCAGCAATAGACATAAGCGGCTATTTAACGAC

CCTGCCCTGAACCGACGACCGGGTCGAATTTGCTTTCGAATTTCTGCCATTCATCCGCTTATT
ATCACTTATTCAGGCGTAGCACCAGGCGTTTAAGGGCACCAATAACTGCCTTAAAAAAATTA
CGCCCCGCCCTGCCACTCATCGCAGTACTGTTGTAATTCATTAAGCATTCTGCCGACATGGAA
GCCATCACAGACGGCATGATGAACCTGAATCGCCAGCGGCATCAGCACCTTGTCGCCTTGCG
TATAATATTTGCCCATAGTGAAAACGGGGGCGAAGAAGTTGTCCATATTGGCCACGTTTAAA
TCAAAACTGGTGAAACTCACCCAGGGATTGGCTGAGACGAAAAACATATTCTCAATAAACCC
TTTAGGGAAATAGGCCAGGTTTTCACCGTAACACGCCACATCTTGCGAATATATGTGTAGAA
ACTGCCGGAAATCGTCGTGGTATTCACTCCAGAGCGATGAAAACGTTTCAGTTTGCTCATGG
AAAACGGTGTAACAAGGGTGAACACTATCCCATATCACCAGCTCACCGTCTTTCATTGCCAT
ACGGAACTCCGGATGAGCATTCATCAGGCGGGCAAGAATGTGAATAAAGGCCGGATAAAAC
TTGTGCTTATTTTTCTTTACGGTCTTTAAAAAGGCCGTAATATCCAGCTGAACGGTCTGGTTAT
AGGTACATTGAGCAACTGACTGAAATGCCTCAAAATGTTCTTTACGATGCCATTGGGATATA
TCAACGGTGGTATATCCAGTGATTTTTTTCTCCATTTTAGCTTCCTTAGCTCCTGAAAATCTCG
ATAACTCAAAAAATACGCCCGGTAGTGATCTTATTTCATTATGGTGAAAGTTGGAACCTCTTA
CGTGCCGATCAACGTCTCATTTTCGCCAAAAGTTGGCCCAGGGCTTCCCGGTATCAACAGGG
ACACCAGGATTTATTTATTCTGCGAAGTGATCTTCCGTCACAGGTATTTATTCGGCGCAAAGT
GCGTCGGGTGATGCTGCCAACTTACTGATTTAGTGTATGATGGTGTTTTTGAGGTGCTCCAGT
GGCTTCTGTTTCTATCAGCTGTCCCTCCTGTTCAGCTACTGACGGGGTGGTGCGTAACGGCAA
AAGCACCGCCGGACATCAGCGCTAGCGGAGTGTATACTGGCTTACTATGTTGGCACTGATGA
GGGTGTCAGTGAAGTGCTTCATGTGGCAGGAGAAAAAAGGCTGCACCGGTGCGTCAGCAGA
ATATGTGATACAGGATATATTCCGCTTCCTCGCTCACTGACTCGCTACGCTCGGTCGTTCGAC
TGCGGCGAGCGGAAATGGCTTACGAACGGGGCGGAGATTTCCTGGAAGATGCCAGGAAGAT
ACTTAACAGGGAAGTGAGAGGGCCGCGGCAAAGCCGTTTTTCCATAGGCTCCGCCCCCCTGA
CAAGCATCACGAAATCTGACGCTCAAATCAGTGGTGGCGAAACCCGACAGGACTATAAAGA
TACCAGGCGTTTCCCCCTGGCGGCTCCCTCGTGCGCTCTCCTGTTCCTGCCTTTCGGTTTACCGG
TGTCATTCCGCTGTTATGGCCGCGTTTGTCTCATTCCACGCCTGACACTCAGTTCCGGGTAGG
CAGTTCGCTCCAAGCTGGACTGTATGCACGAACCCCCCGTTCAGTCCGACCGCTGCGCCTTAT
CCGGTAACTATCGTCTTGAGTCCAACCCGGAAAGACATGCAAAAGCACCACTGGCAGCAGCC
ACTGGTAATTGATTTAGAGGAGTTAGTCTTGAAGTCATGCGCCGGTTAAGGCTAAACTGAAA
GGACAAGTTTTGGTGACTGCGCTCCTCCAAGCCAGTTACCTCGGTTCAAAGAGTTGGTAGCTC
AGAGAACCTTCGAAAAACCGCCCTGCAAGGCGGTTTTTTCGTTTTCAGAGCAAGAGATTACG
CGCAGACCAAAACGATCTCAAGAAGATCATCTTATTAATCAGATAAAATATTTCTAGATTTC
AGTGCAATTTATCTCTTCAAATGTAGCACCTGAAGTCAGCCCCATACGATATAAGTTGTAATT
CTCATGTTAGTCATGCCCCGCGCCCACCGGAAGGAGCTGACTGGGTTGAAGGCTCTCAAGGG

CATCGGTCGAGATCCCGGTGCCTAATGAGTGAGCTAACTTACATTAATTGCGTTGCGCTCACT
GCCCGCTTTCCAGTCGGGAAACCTGTCGTGCCAGCTGCATTAATGAATCGGCCAACGCGCGG
GGAGAGGCGGTTTGCGTATTGGGCGCCAGGGTGGTTTTTCTTTTCACCAGTGAGACGGGCAA
CAGCTGATTGCCCTTCACCGCCTGGCCCTGAGAGAGTTGCAGCAAGCGGTCCACGCTGGTTT
GCCCCAGCAGGCGAAAATCCTGTTTGATGGTGGTTAACGGCGGGATATAACATGAGCTGTCT
TCGGTATCGTCGTATCCCACTACCGAGATGTCCGCACCAACGCGCAGCCCGGACTCGGTAAT
GGCGCGCATTGCGCCCAGCGCCATCTGATCGTTGGCAACCAGCATCGCAGTGGGAACGATGC
CCTCATTCAGCATTTGCATGGTTTGTTGAAAACCGGACATGGCACTCCAGTCGCCTTCCCGTT
CCGCTATCGGCTGAATTTGATTGCGAGTGAGATATTTATGCCAGCCAGCCAGACGCAGACGC
GCCGAGACAGAACTTAATGGGCCCGCTAACAGCGCGATTTGCTGGTGACCCAATGCGACCAG
ATGCTCCACGCCCAGTCGCGTACCGTCTTCATGGGAGAAAATAATACTGTTGATGGGTGTCT
GGTCAGAGACATCAAGAAATAACGCCGGAACATTAGTGCAGGCAGCTTCCACAGCAATGGC
ATCCTGGTCATCCAGCGGATAGTTAATGATCAGCCCACTGACGCGTTGCGCGAGAAGATTGT
GCACCGCCGCTTTACAGGCTTCGACGCCGCTTCGTTCTACCATCGACACCACCACGCTGGCAC
CCAGTTGATCGGCGCGAGATTTAATCGCCGCGACAATTTGCGACGGCGCGTGCAGGGCCAGA
CTGGAGGTGGCAACGCCAATCAGCAACGACTGTTTGCCCGCCAGTTGTTGTGCCACGCGGTT
GGGAATGTAATTCAGCTCCGCCATCGCCGCTTCCACTTTTTCCCGCGTTTTCGCAGAAACGTG
GCTGGCCTGGTTCACCACGCGGGAAACGGTCTGATAAGAGACACCGGCATACTCTGCGACAT
CGTATAACGTTACTGGTTTCACATTCACCACCCTGAATTGACTCTCTTCCGGGCGCTATCATG
CCATACCGCGAAAGGTTTTGCGCCATTCGATGGTGTCCGGGATCTCGACGCTCTCCCTTATGC
GACTCCTGCATTAGGAAATTAATACGACTCACTATA

l. pDuet::IbpB

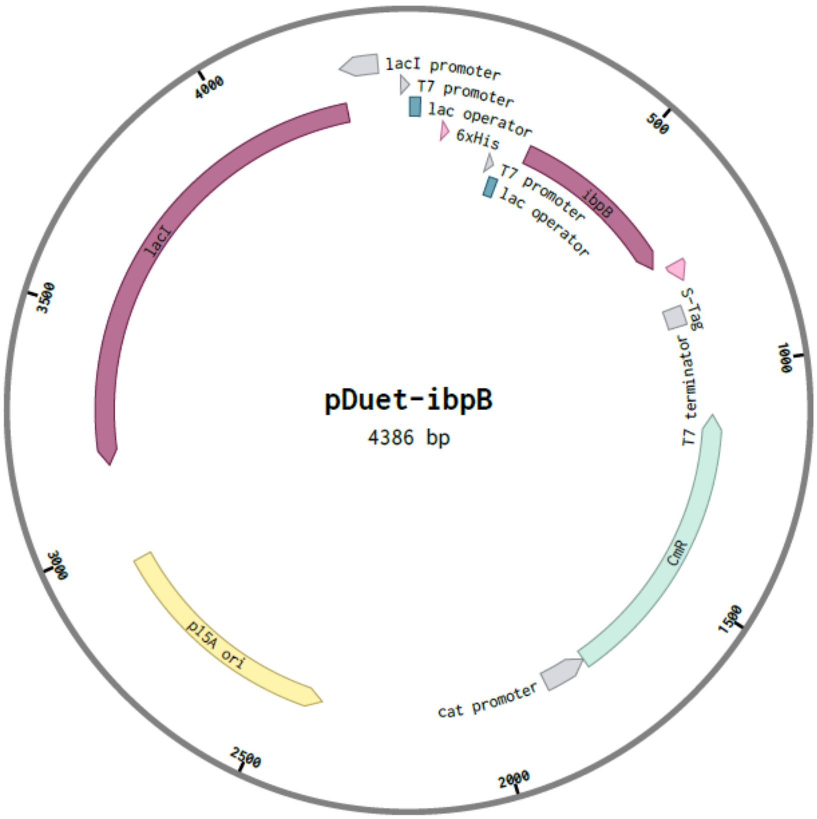

GGGGAATTGTGAGCGGATAACAATTCCCCTGTAGAAATAATTTTGTTTAACTTTAATAAGGA
GATATACCATGGGCAGCAGCCATCACCATCATCACCACAGCCAGGATCCGAATTCGAGCTCG
GCGCGCCTGCAGGTCGACAAGCTTGCGGCCGCATAATGCTTAAGTCGAACAGAAAGTAATCG
TATTGTACACGGCCGCATAATCGAAATTAATACGACTCACTATAGGGGAATTGTGAGCGGAT
AACAATTCCCCATCTTAGTATATTAGTTAAGTATAAGAAGGAGATATACATATGATGCGTAA
CTTCGATTTATCCCCACTGATGCGTCAATGGATCGGTTTTGACAAACTGGCCAACGCACTGCA
AAACGCCGGTGAAAGCCAGAGCTTCCCGCCGTACAACATTGAGAAAAGCGACGATAACCAC
TACCGCATTACCCTTGCGCTGGCAGGTTTCCGTCAGGAAGATTTAGAGATTCAACTGGAAGG
TACGCGCCTGAGCGTAAAAGGCACGCCGGAGCAGCCAAAAGAAGAGAAAAAATGGCTGCAT
CAAGGGCTTATGAATCAGCCATTTAGCCTGAGCTTTACGCTGGCTGAAAATATGGAAGTCTC
TGGCGCAACCTTCGTAAACGGTTTACTGCATATTGATTTAATTCGTAATGAGCCTGAACCCAT
CGCAGCGCAGCGTATCGCTATCAGCGAACGTCCCGCGTTAAATAGCTAACTCGAGTCTGGTA
AAGAAACCGCTGCTGCGAAATTTGAACGCCAGCACATGGACTCGTCTACTAGCGCAGCTTAA
TTAACCTAGGCTGCTGCCACCGCTGAGCAATAACTAGCATAACCCCTTGGGGCCTCTAAACG
GGTCTTGAGGGGTTTTTTGCTGAAACCTCAGGCATTTGAGAAGCACACGGTCACACTGCTTCC
GGTAGTCAATAAACCGGTAAACCAGCAATAGACATAAGCGGCTATTTAACGACCCTGCCCTG

AACCGACGACCGGGTCGAATTTGCTTTCGAATTTCTGCCATTCATCCGCTTATTATCACTTATT
CAGGCGTAGCACCAGGCGTTTAAGGGCACCAATAACTGCCTTAAAAAAATTACGCCCCGCCC
TGCCACTCATCGCAGTACTGTTGTAATTCATTAAGCATTCTGCCGACATGGAAGCCATCACAG
ACGGCATGATGAACCTGAATCGCCAGCGGCATCAGCACCTTGTCGCCTTGCGTATAATATTT
GCCCATAGTGAAAACGGGGGCGAAGAAGTTGTCCATATTGGCCACGTTTAAATCAAAACTGG
TGAAACTCACCCAGGGATTGGCTGAGACGAAAAACATATTCTCAATAAACCCTTTAGGGAAA
TAGGCCAGGTTTTCACCGTAACACGCCACATCTTGCGAATATATGTGTAGAAACTGCCGGAA
ATCGTCGTGGTATTCACTCCAGAGCGATGAAAACGTTTCAGTTTGCTCATGGAAAACGGTGT
AACAAGGGTGAACACTATCCCATATCACCAGCTCACCGTCTTTCATTGCCATACGGAACTCC
GGATGAGCATTCATCAGGCGGGCAAGAATGTGAATAAAGGCCGGATAAAACTTGTGCTTATT
TTTCTTTACGGTCTTTAAAAAGGCCGTAATATCCAGCTGAACGGTCTGGTTATAGGTACATTG
AGCAACTGACTGAAATGCCTCAAAATGTTCTTTACGATGCCATTGGGATATATCAACGGTGG
TATATCCAGTGATTTTTTTCTCCATTTTAGCTTCCTTAGCTCCTGAAAATCTCGATAACTCAAA
AAATACGCCCGGTAGTGATCTTATTTCATTATGGTGAAAGTTGGAACCTCTTACGTGCCGATC
AACGTCTCATTTTCGCCAAAAGTTGGCCCAGGGCTTCCCGGTATCAACAGGGACACCAGGAT
TTATTTATTCTGCGAAGTGATCTTCCGTCACAGGTATTTATTCGGCGCAAAGTGCGTCGGGTG
ATGCTGCCAACTTACTGATTTAGTGTATGATGGTGTTTTTGAGGTGCTCCAGTGGCTTCTGTTT
CTATCAGCTGTCCCTCCTGTTCAGCTACTGACGGGGTGGTGCGTAACGGCAAAAGCACCGCC
GGACATCAGCGCTAGCGGAGTGTATACTGGCTTACTATGTTGGCACTGATGAGGGTGTCAGT
GAAGTGCTTCATGTGGCAGGAGAAAAAAGGCTGCACCGGTGCGTCAGCAGAATATGTGATA
CAGGATATATTCCGCTTCCTCGCTCACTGACTCGCTACGCTCGGTCGTTCGACTGCGGCGAGC
GGAAATGGCTTACGAACGGGGCGGAGATTTCCTGGAAGATGCCAGGAAGATACTTAACAGG
GAAGTGAGAGGGCCGCGGCAAAGCCGTTTTTCCATAGGCTCCGCCCCCCTGACAAGCATCAC
GAAATCTGACGCTCAAATCAGTGGTGGCGAAACCCGACAGGACTATAAAGATACCAGGCGT
TTCCCCTGGCGGCTCCCTCGTGCGCTCTCCTGTTCCTGCCTTTCGGTTTACCGGTGTCATTCCG
CTGTTATGGCCGCGTTTGTCTCATTCCACGCCTGACACTCAGTTCCGGGTAGGCAGTTCGCTC
CAAGCTGGACTGTATGCACGAACCCCCCGTTCAGTCCGACCGCTGCGCCTTATCCGGTAACT
ATCGTCTTGAGTCCAACCCGGAAAGACATGCAAAAGCACCACTGGCAGCAGCCACTGGTAAT
TGATTTAGAGGAGTTAGTCTTGAAGTCATGCGCCGGTTAAGGCTAAACTGAAAGGACAAGTT
TTGGTGACTGCGCTCCTCCAAGCCAGTTACCTCGGTTCAAAGAGTTGGTAGCTCAGAGAACC
TTCGAAAAACCGCCCTGCAAGGCGGTTTTTTCGTTTTCAGAGCAAGAGATTACGCGCAGACC
AAAACGATCTCAAGAAGATCATCTTATTAATCAGATAAAATATTTCTAGATTTCAGTGCAATT
TATCTCTTCAAATGTAGCACCTGAAGTCAGCCCCATACGATATAAGTTGTAATTCTCATGTTA
GTCATGCCCCGCGCCCACCGGAAGGAGCTGACTGGGTTGAAGGCTCTCAAGGGCATCGGTCG

AGATCCCGGTGCCTAATGAGTGAGCTAACTTACATTAATTGCGTTGCGCTCACTGCCCGCTTT
CCAGTCGGGAAACCTGTCGTGCCAGCTGCATTAATGAATCGGCCAACGCGCGGGGAGAGGC
GGTTTGCGTATTGGGCGCCAGGGTGGTTTTTCTTTTCACCAGTGAGACGGGCAACAGCTGATT
GCCCTTCACCGCCTGGCCCTGAGAGAGTTGCAGCAAGCGGTCCACGCTGGTTTGCCCCAGCA
GGCGAAAATCCTGTTTGATGGTGGTTAACGGCGGGATATAACATGAGCTGTCTTCGGTATCG
TCGTATCCCACTACCGAGATGTCCGCACCAACGCGCAGCCCGGACTCGGTAATGGCGCGCAT
TGCGCCCAGCGCCATCTGATCGTTGGCAACCAGCATCGCAGTGGGAACGATGCCCTCATTCA
GCATTTGCATGGTTTGTTGAAAACCGGACATGGCACTCCAGTCGCCTTCCCGTTCCGCTATCG
GCTGAATTTGATTGCGAGTGAGATATTTATGCCAGCCAGCCAGACGCAGACGCGCCGAGACA
GAACTTAATGGGCCCGCTAACAGCGCGATTTGCTGGTGACCCAATGCGACCAGATGCTCCAC
GCCCAGTCGCGTACCGTCTTCATGGGAGAAAATAATACTGTTGATGGGTGTCTGGTCAGAGA
CATCAAGAAATAACGCCGGAACATTAGTGCAGGCAGCTTCCACAGCAATGGCATCCTGGTCA
TCCAGCGGATAGTTAATGATCAGCCCACTGACGCGTTGCGCGAGAAGATTGTGCACCGCCGC
TTTACAGGCTTCGACGCCGCTTCGTTCTACCATCGACACCACCACGCTGGCACCCAGTTGATC
GGCGCGAGATTTAATCGCCGCGACAATTTGCGACGGCGCGTGCAGGGCCAGACTGGAGGTG
GCAACGCCAATCAGCAACGACTGTTTGCCCGCCAGTTGTTGTGCCACGCGGTTGGGAATGTA
ATTCAGCTCCGCCATCGCCGCTTCCACTTTTTCCCGCGTTTTCGCAGAAACGTGGCTGGCCTG
GTTCACCACGCGGGAAACGGTCTGATAAGAGACACCGGCATACTCTGCGACATCGTATAACG
TTACTGGTTTCACATTCACCACCCTGAATTGACTCTCTTCCGGGCGCTATCATGCCATACCGC
GAAAGGTTTTGCGCCATTCGATGGTGTCCGGGATCTCGACGCTCTCCCTTATGCGACTCCTGC
ATTAGGAAATTAATACGACTCACTATA

m. pDuet::IbpAB

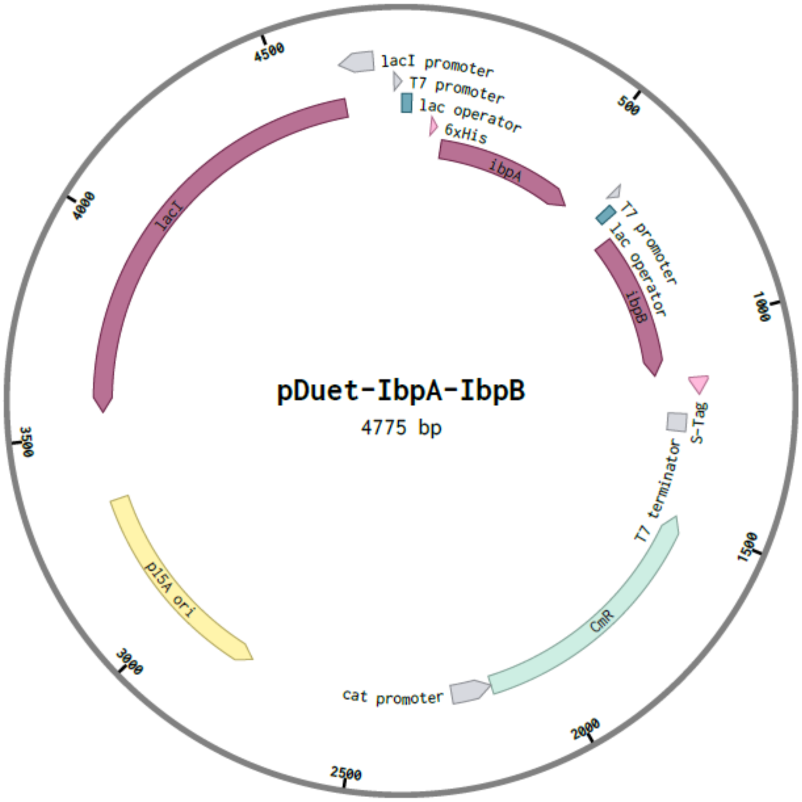

GGGGAATTGTGAGCGGATAACAATTCCCCTGTAGAAATAATTTTGTTTAACTTTAATAAGGA
GATATACCATGGGCAGCAGCCATCACCATCATCACCACAGCCAGGATCCGAATTCATGCGTA
ACTTTGATTTATCCCCGCTTTACCGTTCTGCTATTGGATTTGACCGTTTGTTTAACCACTTAGA
AAACAACCAGAGCCAGAGTAATGGCGGCTACCCTCCGTATAACGTTGAACTGGTAGACGAA
AACCATTACCGCATTGCTATCGCTGTGGCTGGTTTTGCTGAGAGCGAACTGGAAATTACCGCC
CAGGATAATCTGCTGGTGGTGAAAGGTGCTCACGCCGACGAACAAAAAGAGCGCACCTATCT
GTACCAGGGCATCGCTGAACGCAACTTTGAACGCAAATTCCAGTTAGCTGAGAACATTCATG
TTCGTGGTGCTAACCTGGTAAATGGTTTGCTGTATATCGATCTCGAACGCGTGATTCCGGAAG
CGAAAAAACCGCGCCGTATCGAAATCAACTAAAAGCTTGCGGCCGCATAATGCTTAAGTCGA
ACAGAAAGTAATCGTATTGTACACGGCCGCATAATCGAAATTAATACGACTCACTATAGGGG
AATTGTGAGCGGATAACAATTCCCCATCTTAGTATATTAGTTAAGTATAAGAAGGAGATATA
CATATGATGCGTAACTTCGATTTATCCCCACTGATGCGTCAATGGATCGGTTTTGACAAACTG
GCCAACGCACTGCAAAACGCCGGTGAAAGCCAGAGCTTCCCGCCGTACAACATTGAGAAAA
GCGACGATAACCACTACCGCATTACCCTTGCGCTGGCAGGTTTCCGTCAGGAAGATTTAGAG
ATTCAACTGGAAGGTACGCGCCTGAGCGTAAAAGGCACGCCGGAGCAGCCAAAAGAAGAGA
AAAAATGGCTGCATCAAGGGCTTATGAATCAGCCATTTAGCCTGAGCTTTACGCTGGCTGAA

AATATGGAAGTCTCTGGCGCAACCTTCGTAAACGGTTTACTGCATATTGATTTAATTCGTAAT
GAGCCTGAACCCATCGCAGCGCAGCGTATCGCTATCAGCGAACGTCCCGCGTTAAATAGCTA
ACTCGAGTCTGGTAAAGAAACCGCTGCTGCGAAATTTGAACGCCAGCACATGGACTCGTCTA
CTAGCGCAGCTTAATTAACCTAGGCTGCTGCCACCGCTGAGCAATAACTAGCATAACCCCTT
GGGGCCTCTAAACGGGTCTTGAGGGGTTTTTTGCTGAAACCTCAGGCATTTGAGAAGCACAC
GGTCACACTGCTTCCGGTAGTCAATAAACCGGTAAACCAGCAATAGACATAAGCGGCTATTT
AACGACCCTGCCCTGAACCGACGACCGGGTCGAATTTGCTTTCGAATTTCTGCCATTCATCCG
CTTATTATCACTTATTCAGGCGTAGCACCAGGCGTTTAAGGGCACCAATAACTGCCTTAAAA
AAATTACGCCCCGCCCTGCCACTCATCGCAGTACTGTTGTAATTCATTAAGCATTCTGCCGAC
ATGGAAGCCATCACAGACGGCATGATGAACCTGAATCGCCAGCGGCATCAGCACCTTGTCGC
CTTGCGTATAATATTTGCCCATAGTGAAAACGGGGGCGAAGAAGTTGTCCATATTGGCCACG
TTTAAATCAAAACTGGTGAAACTCACCCAGGGATTGGCTGAGACGAAAAACATATTCTCAAT
AAACCCTTTAGGGAAATAGGCCAGGTTTTCACCGTAACACGCCACATCTTGCGAATATATGT
GTAGAAACTGCCGGAAATCGTCGTGGTATTCACTCCAGAGCGATGAAAACGTTTCAGTTTGC
TCATGGAAAACGGTGTAACAAGGGTGAACACTATCCCATATCACCAGCTCACCGTCTTTCAT
TGCCATACGGAACTCCGGATGAGCATTCATCAGGCGGGCAAGAATGTGAATAAAGGCCGGA
TAAAACTTGTGCTTATTTTTCTTTACGGTCTTTAAAAAGGCCGTAATATCCAGCTGAACGGTC
TGGTTATAGGTACATTGAGCAACTGACTGAAATGCCTCAAAATGTTCTTTACGATGCCATTGG
GATATATCAACGGTGGTATATCCAGTGATTTTTTTCTCCATTTTAGCTTCCTTAGCTCCTGAAA
ATCTCGATAACTCAAAAAATACGCCCGGTAGTGATCTTATTTCATTATGGTGAAAGTTGGAA
CCTCTTACGTGCCGATCAACGTCTCATTTTCGCCAAAAGTTGGCCCAGGGCTTCCCGGTATCA
ACAGGGACACCAGGATTTATTTATTCTGCGAAGTGATCTTCCGTCACAGGTATTTATTCGGCG
CAAAGTGCGTCGGGTGATGCTGCCAACTTACTGATTTAGTGTATGATGGTGTTTTTGAGGTGC
TCCAGTGGCTTCTGTTTCTATCAGCTGTCCCTCCTGTTCAGCTACTGACGGGGTGGTGCGTAA
CGGCAAAAGCACCGCCGGACATCAGCGCTAGCGGAGTGTATACTGGCTTACTATGTTGGCAC
TGATGAGGGTGTCAGTGAAGTGCTTCATGTGGCAGGAGAAAAAAGGCTGCACCGGTGCGTC
AGCAGAATATGTGATACAGGATATATTCCGCTTCCTCGCTCACTGACTCGCTACGCTCGGTCG
TTCGACTGCGGCGAGCGGAAATGGCTTACGAACGGGGCGGAGATTTCCTGGAAGATGCCAG
GAAGATACTTAACAGGGAAGTGAGAGGGCCGCGGCAAAGCCGTTTTTCCATAGGCTCCGCCC
CCCTGACAAGCATCACGAAATCTGACGCTCAAATCAGTGGTGGCGAAACCCGACAGGACTAT
AAAGATACCAGGCGTTTCCCCTGGCGGCTCCCTCGTGCGCTCTCCTGTTCCTGCCTTTCGGTTT
ACCGGTGTCATTCCGCTGTTATGGCCGCGTTTGTCTCATTCCACGCCTGACACTCAGTTCCGG
GTAGGCAGTTCGCTCCAAGCTGGACTGTATGCACGAACCCCCCGTTCAGTCCGACCGCTGCG
CCTTATCCGGTAACTATCGTCTTGAGTCCAACCCGGAAAGACATGCAAAAGCACCACTGGCA

GCAGCCACTGGTAATTGATTTAGAGGAGTTAGTCTTGAAGTCATGCGCCGGTTAAGGCTAAA
CTGAAAGGACAAGTTTTGGTGACTGCGCTCCTCCAAGCCAGTTACCTCGGTTCAAAGAGTTG
GTAGCTCAGAGAACCTTCGAAAAACCGCCCTGCAAGGCGGTTTTTTCGTTTTCAGAGCAAGA
GATTACGCGCAGACCAAAACGATCTCAAGAAGATCATCTTATTAATCAGATAAAATATTTCT
AGATTTCAGTGCAATTTATCTCTTCAAATGTAGCACCTGAAGTCAGCCCCATACGATATAAGT
TGTAATTCTCATGTTAGTCATGCCCCGCGCCCACCGGAAGGAGCTGACTGGGTTGAAGGCTC
TCAAGGGCATCGGTCGAGATCCCGGTGCCTAATGAGTGAGCTAACTTACATTAATTGCGTTG
CGCTCACTGCCCGCTTTCCAGTCGGGAAACCTGTCGTGCCAGCTGCATTAATGAATCGGCCA
ACGCGCGGGGAGAGGCGGTTTGCGTATTGGGCGCCAGGGTGGTTTTTCTTTTCACCAGTGAG
ACGGGCAACAGCTGATTGCCCTTCACCGCCTGGCCCTGAGAGAGTTGCAGCAAGCGGTCCAC
GCTGGTTTGCCCCAGCAGGCGAAAATCCTGTTTGATGGTGGTTAACGGCGGGATATAACATG
AGCTGTCTTCGGTATCGTCGTATCCCACTACCGAGATGTCCGCACCAACGCGCAGCCCGGAC
TCGGTAATGGCGCGCATTGCGCCCAGCGCCATCTGATCGTTGGCAACCAGCATCGCAGTGGG
AACGATGCCCTCATTCAGCATTTGCATGGTTTGTTGAAAACCGGACATGGCACTCCAGTCGCC
TTCCCGTTCCGCTATCGGCTGAATTTGATTGCGAGTGAGATATTTATGCCAGCCAGCCAGACG
CAGACGCGCCGAGACAGAACTTAATGGGCCCGCTAACAGCGCGATTTGCTGGTGACCCAATG
CGACCAGATGCTCCACGCCCAGTCGCGTACCGTCTTCATGGGAGAAAATAATACTGTTGATG
GGTGTCTGGTCAGAGACATCAAGAAATAACGCCGGAACATTAGTGCAGGCAGCTTCCACAGC
AATGGCATCCTGGTCATCCAGCGGATAGTTAATGATCAGCCCACTGACGCGTTGCGCGAGAA
GATTGTGCACCGCCGCTTTACAGGCTTCGACGCCGCTTCGTTCTACCATCGACACCACCACGC
TGGCACCCAGTTGATCGGCGCGAGATTTAATCGCCGCGACAATTTGCGACGGCGCGTGCAGG
GCCAGACTGGAGGTGGCAACGCCAATCAGCAACGACTGTTTGCCCGCCAGTTGTTGTGCCAC
GCGGTTGGGAATGTAATTCAGCTCCGCCATCGCCGCTTCCACTTTTTCCCGCGTTTTCGCAGA
AACGTGGCTGGCCTGGTTCACCACGCGGGAAACGGTCTGATAAGAGACACCGGCATACTCTG
CGACATCGTATAACGTTACTGGTTTCACATTCACCACCCTGAATTGACTCTCTTCCGGGCGCT
ATCATGCCATACCGCGAAAGGTTTTGCGCCATTCGATGGTGTCCGGGATCTCGACGCTCTCCC
TTATGCGACTCCTGCATTAGGAAATTAATACGACTCACTATA

n. pDuet::DsbA

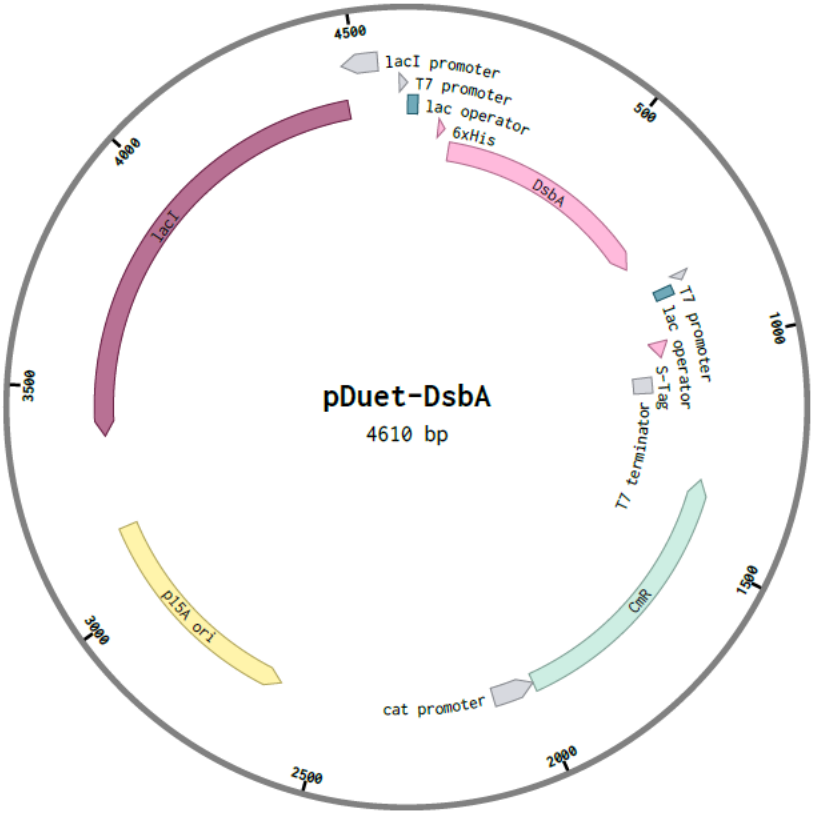

GGGGAATTGTGAGCGGATAACAATTCCCCTGTAGAAATAATTTTGTTTAACTTTAATAAGGA
GATATACCATGGGCAGCAGCCATCACCATCATCACCACAGCCAGGATCCGAATTCATGAAAA
AGATTTGGCTGGCGCTGGCTGGTTTAGTTTTAGCGTTTAGCGCATCGGCGGCGCAGTATGAA
GATGGTAAACAGTACACTACCCTGGAAAAACCGGTAGCTGGCGCGCCGCAAGTGCTGGAGTT
TTTCTCTTTCTTCTGCCCGCACTGCTATCAGTTTGAAGAAGTTCTGCATATTTCTGATAATGTG
AAGAAAAAACTGCCGGAAGGCGTGAAGATGACTAAATACCACGTCAACTTCATGGGTGGTG
ACCTGGGCAAAGATCTGACTCAGGCATGGGCTGTGGCGATGGCGCTGGGCGTGGAAGACAA
AGTGACTGTTCCGCTGTTTGAAGGCGTACAGAAAACCCAGACCATTCGTTCTGCTTCTGATAT
CCGCGATGTATTTATCAACGCAGGTATTAAAGGTGAAGAGTACGACGCGGCGTGGAACAGCT
TCGTGGTGAAATCTCTGGTCGCTCAGCAGGAAAAAGCTGCAGCTGACGTGCAATTGCGTGGC
GTTCCGGCGATGTTTGTTAACGGTAAATATCAGCTGAATCCGCAGGGTATGGATACCAGCAA
TATGGATGTTTTTGTTCAGCAGTATGCTGATACAGTGAAATATCTGTCCGAGAAAAAATAAA
AGCTTGCGGCCGCATAATGCTTAAGTCGAACAGAAAGTAATCGTATTGTACACGGCCGCATA
ATCGAAATTAATACGACTCACTATAGGGGAATTGTGAGCGGATAACAATTCCCCATCTTAGT
ATATTAGTTAAGTATAAGAAGGAGATATACATATGGCAGATCTCAATTGGATATCGGCCGGC
CACGCGATCGCTGACGTCGGTACCCTCGAGTCTGGTAAAGAAACCGCTGCTGCGAAATTTGA

ACGCCAGCACATGGACTCGTCTACTAGCGCAGCTTAATTAACCTAGGCTGCTGCCACCGCTG
AGCAATAACTAGCATAACCCCTTGGGGCCTCTAAACGGGTCTTGAGGGGTTTTTTGCTGAAA
CCTCAGGCATTTGAGAAGCACACGGTCACACTGCTTCCGGTAGTCAATAAACCGGTAAACCA
GCAATAGACATAAGCGGCTATTTAACGACCCTGCCCTGAACCGACGACCGGGTCGAATTTGC
TTTCGAATTTCTGCCATTCATCCGCTTATTATCACTTATTCAGGCGTAGCACCAGGCGTTTAA
GGGCACCAATAACTGCCTTAAAAAAATTACGCCCCGCCCTGCCACTCATCGCAGTACTGTTG
TAATTCATTAAGCATTCTGCCGACATGGAAGCCATCACAGACGGCATGATGAACCTGAATCG
CCAGCGGCATCAGCACCTTGTCGCCTTGCGTATAATATTTGCCCATAGTGAAAACGGGGGCG
AAGAAGTTGTCCATATTGGCCACGTTTAAATCAAAACTGGTGAAACTCACCCAGGGATTGGC
TGAGACGAAAAACATATTCTCAATAAACCCTTTAGGGAAATAGGCCAGGTTTTCACCGTAAC
ACGCCACATCTTGCGAATATATGTGTAGAAACTGCCGGAAATCGTCGTGGTATTCACTCCAG
AGCGATGAAAACGTTTCAGTTTGCTCATGGAAAACGGTGTAACAAGGGTGAACACTATCCCA
TATCACCAGCTCACCGTCTTTCATTGCCATACGGAACTCCGGATGAGCATTCATCAGGCGGGC
AAGAATGTGAATAAAGGCCGGATAAAACTTGTGCTTATTTTTCTTTACGGTCTTTAAAAAGGC
CGTAATATCCAGCTGAACGGTCTGGTTATAGGTACATTGAGCAACTGACTGAAATGCCTCAA
AATGTTCTTTACGATGCCATTGGGATATATCAACGGTGGTATATCCAGTGATTTTTTTCTCCAT
TTTAGCTTCCTTAGCTCCTGAAAATCTCGATAACTCAAAAAATACGCCCGGTAGTGATCTTAT
TTCATTATGGTGAAAGTTGGAACCTCTTACGTGCCGATCAACGTCTCATTTTCGCCAAAAGTT
GGCCCAGGGCTTCCCGGTATCAACAGGGACACCAGGATTTATTTATTCTGCGAAGTGATCTTC
CGTCACAGGTATTTATTCGGCGCAAAGTGCGTCGGGTGATGCTGCCAACTTACTGATTTAGTG
TATGATGGTGTTTTTGAGGTGCTCCAGTGGCTTCTGTTTCTATCAGCTGTCCCTCCTGTTCAGC
TACTGACGGGGTGGTGCGTAACGGCAAAAGCACCGCCGGACATCAGCGCTAGCGGAGTGTA
TACTGGCTTACTATGTTGGCACTGATGAGGGTGTCAGTGAAGTGCTTCATGTGGCAGGAGAA
AAAAGGCTGCACCGGTGCGTCAGCAGAATATGTGATACAGGATATATTCCGCTTCCTCGCTC
ACTGACTCGCTACGCTCGGTCGTTCGACTGCGGCGAGCGGAAATGGCTTACGAACGGGGCGG
AGATTTCCTGGAAGATGCCAGGAAGATACTTAACAGGGAAGTGAGAGGGCCGCGGCAAAGC
CGTTTTTCCATAGGCTCCGCCCCCCTGACAAGCATCACGAAATCTGACGCTCAAATCAGTGGT
GGCGAAACCCGACAGGACTATAAAGATACCAGGCGTTTCCCCTGGCGGCTCCCTCGTGCGCT
CTCCTGTTCCTGCCTTTCGGTTTACCGGTGTCATTCCGCTGTTATGGCCGCGTTTGTCTCATTC
CACGCCTGACACTCAGTTCCGGGTAGGCAGTTCGCTCCAAGCTGGACTGTATGCACGAACCC
CCCGTTCAGTCCGACCGCTGCGCCTTATCCGGTAACTATCGTCTTGAGTCCAACCCGGAAAGA
CATGCAAAAGCACCACTGGCAGCAGCCACTGGTAATTGATTTAGAGGAGTTAGTCTTGAAGT
CATGCGCCGGTTAAGGCTAAACTGAAAGGACAAGTTTTGGTGACTGCGCTCCTCCAAGCCAG
TTACCTCGGTTCAAAGAGTTGGTAGCTCAGAGAACCTTCGAAAAACCGCCCTGCAAGGCGGT

TTTTTCGTTTTCAGAGCAAGAGATTACGCGCAGACCAAAACGATCTCAAGAAGATCATCTTA
TTAATCAGATAAAATATTTCTAGATTTCAGTGCAATTTATCTCTTCAAATGTAGCACCTGAAG
TCAGCCCCATACGATATAAGTTGTAATTCTCATGTTAGTCATGCCCCGCGCCCACCGGAAGG
AGCTGACTGGGTTGAAGGCTCTCAAGGGCATCGGTCGAGATCCCGGTGCCTAATGAGTGAGC
TAACTTACATTAATTGCGTTGCGCTCACTGCCCGCTTTCCAGTCGGGAAACCTGTCGTGCCAG
CTGCATTAATGAATCGGCCAACGCGCGGGGAGAGGCGGTTTGCGTATTGGGCGCCAGGGTGG
TTTTTCTTTTCACCAGTGAGACGGGCAACAGCTGATTGCCCTTCACCGCCTGGCCCTGAGAGA
GTTGCAGCAAGCGGTCCACGCTGGTTTGCCCCAGCAGGCGAAAATCCTGTTTGATGGTGGTT
AACGGCGGGATATAACATGAGCTGTCTTCGGTATCGTCGTATCCCACTACCGAGATGTCCGC
ACCAACGCGCAGCCCGGACTCGGTAATGGCGCGCATTGCGCCCAGCGCCATCTGATCGTTGG
CAACCAGCATCGCAGTGGGAACGATGCCCTCATTCAGCATTTGCATGGTTTGTTGAAAACCG
GACATGGCACTCCAGTCGCCTTCCCGTTCCGCTATCGGCTGAATTTGATTGCGAGTGAGATAT
TTATGCCAGCCAGCCAGACGCAGACGCGCCGAGACAGAACTTAATGGGCCCGCTAACAGCG
CGATTTGCTGGTGACCCAATGCGACCAGATGCTCCACGCCCAGTCGCGTACCGTCTTCATGG
GAGAAAATAATACTGTTGATGGGTGTCTGGTCAGAGACATCAAGAAATAACGCCGGAACATT
AGTGCAGGCAGCTTCCACAGCAATGGCATCCTGGTCATCCAGCGGATAGTTAATGATCAGCC
CACTGACGCGTTGCGCGAGAAGATTGTGCACCGCCGCTTTACAGGCTTCGACGCCGCTTCGTT
CTACCATCGACACCACCACGCTGGCACCCAGTTGATCGGCGCGAGATTTAATCGCCGCGACA
ATTTGCGACGGCGCGTGCAGGGCCAGACTGGAGGTGGCAACGCCAATCAGCAACGACTGTTT
GCCCGCCAGTTGTTGTGCCACGCGGTTGGGAATGTAATTCAGCTCCGCCATCGCCGCTTCCAC
TTTTTCCCGCGTTTTCGCAGAAACGTGGCTGGCCTGGTTCACCACGCGGGAAACGGTCTGATA
AGAGACACCGGCATACTCTGCGACATCGTATAACGTTACTGGTTTCACATTCACCACCCTGA
ATTGACTCTCTTCCGGGCGCTATCATGCCATACCGCGAAAGGTTTTGCGCCATTCGATGGTGT
CCGGGATCTCGACGCTCTCCCTTATGCGACTCCTGCATTAGGAAATTAATACGACTCACTATA

o. pDuet::DsbB

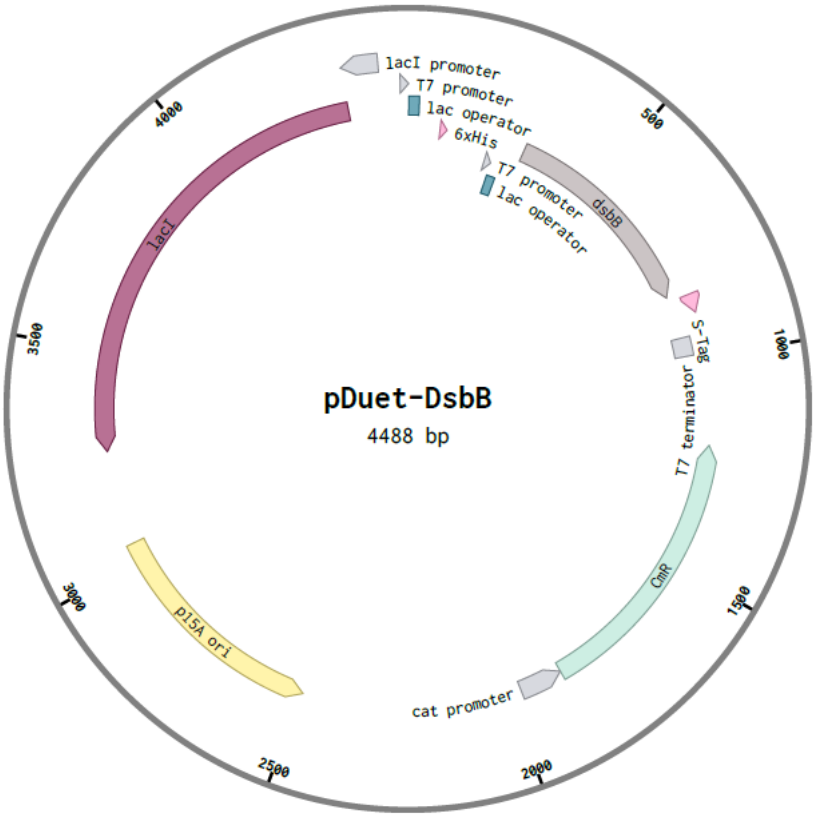

GGGGAATTGTGAGCGGATAACAATTCCCCTGTAGAAATAATTTTGTTTAACTTTAATAAGGA
GATATACCATGGGCAGCAGCCATCACCATCATCACCACAGCCAGGATCCGAATTCGAGCTCG
GCGCGCCTGCAGGTCGACAAGCTTGCGGCCGCATAATGCTTAAGTCGAACAGAAAGTAATCG
TATTGTACACGGCCGCATAATCGAAATTAATACGACTCACTATAGGGGAATTGTGAGCGGAT
AACAATTCCCCATCTTAGTATATTAGTTAAGTATAAGAAGGAGATATACATATGATGTTGCG
ATTTTTGAACCAATGTTCACAAGGCCGGGGCGCGTGGCTGTTGATGGCGTTTACTGCTCTGGC
ACTGGAACTGACGGCGCTGTGGTTCCAGCATGTGATGTTACTGAAACCTTGCGTGCTCTGTAT
TTATGAACGCTGCGCGTTATTCGGCGTTCTGGGTGCTGCGCTGATTGGCGCGATCGCCCCGAA
AACTCCGCTGCGTTATGTAGCGATGGTTATCTGGTTGTATAGTGCGTTCCGCGGTGTGCAGTT
AACTTACGAGCACACCATGCTTCAGCTCTATCCTTCGCCGTTTGCCACCTGTGATTTTATGGTT
CGTTTCCCGGAATGGCTGCCGCTGGATAAGTGGGTGCCGCAAGTGTTTGTCGCCTCTGGCGAT
TGCGCCGAGCGTCAGTGGGATTTTTTAGGTCTGGAAATGCCGCAGTGGCTGCTCGGTATTTTT
ATCGCTTACCTGATTGTCGCAGTGCTGGTGGTGATTTCCCAGCCGTTTAAAGCGAAAAAACGT
GATCTGTTCGGTCGCTAACTCGAGTCTGGTAAAGAAACCGCTGCTGCGAAATTTGAACGCCA
GCACATGGACTCGTCTACTAGCGCAGCTTAATTAACCTAGGCTGCTGCCACCGCTGAGCAAT
AACTAGCATAACCCCTTGGGGCCTCTAAACGGGTCTTGAGGGGTTTTTTGCTGAAACCTCAG

GCATTTGAGAAGCACACGGTCACACTGCTTCCGGTAGTCAATAAACCGGTAAACCAGCAATA
GACATAAGCGGCTATTTAACGACCCTGCCCTGAACCGACGACCGGGTCGAATTTGCTTTCGA
ATTTCTGCCATTCATCCGCTTATTATCACTTATTCAGGCGTAGCACCAGGCGTTTAAGGGCAC
CAATAACTGCCTTAAAAAAATTACGCCCCGCCCTGCCACTCATCGCAGTACTGTTGTAATTCA
TTAAGCATTCTGCCGACATGGAAGCCATCACAGACGGCATGATGAACCTGAATCGCCAGCGG
CATCAGCACCTTGTCGCCTTGCGTATAATATTTGCCCATAGTGAAAACGGGGGCGAAGAAGT
TGTCCATATTGGCCACGTTTAAATCAAAACTGGTGAAACTCACCCAGGGATTGGCTGAGACG
AAAAACATATTCTCAATAAACCCTTTAGGGAAATAGGCCAGGTTTTCACCGTAACACGCCAC
ATCTTGCGAATATATGTGTAGAAACTGCCGGAAATCGTCGTGGTATTCACTCCAGAGCGATG
AAAACGTTTCAGTTTGCTCATGGAAAACGGTGTAACAAGGGTGAACACTATCCCATATCACC
AGCTCACCGTCTTTCATTGCCATACGGAACTCCGGATGAGCATTCATCAGGCGGGCAAGAAT
GTGAATAAAGGCCGGATAAAACTTGTGCTTATTTTTCTTTACGGTCTTTAAAAAGGCCGTAAT
ATCCAGCTGAACGGTCTGGTTATAGGTACATTGAGCAACTGACTGAAATGCCTCAAAATGTT
CTTTACGATGCCATTGGGATATATCAACGGTGGTATATCCAGTGATTTTTTTCTCCATTTTAGC
TTCCTTAGCTCCTGAAAATCTCGATAACTCAAAAAATACGCCCGGTAGTGATCTTATTTCATT
ATGGTGAAAGTTGGAACCTCTTACGTGCCGATCAACGTCTCATTTTCGCCAAAAGTTGGCCCA
GGGCTTCCCGGTATCAACAGGGACACCAGGATTTATTTATTCTGCGAAGTGATCTTCCGTCAC
AGGTATTTATTCGGCGCAAAGTGCGTCGGGTGATGCTGCCAACTTACTGATTTAGTGTATGAT
GGTGTTTTTGAGGTGCTCCAGTGGCTTCTGTTTCTATCAGCTGTCCCTCCTGTTCAGCTACTGA
CGGGGTGGTGCGTAACGGCAAAAGCACCGCCGGACATCAGCGCTAGCGGAGTGTATACTGG
CTTACTATGTTGGCACTGATGAGGGTGTCAGTGAAGTGCTTCATGTGGCAGGAGAAAAAAGG
CTGCACCGGTGCGTCAGCAGAATATGTGATACAGGATATATTCCGCTTCCTCGCTCACTGACT
CGCTACGCTCGGTCGTTCGACTGCGGCGAGCGGAAATGGCTTACGAACGGGGCGGAGATTTC
CTGGAAGATGCCAGGAAGATACTTAACAGGGAAGTGAGAGGGCCGCGGCAAAGCCGTTTTT
CCATAGGCTCCGCCCCCCTGACAAGCATCACGAAATCTGACGCTCAAATCAGTGGTGGCGAA
ACCCGACAGGACTATAAAGATACCAGGCGTTTCCCCTGGCGGCTCCCTCGTGCGCTCTCCTGT
TCCTGCCTTTCGGTTTACCGGTGTCATTCCGCTGTTATGGCCGCGTTTGTCTCATTCCACGCCT
GACACTCAGTTCCGGGTAGGCAGTTCGCTCCAAGCTGGACTGTATGCACGAACCCCCCGTTC
AGTCCGACCGCTGCGCCTTATCCGGTAACTATCGTCTTGAGTCCAACCCGGAAAGACATGCA
AAAGCACCACTGGCAGCAGCCACTGGTAATTGATTTAGAGGAGTTAGTCTTGAAGTCATGCG
CCGGTTAAGGCTAAACTGAAAGGACAAGTTTTGGTGACTGCGCTCCTCCAAGCCAGTTACCT
CGGTTCAAAGAGTTGGTAGCTCAGAGAACCTTCGAAAAACCGCCCTGCAAGGCGGTTTTTTC
GTTTTCAGAGCAAGAGATTACGCGCAGACCAAAACGATCTCAAGAAGATCATCTTATTAATC
AGATAAAATATTTCTAGATTTCAGTGCAATTTATCTCTTCAAATGTAGCACCTGAAGTCAGCC

CCATACGATATAAGTTGTAATTCTCATGTTAGTCATGCCCCGCGCCCACCGGAAGGAGCTGA
CTGGGTTGAAGGCTCTCAAGGGCATCGGTCGAGATCCCGGTGCCTAATGAGTGAGCTAACTT
ACATTAATTGCGTTGCGCTCACTGCCCGCTTTCCAGTCGGGAAACCTGTCGTGCCAGCTGCAT
TAATGAATCGGCCAACGCGCGGGGAGAGGCGGTTTGCGTATTGGGCGCCAGGGTGGTTTTTC
TTTTCACCAGTGAGACGGGCAACAGCTGATTGCCCTTCACCGCCTGGCCCTGAGAGAGTTGC
AGCAAGCGGTCCACGCTGGTTTGCCCCAGCAGGCGAAAATCCTGTTTGATGGTGGTTAACGG
CGGGATATAACATGAGCTGTCTTCGGTATCGTCGTATCCCACTACCGAGATGTCCGCACCAA
CGCGCAGCCCGGACTCGGTAATGGCGCGCATTGCGCCCAGCGCCATCTGATCGTTGGCAACC
AGCATCGCAGTGGGAACGATGCCCTCATTCAGCATTTGCATGGTTTGTTGAAAACCGGACAT
GGCACTCCAGTCGCCTTCCCGTTCCGCTATCGGCTGAATTTGATTGCGAGTGAGATATTTATG
CCAGCCAGCCAGACGCAGACGCGCCGAGACAGAACTTAATGGGCCCGCTAACAGCGCGATT
TGCTGGTGACCCAATGCGACCAGATGCTCCACGCCCAGTCGCGTACCGTCTTCATGGGAGAA
AATAATACTGTTGATGGGTGTCTGGTCAGAGACATCAAGAAATAACGCCGGAACATTAGTGC
AGGCAGCTTCCACAGCAATGGCATCCTGGTCATCCAGCGGATAGTTAATGATCAGCCCACTG
ACGCGTTGCGCGAGAAGATTGTGCACCGCCGCTTTACAGGCTTCGACGCCGCTTCGTTCTACC
ATCGACACCACCACGCTGGCACCCAGTTGATCGGCGCGAGATTTAATCGCCGCGACAATTTG
CGACGGCGCGTGCAGGGCCAGACTGGAGGTGGCAACGCCAATCAGCAACGACTGTTTGCCC
GCCAGTTGTTGTGCCACGCGGTTGGGAATGTAATTCAGCTCCGCCATCGCCGCTTCCACTTTT
TCCCGCGTTTTCGCAGAAACGTGGCTGGCCTGGTTCACCACGCGGGAAACGGTCTGATAAGA
GACACCGGCATACTCTGCGACATCGTATAACGTTACTGGTTTCACATTCACCACCCTGAATTG
ACTCTCTTCCGGGCGCTATCATGCCATACCGCGAAAGGTTTTGCGCCATTCGATGGTGTCCGG
GATCTCGACGCTCTCCCTTATGCGACTCCTGCATTAGGAAATTAATACGACTCACTATA

**p.	pDuet::DsbAB**

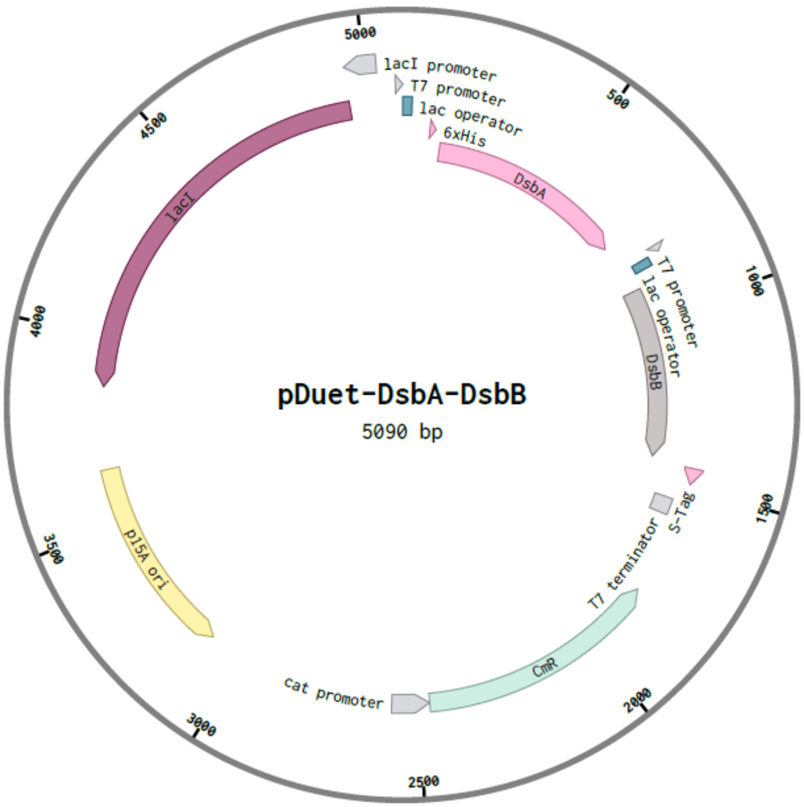

GGGGAATTGTGAGCGGATAACAATTCCCCTGTAGAAATAATTTTGTTTAACTTTAATAAGGA
GATATACCATGGGCAGCAGCCATCACCATCATCACCACAGCCAGGATCCGAATTCATGAAAA
AGATTTGGCTGGCGCTGGCTGGTTTAGTTTTAGCGTTTAGCGCATCGGCGGCGCAGTATGAA
GATGGTAAACAGTACACTACCCTGGAAAAACCGGTAGCTGGCGCGCCGCAAGTGCTGGAGTT
TTTCTCTTTCTTCTGCCCGCACTGCTATCAGTTTGAAGAAGTTCTGCATATTTCTGATAATGTG
AAGAAAAAACTGCCGGAAGGCGTGAAGATGACTAAATACCACGTCAACTTCATGGGTGGTG
ACCTGGGCAAAGATCTGACTCAGGCATGGGCTGTGGCGATGGCGCTGGGCGTGGAAGACAA
AGTGACTGTTCCGCTGTTTGAAGGCGTACAGAAAACCCAGACCATTCGTTCTGCTTCTGATAT
CCGCGATGTATTTATCAACGCAGGTATTAAAGGTGAAGAGTACGACGCGGCGTGGAACAGCT
TCGTGGTGAAATCTCTGGTCGCTCAGCAGGAAAAAGCTGCAGCTGACGTGCAATTGCGTGGC
GTTCCGGCGATGTTTGTTAACGGTAAATATCAGCTGAATCCGCAGGGTATGGATACCAGCAA
TATGGATGTTTTTGTTCAGCAGTATGCTGATACAGTGAAATATCTGTCCGAGAAAAAATAAA
AGCTTGCGGCCGCATAATGCTTAAGTCGAACAGAAAGTAATCGTATTGTACACGGCCGCATA
ATCGAAATTAATACGACTCACTATAGGGGAATTGTGAGCGGATAACAATTCCCCATCTTAGT
ATATTAGTTAAGTATAAGAAGGAGATATACATATGATGTTGCGATTTTTGAACCAATGTTCAC
AAGGCCGGGGCGCGTGGCTGTTGATGGCGTTTACTGCTCTGGCACTGGAACTGACGGCGCTG

TGGTTCCAGCATGTGATGTTACTGAAACCTTGCGTGCTCTGTATTTATGAACGCTGCGCGTTA
TTCGGCGTTCTGGGTGCTGCGCTGATTGGCGCGATCGCCCCGAAAACTCCGCTGCGTTATGTA
GCGATGGTTATCTGGTTGTATAGTGCGTTCCGCGGTGTGCAGTTAACTTACGAGCACACCATG
CTTCAGCTCTATCCTTCGCCGTTTGCCACCTGTGATTTTATGGTTCGTTTCCCGGAATGGCTGC
CGCTGGATAAGTGGGTGCCGCAAGTGTTTGTCGCCTCTGGCGATTGCGCCGAGCGTCAGTGG
GATTTTTTAGGTCTGGAAATGCCGCAGTGGCTGCTCGGTATTTTTATCGCTTACCTGATTGTC
GCAGTGCTGGTGGTGATTTCCCAGCCGTTTAAAGCGAAAAAACGTGATCTGTTCGGTCGCTA
ACTCGAGTCTGGTAAAGAAACCGCTGCTGCGAAATTTGAACGCCAGCACATGGACTCGTCTA
CTAGCGCAGCTTAATTAACCTAGGCTGCTGCCACCGCTGAGCAATAACTAGCATAACCCCTT
GGGGCCTCTAAACGGGTCTTGAGGGGTTTTTTGCTGAAACCTCAGGCATTTGAGAAGCACAC
GGTCACACTGCTTCCGGTAGTCAATAAACCGGTAAACCAGCAATAGACATAAGCGGCTATTT
AACGACCCTGCCCTGAACCGACGACCGGGTCGAATTTGCTTTCGAATTTCTGCCATTCATCCG
CTTATTATCACTTATTCAGGCGTAGCACCAGGCGTTTAAGGGCACCAATAACTGCCTTAAAA
AAATTACGCCCCGCCCTGCCACTCATCGCAGTACTGTTGTAATTCATTAAGCATTCTGCCGAC
ATGGAAGCCATCACAGACGGCATGATGAACCTGAATCGCCAGCGGCATCAGCACCTTGTCGC
CTTGCGTATAATATTTGCCCATAGTGAAAACGGGGGCGAAGAAGTTGTCCATATTGGCCACG
TTTAAATCAAAACTGGTGAAACTCACCCAGGGATTGGCTGAGACGAAAAACATATTCTCAAT
AAACCCTTTAGGGAAATAGGCCAGGTTTTCACCGTAACACGCCACATCTTGCGAATATATGT
GTAGAAACTGCCGGAAATCGTCGTGGTATTCACTCCAGAGCGATGAAAACGTTTCAGTTTGC
TCATGGAAAACGGTGTAACAAGGGTGAACACTATCCCATATCACCAGCTCACCGTCTTTCAT
TGCCATACGGAACTCCGGATGAGCATTCATCAGGCGGGCAAGAATGTGAATAAAGGCCGGA
TAAAACTTGTGCTTATTTTTCTTTACGGTCTTTAAAAAGGCCGTAATATCCAGCTGAACGGTC
TGGTTATAGGTACATTGAGCAACTGACTGAAATGCCTCAAAATGTTCTTTACGATGCCATTGG
GATATATCAACGGTGGTATATCCAGTGATTTTTTTCTCCATTTTAGCTTCCTTAGCTCCTGAAA
ATCTCGATAACTCAAAAAATACGCCCGGTAGTGATCTTATTTCATTATGGTGAAAGTTGGAA
CCTCTTACGTGCCGATCAACGTCTCATTTTCGCCAAAAGTTGGCCCAGGGCTTCCCGGTATCA
ACAGGGACACCAGGATTTATTTATTCTGCGAAGTGATCTTCCGTCACAGGTATTTATTCGGCG
CAAAGTGCGTCGGGTGATGCTGCCAACTTACTGATTTAGTGTATGATGGTGTTTTTGAGGTGC
TCCAGTGGCTTCTGTTTCTATCAGCTGTCCCTCCTGTTCAGCTACTGACGGGGTGGTGCGTAA
CGGCAAAAGCACCGCCGGACATCAGCGCTAGCGGAGTGTATACTGGCTTACTATGTTGGCAC
TGATGAGGGTGTCAGTGAAGTGCTTCATGTGGCAGGAGAAAAAAGGCTGCACCGGTGCGTC
AGCAGAATATGTGATACAGGATATATTCCGCTTCCTCGCTCACTGACTCGCTACGCTCGGTCG
TTCGACTGCGGCGAGCGGAAATGGCTTACGAACGGGGCGGAGATTTCCTGGAAGATGCCAG
GAAGATACTTAACAGGGAAGTGAGAGGGCCGCGGCAAAGCCGTTTTTCCATAGGCTCCGCCC

CCCTGACAAGCATCACGAAATCTGACGCTCAAATCAGTGGTGGCGAAACCCGACAGGACTAT
AAAGATACCAGGCGTTTCCCCTGGCGGCTCCCTCGTGCGCTCTCCTGTTCCTGCCTTTCGGTTT
ACCGGTGTCATTCCGCTGTTATGGCCGCGTTTGTCTCATTCCACGCCTGACACTCAGTTCCGG
GTAGGCAGTTCGCTCCAAGCTGGACTGTATGCACGAACCCCCCGTTCAGTCCGACCGCTGCG
CCTTATCCGGTAACTATCGTCTTGAGTCCAACCCGGAAAGACATGCAAAAGCACCACTGGCA
GCAGCCACTGGTAATTGATTTAGAGGAGTTAGTCTTGAAGTCATGCGCCGGTTAAGGCTAAA
CTGAAAGGACAAGTTTTGGTGACTGCGCTCCTCCAAGCCAGTTACCTCGGTTCAAAGAGTTG
GTAGCTCAGAGAACCTTCGAAAAACCGCCCTGCAAGGCGGTTTTTTCGTTTTCAGAGCAAGA
GATTACGCGCAGACCAAAACGATCTCAAGAAGATCATCTTATTAATCAGATAAAATATTTCT
AGATTTCAGTGCAATTTATCTCTTCAAATGTAGCACCTGAAGTCAGCCCCATACGATATAAGT
TGTAATTCTCATGTTAGTCATGCCCCGCGCCCACCGGAAGGAGCTGACTGGGTTGAAGGCTC
TCAAGGGCATCGGTCGAGATCCCGGTGCCTAATGAGTGAGCTAACTTACATTAATTGCGTTG
CGCTCACTGCCCGCTTTCCAGTCGGGAAACCTGTCGTGCCAGCTGCATTAATGAATCGGCCA
ACGCGCGGGGAGAGGCGGTTTGCGTATTGGGCGCCAGGGTGGTTTTTCTTTTCACCAGTGAG
ACGGGCAACAGCTGATTGCCCTTCACCGCCTGGCCCTGAGAGAGTTGCAGCAAGCGGTCCAC
GCTGGTTTGCCCCAGCAGGCGAAAATCCTGTTTGATGGTGGTTAACGGCGGGATATAACATG
AGCTGTCTTCGGTATCGTCGTATCCCACTACCGAGATGTCCGCACCAACGCGCAGCCCGGAC
TCGGTAATGGCGCGCATTGCGCCCAGCGCCATCTGATCGTTGGCAACCAGCATCGCAGTGGG
AACGATGCCCTCATTCAGCATTTGCATGGTTTGTTGAAAACCGGACATGGCACTCCAGTCGCC
TTCCCGTTCCGCTATCGGCTGAATTTGATTGCGAGTGAGATATTTATGCCAGCCAGCCAGACG
CAGACGCGCCGAGACAGAACTTAATGGGCCCGCTAACAGCGCGATTTGCTGGTGACCCAATG
CGACCAGATGCTCCACGCCCAGTCGCGTACCGTCTTCATGGGAGAAAATAATACTGTTGATG
GGTGTCTGGTCAGAGACATCAAGAAATAACGCCGGAACATTAGTGCAGGCAGCTTCCACAGC
AATGGCATCCTGGTCATCCAGCGGATAGTTAATGATCAGCCCACTGACGCGTTGCGCGAGAA
GATTGTGCACCGCCGCTTTACAGGCTTCGACGCCGCTTCGTTCTACCATCGACACCACCACGC
TGGCACCCAGTTGATCGGCGCGAGATTTAATCGCCGCGACAATTTGCGACGGCGCGTGCAGG
GCCAGACTGGAGGTGGCAACGCCAATCAGCAACGACTGTTTGCCCGCCAGTTGTTGTGCCAC
GCGGTTGGGAATGTAATTCAGCTCCGCCATCGCCGCTTCCACTTTTTCCCGCGTTTTCGCAGA
AACGTGGCTGGCCTGGTTCACCACGCGGGAAACGGTCTGATAAGAGACACCGGCATACTCTG
CGACATCGTATAACGTTACTGGTTTCACATTCACCACCCTGAATTGACTCTCTTCCGGGCGCT
ATCATGCCATACCGCGAAAGGTTTTGCGCCATTCGATGGTGTCCGGGATCTCGACGCTCTCCC
TTATGCGACTCCTGCATTAGGAAATTAATACGACTCACTATA

q. pDuet::GrpE

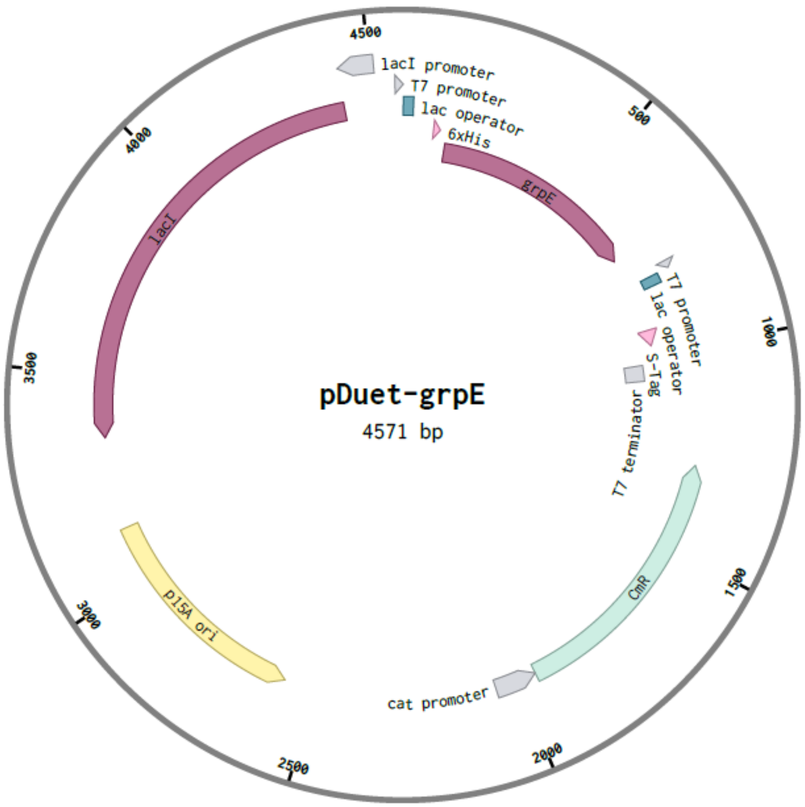

GGGGAATTGTGAGCGGATAACAATTCCCCTGTAGAAATAATTTTGTTTAACTTTAATAAGGA
GATATACCATGGGCAGCAGCCATCACCATCATCACCACAGCCAGGATCCGAATTCATGAGTA
GTAAAGAACAGAAAACGCCTGAGGGGCAAGCCCCGGAAGAAATTATCATGGATCAGCACGA
AGAGATTGAGGCAGTTGAGCCAGAAGCTTCTGCTGAGCAGGTGGATCCGCGCGATGAAAAA
GTTGCGAATCTCGAAGCTCAGCTGGCTGAAGCCCAGACCCGTGAACGTGACGGCATTTTGCG
TGTAAAAGCCGAAATGGAAAACCTGCGTCGTCGTACTGAACTGGATATTGAAAAAGCCCACA
AATTCGCGCTGGAGAAATTCATCAACGAATTGCTGCCGGTGATTGATAGCCTGGATCGTGCG
CTGGAAGTGGCTGATAAAGCTAACCCGGATATGTCTGCGATGGTTGAAGGCATTGAGCTGAC
GCTGAAGTCGATGCTGGATGTTGTGCGTAAGTTTGGCGTTGAAGTGATCGCCGAAACTAACG
TCCCACTGGACCCGAATGTGCATCAGGCCATCGCAATGGTGGAATCTGATGACGTTGCGCCA
GGTAACGTACTGGGCATTATGCAGAAGGGTTATACGCTGAATGGTCGTACGATTCGTGCGGC
GATGGTTACTGTAGCGAAAGCAAAAGCTTAAGCGGCCGCATAATGCTTAAGTCGAACAGAA
AGTAATCGTATTGTACACGGCCGCATAATCGAAATTAATACGACTCACTATAGGGGAATTGT
GAGCGGATAACAATTCCCCATCTTAGTATATTAGTTAAGTATAAGAAGGAGATATACATATG
GCAGATCTCAATTGGATATCGGCCGGCCACGCGATCGCTGACGTCGGTACCCTCGAGTCTGG
TAAAGAAACCGCTGCTGCGAAATTTGAACGCCAGCACATGGACTCGTCTACTAGCGCAGCTT

AATTAACCTAGGCTGCTGCCACCGCTGAGCAATAACTAGCATAACCCCTTGGGGCCTCTAAA
CGGGTCTTGAGGGGTTTTTTGCTGAAACCTCAGGCATTTGAGAAGCACACGGTCACACTGCTT
CCGGTAGTCAATAAACCGGTAAACCAGCAATAGACATAAGCGGCTATTTAACGACCCTGCCC
TGAACCGACGACCGGGTCGAATTTGCTTTCGAATTTCTGCCATTCATCCGCTTATTATCACTT
ATTCAGGCGTAGCACCAGGCGTTTAAGGGCACCAATAACTGCCTTAAAAAAATTACGCCCCG
CCCTGCCACTCATCGCAGTACTGTTGTAATTCATTAAGCATTCTGCCGACATGGAAGCCATCA
CAGACGGCATGATGAACCTGAATCGCCAGCGGCATCAGCACCTTGTCGCCTTGCGTATAATA
TTTGCCCATAGTGAAAACGGGGGCGAAGAAGTTGTCCATATTGGCCACGTTTAAATCAAAAC
TGGTGAAACTCACCCAGGGATTGGCTGAGACGAAAAACATATTCTCAATAAACCCTTTAGGG
AAATAGGCCAGGTTTTCACCGTAACACGCCACATCTTGCGAATATATGTGTAGAAACTGCCG
GAAATCGTCGTGGTATTCACTCCAGAGCGATGAAAACGTTTCAGTTTGCTCATGGAAAACGG
TGTAACAAGGGTGAACACTATCCCATATCACCAGCTCACCGTCTTTCATTGCCATACGGAACT
CCGGATGAGCATTCATCAGGCGGGCAAGAATGTGAATAAAGGCCGGATAAAACTTGTGCTTA
TTTTTCTTTACGGTCTTTAAAAAGGCCGTAATATCCAGCTGAACGGTCTGGTTATAGGTACAT
TGAGCAACTGACTGAAATGCCTCAAAATGTTCTTTACGATGCCATTGGGATATATCAACGGT
GGTATATCCAGTGATTTTTTTCTCCATTTTAGCTTCCTTAGCTCCTGAAAATCTCGATAACTCA
AAAAATACGCCCGGTAGTGATCTTATTTCATTATGGTGAAAGTTGGAACCTCTTACGTGCCGA
TCAACGTCTCATTTTCGCCAAAAGTTGGCCCAGGGCTTCCCGGTATCAACAGGGACACCAGG
ATTTATTTATTCTGCGAAGTGATCTTCCGTCACAGGTATTTATTCGGCGCAAAGTGCGTCGGG
TGATGCTGCCAACTTACTGATTTAGTGTATGATGGTGTTTTTGAGGTGCTCCAGTGGCTTCTG
TTTCTATCAGCTGTCCCTCCTGTTCAGCTACTGACGGGGTGGTGCGTAACGGCAAAAGCACCG
CCGGACATCAGCGCTAGCGGAGTGTATACTGGCTTACTATGTTGGCACTGATGAGGGTGTCA
GTGAAGTGCTTCATGTGGCAGGAGAAAAAAGGCTGCACCGGTGCGTCAGCAGAATATGTGA
TACAGGATATATTCCGCTTCCTCGCTCACTGACTCGCTACGCTCGGTCGTTCGACTGCGGCGA
GCGGAAATGGCTTACGAACGGGGCGGAGATTTCCTGGAAGATGCCAGGAAGATACTTAACA
GGGAAGTGAGAGGGCCGCGGCAAAGCCGTTTTTCCATAGGCTCCGCCCCCCTGACAAGCATC
ACGAAATCTGACGCTCAAATCAGTGGTGGCGAAACCCGACAGGACTATAAAGATACCAGGC
GTTTCCCCCTGGCGGCTCCCTCGTGCGCTCTCCTGTTCCTGCCTTTCGGTTTACCGGTGTCATTC
CGCTGTTATGGCCGCGTTTGTCTCATTCCACGCCTGACACTCAGTTCCGGGTAGGCAGTTCGC
TCCAAGCTGGACTGTATGCACGAACCCCCCGTTCAGTCCGACCGCTGCGCCTTATCCGGTAAC
TATCGTCTTGAGTCCAACCCGGAAAGACATGCAAAAGCACCACTGGCAGCAGCCACTGGTAA
TTGATTTAGAGGAGTTAGTCTTGAAGTCATGCGCCGGTTAAGGCTAAACTGAAAGGACAAGT
TTTGGTGACTGCGCTCCTCCAAGCCAGTTACCTCGGTTCAAAGAGTTGGTAGCTCAGAGAAC
CTTCGAAAAACCGCCCTGCAAGGCGGTTTTTTCGTTTTCAGAGCAAGAGATTACGCGCAGAC

CAAAACGATCTCAAGAAGATCATCTTATTAATCAGATAAAATATTTCTAGATTTCAGTGCAAT
TTATCTCTTCAAATGTAGCACCTGAAGTCAGCCCCATACGATATAAGTTGTAATTCTCATGTT
AGTCATGCCCCGCGCCCACCGGAAGGAGCTGACTGGGTTGAAGGCTCTCAAGGGCATCGGTC
GAGATCCCGGTGCCTAATGAGTGAGCTAACTTACATTAATTGCGTTGCGCTCACTGCCCGCTT
TCCAGTCGGGAAACCTGTCGTGCCAGCTGCATTAATGAATCGGCCAACGCGCGGGGAGAGGC
GGTTTGCGTATTGGGCGCCAGGGTGGTTTTTCTTTTCACCAGTGAGACGGGCAACAGCTGATT
GCCCTTCACCGCCTGGCCCTGAGAGAGTTGCAGCAAGCGGTCCACGCTGGTTTGCCCCAGCA
GGCGAAAATCCTGTTTGATGGTGGTTAACGGCGGGATATAACATGAGCTGTCTTCGGTATCG
TCGTATCCCACTACCGAGATGTCCGCACCAACGCGCAGCCCGGACTCGGTAATGGCGCGCAT
TGCGCCCAGCGCCATCTGATCGTTGGCAACCAGCATCGCAGTGGGAACGATGCCCTCATTCA
GCATTTGCATGGTTTGTTGAAAACCGGACATGGCACTCCAGTCGCCTTCCCGTTCCGCTATCG
GCTGAATTTGATTGCGAGTGAGATATTTATGCCAGCCAGCCAGACGCAGACGCGCCGAGACA
GAACTTAATGGGCCCGCTAACAGCGCGATTTGCTGGTGACCCAATGCGACCAGATGCTCCAC
GCCCAGTCGCGTACCGTCTTCATGGGAGAAAATAATACTGTTGATGGGTGTCTGGTCAGAGA
CATCAAGAAATAACGCCGGAACATTAGTGCAGGCAGCTTCCACAGCAATGGCATCCTGGTCA
TCCAGCGGATAGTTAATGATCAGCCCACTGACGCGTTGCGCGAGAAGATTGTGCACCGCCGC
TTTACAGGCTTCGACGCCGCTTCGTTCTACCATCGACACCACCACGCTGGCACCCAGTTGATC
GGCGCGAGATTTAATCGCCGCGACAATTTGCGACGGCGCGTGCAGGGCCAGACTGGAGGTG
GCAACGCCAATCAGCAACGACTGTTTGCCCGCCAGTTGTTGTGCCACGCGGTTGGGAATGTA
ATTCAGCTCCGCCATCGCCGCTTCCACTTTTTCCCGCGTTTTCGCAGAAACGTGGCTGGCCTG
GTTCACCACGCGGGAAACGGTCTGATAAGAGACACCGGCATACTCTGCGACATCGTATAACG
TTACTGGTTTCACATTCACCACCCTGAATTGACTCTCTTCCGGGCGCTATCATGCCATACCGC
GAAAGGTTTTGCGCCATTCGATGGTGTCCGGGATCTCGACGCTCTCCCTTATGCGACTCCTGC
ATTAGGAAATTAATACGACTCACTATA

r. pDuet::ClpB

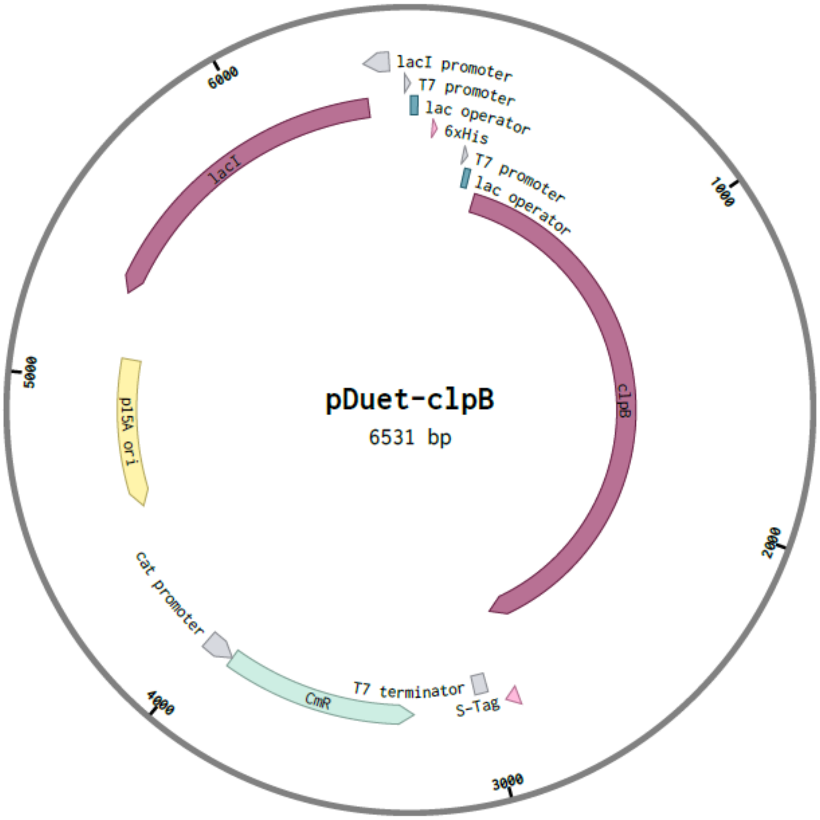

GGGGAATTGTGAGCGGATAACAATTCCCCTGTAGAAATAATTTTGTTTAACTTTAATAAGGA
GATATACCATGGGCAGCAGCCATCACCATCATCACCACAGCCAGGATCCGAATTCGAGCTCG
GCGCGCCTGCAGGTCGACAAGCTTGCGGCCGCATAATGCTTAAGTCGAACAGAAAGTAATCG
TATTGTACACGGCCGCATAATCGAAATTAATACGACTCACTATAGGGGAATTGTGAGCGGAT
AACAATTCCCCATCTTAGTATATTAGTTAAGTATAAGAAGGAGATATACATATGATGCGTCT
GGATCGTCTTACTAATAAATTCCAGCTTGCTCTTGCCGATGCCCAATCACTTGCACTCGGGCA
CGACAACCAATTTATCGAACCACTTCATTTAATGAGCGCCCTGCTGAATCAGGAAGGGGGTT
CGGTTAGTCCTTTATTAACATCCGCTGGCATAAATGCTGGCCAGTTGCGCACAGATATCAATC
AGGCATTAAATCGTTTACCGCAGGTTGAAGGTACTGGTGGTGATGTCCAGCCATCACAGGAT
CTGGTGCGCGTTCTTAATCTTTGCGACAAGCTGGCGCAAAAACGTGGTGATAACTTTATCTCG
TCAGAACTGTTCGTTCTGGCGGCACTTGAGTCTCGCGGCACGCTGGCCGACATCCTGAAAGC
AGCAGGGGCGACCACCGCCAACATTACTCAAGCGATTGAACAAATGCGTGGAGGTGAAAGC
GTGAACGATCAAGGTGCTGAAGACCAACGTCAGGCTTTGAAAAAATATACCATCGACCTTAC
CGAACGAGCCGAACAGGGCAAACTCGATCCGGTGATTGGTCGTGATGAAGAAATTCGCCGT
ACCATTCAGGTGCTGCAACGTCGTACTAAAAATAACCCGGTACTGATTGGTGAACCCGGCGT
CGGTAAAACTGCCATCGTTGAAGGTCTGGCGCAGCGTATTATCAACGGCGAAGTGCCGGAAG

GGTTGAAAGGCCGCCGGGTACTGGCGCTGGATATGGGCGCGCTGGTGGCTGGGGCGAAATA
TCGCGGTGAGTTTGAAGAACGTTTAAAAGGCGTGCTTAACGATCTTGCCAAACAGGAAGGCA
ACGTCATCCTATTTATCGACGAATTACATACCATGGTCGGCGCGGGTAAAGCCGATGGCGCA
ATGGACGCCGGAAACATGCTGAAACCGGCGCTGGCGCGTGGTGAATTGCACTGCGTAGGTGC
CACGACGCTTGACGAATATCGCCAGTACATTGAAAAAGATGCTGCGCTGGAACGTCGTTTCC
AGAAAGTGTTTGTTGCCGAGCCTTCTGTTGAAGATACCATTGCGATTCTGCGTGGCCTGAAAG
AACGTTACGAATTGCACCACCATGTGCAAATTACTGACCCGGCAATTGTTGCAGCGGCGACG
TTGTCTCATCGCTACATTGCTGACCGTCAGCTGCCGGATAAAGCCATCGACCTGATCGATGAA
GCAGCATCCAGCATTCGTATGCAGATTGACTCAAAACCAGAAGAACTCGACCGACTCGATCG
TCGTATCATCCAGCTCAAACTGGAACAACAGGCGTTAATGAAAGAGTCTGATGAAGCCAGTA
AAAAACGTCTGGATATGCTCAACGAAGAACTGAGCGACAAAGAACGTCAGTACTCCGAGTT
AGAAGAAGAGTGGAAAGCAGAGAAGGCATCGCTTTCTGGTACGCAGACCATTAAAGCGGAA
CTGGAACAGGCGAAAATCGCTATTGAACAGGCTCGCCGTGTGGGGGACCTGGCGCGGATGTC
TGAACTGCAATACGGCAAAATCCCGGAACTGGAAAAGCAACTGGAAGCCGCAACGCAGCTC
GAAGGCAAAACTATGCGTCTGTTGCGTAATAAAGTGACCGACGCCGAAATTGCTGAAGTGCT
GGCGCGTTGGACGGGGATTCCGGTTTCTCGCATGATGGAAAGCGAGCGCGAAAAACTGCTGC
GTATGGAGCAAGAACTGCACCATCGCGTAATTGGTCAGAACGAAGCGGTTGATGCGGTATCT
AACGCTATTCGTCGTAGCCGTGCGGGGCTGGCGGATCCAAATCGCCCGATTGGTTCATTCCTG
TTCCTCGGCCCAACTGGTGTGGGGAAAACAGAGCTTTGTAAGGCGCTGGCGAACTTTATGTT
TGATAGCGACGAGGCGATGGTCCGTATCGATATGTCCGAGTTTATGGAGAAACACTCGGTGT
CTCGTTTGGTTGGTGCGCCTCCGGGATATGTCGGTTATGAAGAAGGTGGCTACCTGACCGAA
GCGGTGCGTCGTCGTCCGTATTCCGTCATCCTGCTGGATGAAGTGGAAAAAGCGCATCCGGA
TGTCTTCAACATTCTGTTGCAGGTACTGGATGATGGGCGTCTGACTGACGGGCAAGGGAGAA
CGGTCGACTTCCGTAATACGGTCGTCATTATGACCTCTAACCTCGGTTCCGATCTGATTCAGG
AACGCTTCGGTGAACTGGATTATGCGCACATGAAAGAGCTGGTGCTCGGTGTGGTAAGCCAT
AACTTCCGTCCGGAATTCATTAACCGTATCGATGAAGTGGTGGTCTTCCATCCGCTGGGTGAA
CAGCACATTGCCTCGATTGCGCAGATTCAGTTGAAACGTCTGTACAAACGTCTGGAAGAACG
TGGTTATGAAATCCACATTTCTGACGAGGCGCTGAAACTGCTGAGCGAGAACGGTTACGATC
CGGTCTATGGTGCACGTCCTCTGAAACGTGCAATTCAGCAGCAGATCGAAAACCCGCTGGCA
CAGCAAATACTGTCTGGTGAATTGGTTCCGGGTAAAGTGATTCGCCTGGAAGTTAATGAAGA
CCGGATTGTCGCCGTCCAGTAACTCGAGTCTGGTAAAGAAACCGCTGCTGCGAAATTTGAAC
GCCAGCACATGGACTCGTCTACTAGCGCAGCTTAATTAACCTAGGCTGCTGCCACCGCTGAG
CAATAACTAGCATAACCCCTTGGGGCCTCTAAACGGGTCTTGAGGGGTTTTTTGCTGAAACCT
CAGGCATTTGAGAAGCACACGGTCACACTGCTTCCGGTAGTCAATAAACCGGTAAACCAGCA

ATAGACATAAGCGGCTATTTAACGACCCTGCCCTGAACCGACGACCGGGTCGAATTTGCTTT
CGAATTTCTGCCATTCATCCGCTTATTATCACTTATTCAGGCGTAGCACCAGGCGTTTAAGGG
CACCAATAACTGCCTTAAAAAAATTACGCCCCGCCCTGCCACTCATCGCAGTACTGTTGTAAT
TCATTAAGCATTCTGCCGACATGGAAGCCATCACAGACGGCATGATGAACCTGAATCGCCAG
CGGCATCAGCACCTTGTCGCCTTGCGTATAATATTTGCCCATAGTGAAAACGGGGGCGAAGA
AGTTGTCCATATTGGCCACGTTTAAATCAAAACTGGTGAAACTCACCCAGGGATTGGCTGAG
ACGAAAAACATATTCTCAATAAACCCTTTAGGGAAATAGGCCAGGTTTTCACCGTAACACGC
CACATCTTGCGAATATATGTGTAGAAACTGCCGGAAATCGTCGTGGTATTCACTCCAGAGCG
ATGAAAACGTTTCAGTTTGCTCATGGAAAACGGTGTAACAAGGGTGAACACTATCCCATATC
ACCAGCTCACCGTCTTTCATTGCCATACGGAACTCCGGATGAGCATTCATCAGGCGGGCAAG
AATGTGAATAAAGGCCGGATAAAACTTGTGCTTATTTTTCTTTACGGTCTTTAAAAAGGCCGT
AATATCCAGCTGAACGGTCTGGTTATAGGTACATTGAGCAACTGACTGAAATGCCTCAAAAT
GTTCTTTACGATGCCATTGGGATATATCAACGGTGGTATATCCAGTGATTTTTTTCTCCATTTT
AGCTTCCTTAGCTCCTGAAAATCTCGATAACTCAAAAAATACGCCCGGTAGTGATCTTATTTC
ATTATGGTGAAAGTTGGAACCTCTTACGTGCCGATCAACGTCTCATTTTCGCCAAAAGTTGGC
CCAGGGCTTCCCGGTATCAACAGGGACACCAGGATTTATTTATTCTGCGAAGTGATCTTCCGT
CACAGGTATTTATTCGGCGCAAAGTGCGTCGGGTGATGCTGCCAACTTACTGATTTAGTGTAT
GATGGTGTTTTTGAGGTGCTCCAGTGGCTTCTGTTTCTATCAGCTGTCCCTCCTGTTCAGCTAC
TGACGGGGTGGTGCGTAACGGCAAAAGCACCGCCGGACATCAGCGCTAGCGGAGTGTATAC
TGGCTTACTATGTTGGCACTGATGAGGGTGTCAGTGAAGTGCTTCATGTGGCAGGAGAAAAA
AGGCTGCACCGGTGCGTCAGCAGAATATGTGATACAGGATATATTCCGCTTCCTCGCTCACT
GACTCGCTACGCTCGGTCGTTCGACTGCGGCGAGCGGAAATGGCTTACGAACGGGGCGGAG
ATTTCCTGGAAGATGCCAGGAAGATACTTAACAGGGAAGTGAGAGGGCCGCGGCAAAGCCG
TTTTTCCATAGGCTCCGCCCCCCTGACAAGCATCACGAAATCTGACGCTCAAATCAGTGGTGG
CGAAACCCGACAGGACTATAAAGATACCAGGCGTTTCCCCTGGCGGCTCCCTCGTGCGCTCT
CCTGTTCCTGCCTTTCGGTTTACCGGTGTCATTCCGCTGTTATGGCCGCGTTTGTCTCATTCCA
CGCCTGACACTCAGTTCCGGGTAGGCAGTTCGCTCCAAGCTGGACTGTATGCACGAACCCCC
CGTTCAGTCCGACCGCTGCGCCTTATCCGGTAACTATCGTCTTGAGTCCAACCCGGAAAGAC
ATGCAAAAGCACCACTGGCAGCAGCCACTGGTAATTGATTTAGAGGAGTTAGTCTTGAAGTC
ATGCGCCGGTTAAGGCTAAACTGAAAGGACAAGTTTTGGTGACTGCGCTCCTCCAAGCCAGT
TACCTCGGTTCAAAGAGTTGGTAGCTCAGAGAACCTTCGAAAAACCGCCCTGCAAGGCGGTT
TTTTCGTTTTCAGAGCAAGAGATTACGCGCAGACCAAAACGATCTCAAGAAGATCATCTTAT
TAATCAGATAAAATATTTCTAGATTTCAGTGCAATTTATCTCTTCAAATGTAGCACCTGAAGT
CAGCCCCATACGATATAAGTTGTAATTCTCATGTTAGTCATGCCCCGCGCCCACCGGAAGGA

GCTGACTGGGTTGAAGGCTCTCAAGGGCATCGGTCGAGATCCCGGTGCCTAATGAGTGAGCT
AACTTACATTAATTGCGTTGCGCTCACTGCCCGCTTTCCAGTCGGGAAACCTGTCGTGCCAGC
TGCATTAATGAATCGGCCAACGCGCGGGGAGAGGCGGTTTGCGTATTGGGCGCCAGGGTGGT
TTTTCTTTTCACCAGTGAGACGGGCAACAGCTGATTGCCCTTCACCGCCTGGCCCTGAGAGAG
TTGCAGCAAGCGGTCCACGCTGGTTTGCCCCAGCAGGCGAAAATCCTGTTTGATGGTGGTTA
ACGGCGGGATATAACATGAGCTGTCTTCGGTATCGTCGTATCCCACTACCGAGATGTCCGCA
CCAACGCGCAGCCCGGACTCGGTAATGGCGCGCATTGCGCCCAGCGCCATCTGATCGTTGGC
AACCAGCATCGCAGTGGGAACGATGCCCTCATTCAGCATTTGCATGGTTTGTTGAAAACCGG
ACATGGCACTCCAGTCGCCTTCCCGTTCCGCTATCGGCTGAATTTGATTGCGAGTGAGATATT
TATGCCAGCCAGCCAGACGCAGACGCGCCGAGACAGAACTTAATGGGCCCGCTAACAGCGC
GATTTGCTGGTGACCCAATGCGACCAGATGCTCCACGCCCAGTCGCGTACCGTCTTCATGGG
AGAAAATAATACTGTTGATGGGTGTCTGGTCAGAGACATCAAGAAATAACGCCGGAACATTA
GTGCAGGCAGCTTCCACAGCAATGGCATCCTGGTCATCCAGCGGATAGTTAATGATCAGCCC
ACTGACGCGTTGCGCGAGAAGATTGTGCACCGCCGCTTTACAGGCTTCGACGCCGCTTCGTTC
TACCATCGACACCACCACGCTGGCACCCAGTTGATCGGCGCGAGATTTAATCGCCGCGACAA
TTTGCGACGGCGCGTGCAGGGCCAGACTGGAGGTGGCAACGCCAATCAGCAACGACTGTTTG
CCCGCCAGTTGTTGTGCCACGCGGTTGGGAATGTAATTCAGCTCCGCCATCGCCGCTTCCACT
TTTTCCCGCGTTTTCGCAGAAACGTGGCTGGCCTGGTTCACCACGCGGGAAACGGTCTGATA
AGAGACACCGGCATACTCTGCGACATCGTATAACGTTACTGGTTTCACATTCACCACCCTGA
ATTGACTCTCTTCCGGGCGCTATCATGCCATACCGCGAAAGGTTTTGCGCCATTCGATGGTGT
CCGGGATCTCGACGCTCTCCCTTATGCGACTCCTGCATTAGGAAATTAATACGACTCACTATA

3. Non-cropped Western Blots

a. Production of rhGALNS in *E. coli* BL21(DE3) using different promoters

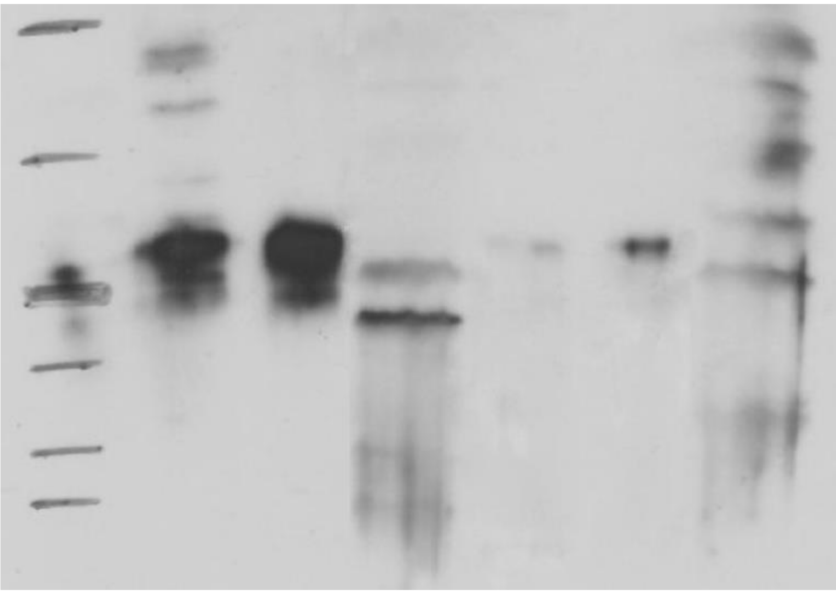

b. Dynamics of the expression of rhGALNS under the control of the promoter *proU*$_{mod}$.

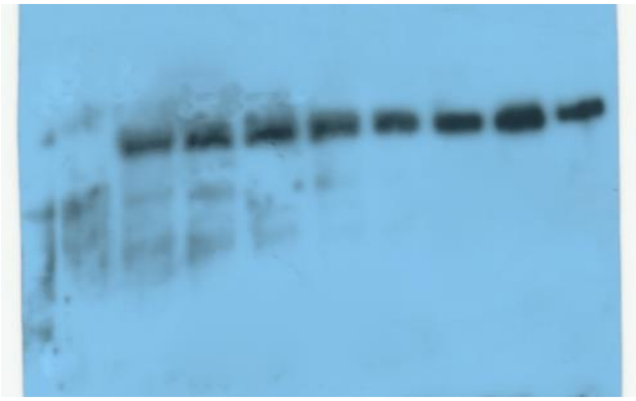

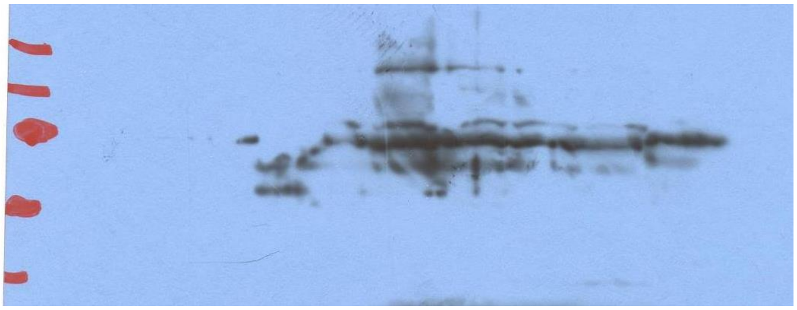

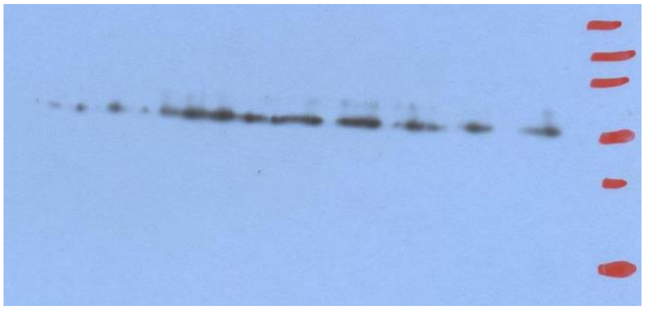

c. Production of rhGALNS using the promoters *tac* and *proU*$_{mod}$, with two different *E. coli* strains: BL21(DE3) and SHuffle®

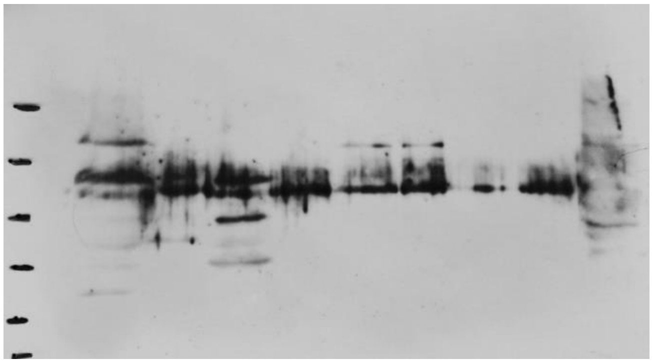

4. Purification via affinity chromatography

Cell lysates were applied to a Glutathione Sepharose 4B with peristaltic pump, under manufacturer instructions (GE Healthcare). In short, the column was equilibrated with approximately ten column volumes of PBS (140 mM NaCl, 2.7 mM KCl, 10 mM Na2 HPO4, 1.8 mM KH2PO4, pH 7.3). Then, the column was incubated for 1 hour with the sample at room temperature, followed by a washing step with ten column volumes of PBS pH 7.3 until no material appears in the flow-through. Finally, the proteins were eluted with elution buffer (50 mM Tris-HCl, 10 mM reduced glutathione). The pH was continuously increased from pH 8.0. Fractions of 0.5 ml were collected and visualized using a SDS-PAGE.

The following table indicates the protein amounts obtained after each purification step.

	mL	Concentration (mg/mL)	Total protein mg	Bound protein (mg/ml)	Bound protein after washing (mg/ml)	Bound protein after washing + eluted (mg/ml)
**Protein load**	5	24.870	124.350			
**Flow-through**	5	18.470	92.850	32.000	14.325	15.268
**Wash**	50	0.354	17.675			
**Eluted**	25	0.034	0.9425			

After the samples were visualized in a SDS-PAGE (Figure shown below), it was evident that the protein fusion was not expressed. Instead, a band corresponding to the GST peptide (26 kDa) was found in the different fractions, prominent in the 20th fraction. These samples were tested for enzyme activity but it was not present.

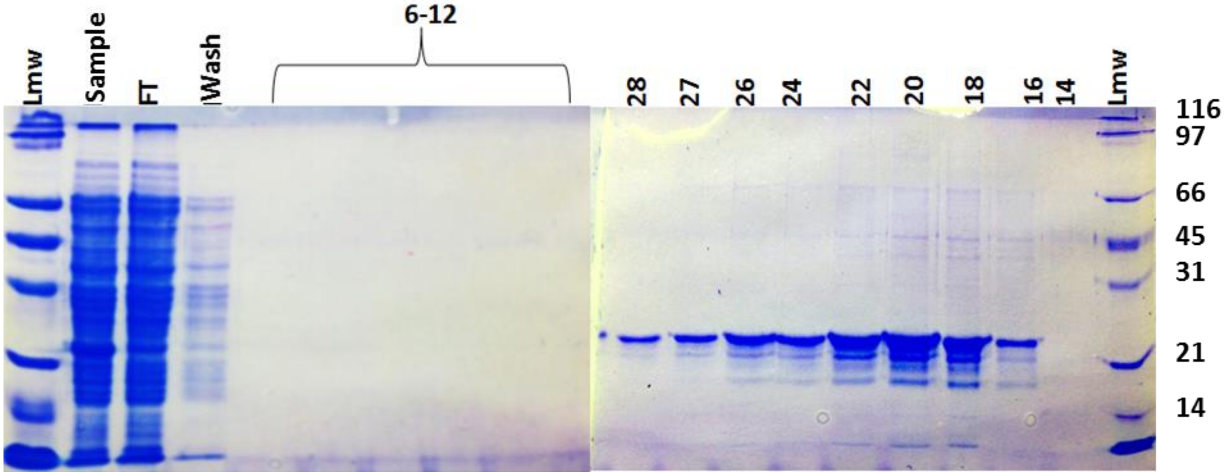

5. Indirect Enzyme-Linked Immunosorbent Assay (ELISA)

An indirect ELISA was carried out in order to validate the extracellular amounts rhGALNS, according to the protocol described by Rodriguez *et. al.* 2010. A calibration curve between 0 and 1 µg/ml was created using a recombinant human GALNS enzyme produced in CHO cells (stock concentration of 551 µg/ml). The samples quantified correspond to culture medium of BL21(DE3)/pGEX-5X-GALNSopt after 24 hours cultivation, exposed to 5% (w/v) sucrose, 0.05N NaCl and absence of osmotic stress.

The results indicate (Figure shown below) that the amounts of rhGALNS in the extracellular fraction were larger when the cells were exposed to osmotic stress, either in the presence of sucrose or sodium chloride, with increments of 349% and 265% of the secreted protein, respectively.

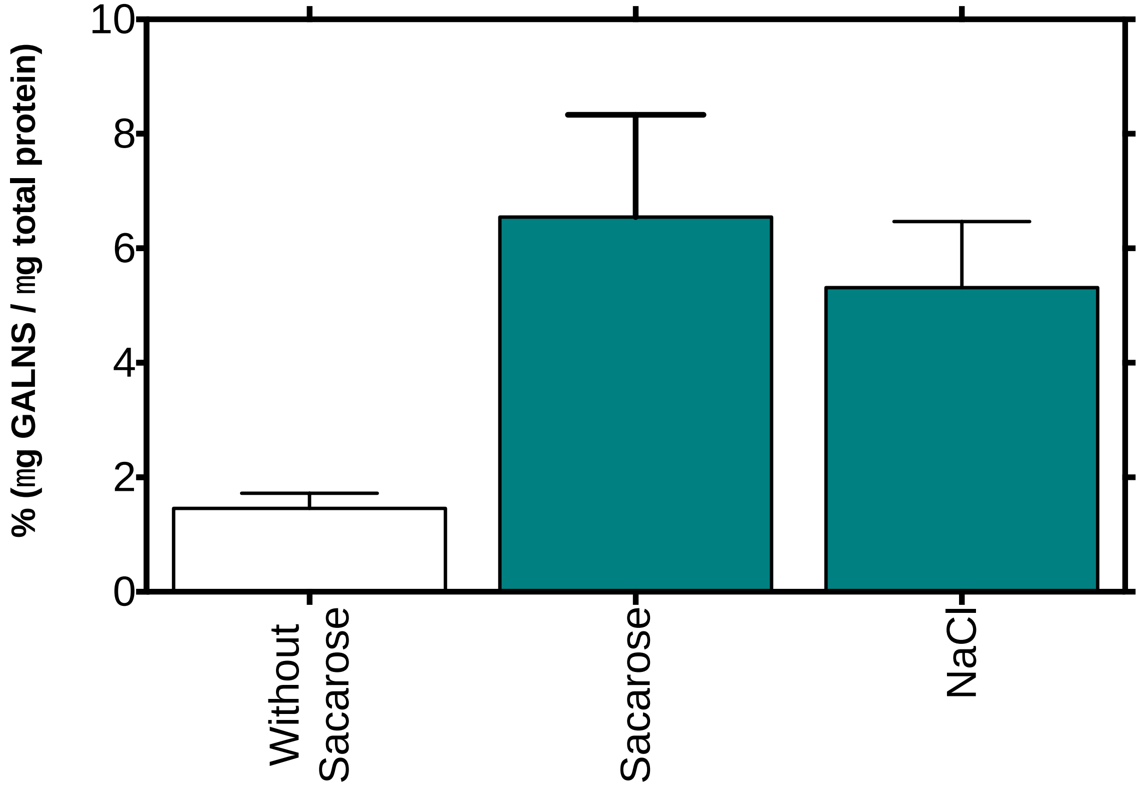

Supplement: Supplementary file 1 — Supplementary Information [file 41598_2017_6367_MOESM1_ESM.pdf]
